# No genetic causal associations between periodontitis and brain atrophy or cognitive impairment: evidence from a comprehensive bidirectional Mendelian randomization study

**DOI:** 10.1186/s12903-024-04367-7

**Published:** 2024-05-16

**Authors:** Zhixing Deng, Jiaming Li, Yuhao Zhang, Yinian Zhang

**Affiliations:** 1 The Second School of Clinical Medicine, Southern Medical University, Guangzhou, China; 2 Department of Neuro-Oncological Surgery, Neurosurgery Center, Zhujiang Hospital of Southern Medical University, Guangzhou, China

**Keywords:** Mendelian randomization analysis, Periodontitis, Brain atrophy, Cognitive impairment

## Abstract

**Background:**

Observational studies have explored the relationships of periodontitis with brain atrophy and cognitive impairment, but these findings are limited by reverse causation, confounders and have reported conflicting results. Our study aimed to investigate the causal associations of periodontitis with brain atrophy and cognitive impairment through a comprehensive bidirectional Mendelian randomization (MR) research.

**Methods:**

We incorporated two distinct genome-wide association study (GWAS) summary datasets as an exploration cohort and a replication cohort for periodontitis. Four and eight metrics were selected for the insightful evaluation of brain atrophy and cognitive impairment, respectively. The former involved cortical thickness and surface area, left and right hippocampal volumes, with the latter covering assessments of cognitive performance, fluid intelligence scores, prospective memory, and reaction time for mild cognitive impairment to Alzheimer's disease (AD), Lewy body dementia, vascular dementia and frontotemporal dementia for severe situations. Furthermore, supplementary analyses were conducted to examine the associations between the longitudinal rates of change in brain atrophy and cognitive function metrics with periodontitis. The main analysis utilized the inverse variance weighting (IVW) method and evaluated the robustness of the results through a series of sensitivity analyses. For multiple tests, associations with *p*-values < 0.0021 were considered statistically significant, while *p*-values ≥ 0.0021 and < 0.05 were regarded as suggestive of significance.

**Results:**

In the exploration cohort, forward and reverse MR results revealed no causal associations between periodontitis and brain atrophy or cognitive impairment, and only a potential causal association was found between AD and periodontitis (IVW: OR = 0.917, 95% CI from 0.845 to 0.995, *P* = 0.038). Results from the replication cohort similarly corroborated the absence of a causal relationship. In the supplementary analyses, the longitudinal rates of change in brain atrophy and cognitive function were also not found to have causal relationships with periodontitis.

**Conclusions:**

The MR analyses indicated a lack of substantial evidence for a causal connection between periodontitis and both brain atrophy and cognitive impairment.

**Supplementary Information:**

The online version contains supplementary material available at 10.1186/s12903-024-04367-7.

## Background

Periodontitis is a chronic, multifactorial inflammatory disease arising from the dysregulation of interactions between oral microorganisms and the host's immune response. This imbalance can culminate in a persistent, detrimental inflammatory reaction leading to tissue destruction [1]. It affects approximately half of the adult population, with a significant 10% experiencing its severe form. Its prevalence, coupled with potential links to severe diseases such as cardiovascular disease, cancer, and Alzheimer's disease (AD), places immense pressure on the global healthcare structure [2–5]. Simultaneously, cognitive impairment stands as a major public health concern worldwide [6]. Several studies have delved into the possible connection between periodontitis and cognitive decline. For instance, a 5-year study involving 179 senior men and women revealed a link between severe periodontitis and mild cognitive impairment [7]. Another community-based study observed a modest association of periodontitis with minor cognitive ailments in both black and white participants [8]. However, the most recent meta-analysis suggested a potential bias in evidence, making it difficult to determine whether periodontitis is a genuine risk factor for age-induced cognitive decline [9]. Furthermore, recent studies have sparked interest in the relationship between periodontitis and brain health. Experimental data from animal models indicated a potential association between the inflammatory reactions triggered by periodontitis, particularly in response to its primary pathogen, *Porphyromonas*, and the pathogenesis of AD [10]. While one study suggested that treating periodontitis can mitigate AD-related brain atrophy, [11] another study found no association between the severity of periodontitis and brain morphological changes [12]. Importantly, these studies are observational, and their results remain contentious. Given the current state of research, we cannot definitively establish a causal link between periodontitis and cognitive impairment or brain health.

Mendelian randomization (MR) is a method of assessing causality and limiting confounding-induced bias by using genetic variation closely associated with exposure as an instrumental variable (IV) [13]. We hypothesized that there is a bidirectional causal relationship of periodontitis with brain atrophy and cognitive impairment. In this study, we sought to elucidate this potential causative association by employing MR analysis. For brain atrophy, an organic brain tissue lesion associated with various neurodegenerative pathologies, assessment can be conducted through specific imaging methods, including magnetic resonance imaging (MRI). Typically, areas most significantly affected by atrophy comprise the cerebral cortex along with select subcortical structures, clinically valuable for measuring the extent of atrophy [14]. Therefore, our study incorporated cortical surface area, cortical thickness, and left and right hippocampal volumes as indicators of brain atrophy. As for cognitive impairment, a clinical syndrome characterized by cognitive decline, encompasses a wide range of symptoms. To conduct a comprehensive analysis, we chose phenotypes ranging from mild cognitive decline to severe impairment. The former included cognitive performance, fluid intelligence score, prospective memory, and reaction time, which have proven to be robust assessments [15]. The latter included common types of dementia: AD (all, early-onset, late-onset), Lewy body dementia, vascular dementia, and frontotemporal dementia. In addition, considering that the MR treats the time-varying trait as a single measurement, we additionally added relevant dataset that reflect the rate of change in brain atrophy and cognitive function over time as exposures to analyze their association with periodontitis.

## Methods

### Study design

**Fig. 1:**
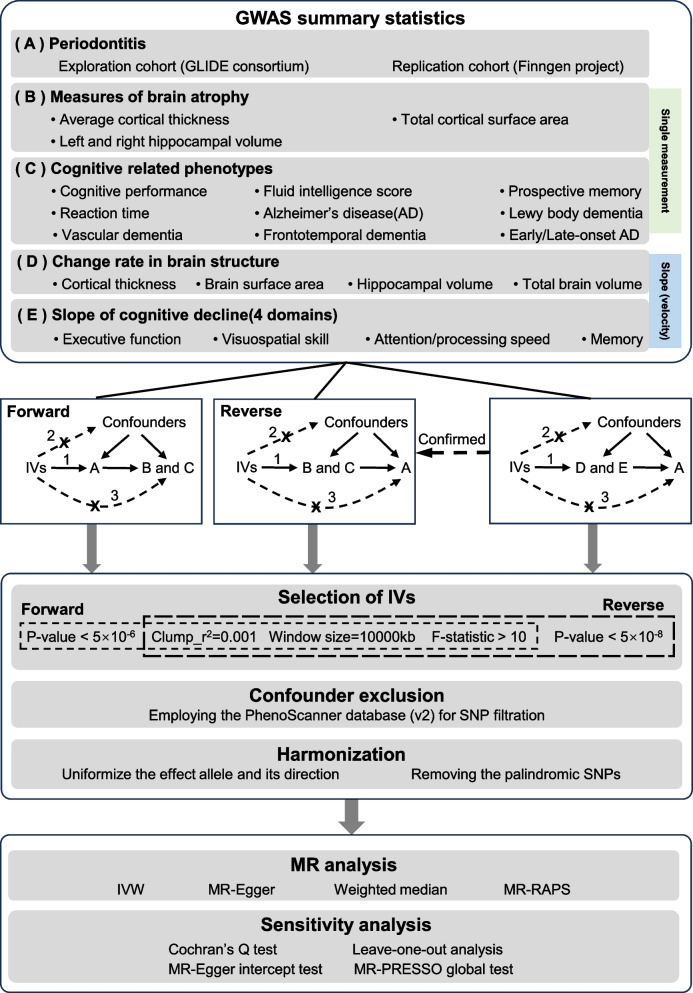
Research flowchart

We conducted a two-cohort, bidirectional MR analysis and the specific flow of the study is depicted in Fig. 1. To illustrate a potential causal effect between exposure and outcome through MR analysis, the selected single nucleotide polymorphisms (SNPs) are required to comply with three major assumptions: 1. Correlation: the selected IVs are robustly correlated with exposure; 2. Independence: the IVs are irrelevant to the confounders associated with the outcome-exposure association; 3. Exclusion restriction: SNPs affect outcomes only through exposure but not through other pathways [16, 17]. All the genome-wide association study (GWAS) data involved in this study are publicly accessible, the participants were predominantly of European descent, and the original study was ethically approved.

### GWAS summary data

Table 1 displayed the basic information of all the cohorts we incorporated, including sample size, age ranges, etc. We described the adjustments for confounders made by each original GWAS and the supplementary explanation for measurements of traits in Table S1.

**Table 1:** Basic information on all GWAS included in the study

**Traits**	**Consortium**	**Total**	**Ncase**	**Ncontrol**	**Population**	**Age**
Periodontitis	GLIDE	45,563	17,353	28,210	European	-
Periodontitis	FinnGen	263,668	4,434	259,234	European	-
Cerebral cortex	ENIGMA	51,665	4,809	46,856	European (94%)	3-91
Hippocampal volume	UK Biobank	36,778	-	-	European	40-82
Cognitive performance	COGENT and UK Biobank	34,652	-	-	European	16-102
Fluid intelligence score	UK Biobank	149,051	-	-	European	-
Prospective memory	UK Biobank	152,605	-	-	European	-
Reaction time	CCACE	330,069	-	-	European	8-102
Alzheimer’s disease (AD)	-	487,511	85,934	401,577	European	53-91
Early-onset AD	FinnGen	185,204	1,451	183,753	European	-
Late-onset AD	FinnGen	191,061	7,308	183,753	European	-
Vascular dementia	UK Biobank	411,277	444	410833	European	-
Lewy body dementia	-	6,618	2,591	4,027	European	39-89
Frontotemporal dementia	FinnGen	392,592	129	392,463	European	-
Change rate in brain structure	ENIGMA	15,640	2250	13390	European (>99%)	4-99
Slope of cognitive decline	-	1145	179	966	European	66-84

#### Periodontitis

For the study of periodontitis, we employed two different sources of data, designating one as the exploration cohort and the other as the replication cohort. GWAS summary statistics obtained from the Gene-Lifestyle Interactions in Dental Endpoints (GLIDE) consortium [18]served as the exploration cohort, which included seven European-descent cohort studies (17,353 cases; 28,210 controls). Among them, four were diagnosed based on the Centers for Disease Control and Prevention/American Academy of Periodontology (CDC/AAP) criterion, two were confirmed through the Community Periodontal Index (CPI), and one was confirmed according to the patient’s self-reports. Additionally, the replication cohort comprises periodontitis summary data from the FinnGen project, [19] which includes 263,668 individuals (4,434 cases; 259,234 controls). Within the FinnGen research program, participants were stratified based on distinct defining events. For the diagnosis of patients with chronic periodontitis, identification was conducted through screening with periodontitis-related ICD codes, including ICD-8:5234, ICD-9:5234, and ICD-10: K05.30, K05.31. The controls were individuals devoid of diseases affecting the oral cavity, salivary glands, or jaw.

#### Measurements of brain atrophy

The mean cortical thickness (mm) and total cortical surface area (mm^2^) were derived from a meta-analysis of genome-wide T1-weighted MRI of the brain by the Enhancing Neuroimaging Genetics through Meta-analysis (ENIGMA) consortium [20]. It measured the brain MRI data of 51,665 participants from 60 cohorts to characterize genetic variants affecting the structure of the cerebral cortex, with a major population of Europeans (approximately 94%). Genetic data for hippocampal volumes were sourced from a European population-based GWAS carried out by Fürtjes [21], which modeled the overall dimensions of variation in the morphological structure of the human brain. A total of 36,778 people with T1 and T2 brain imaging information were involved in the study, all from the UK Biobank.

#### Cognitive related traits

The summary statistics of genetic association with cognitive performance were taken from a GWAS encompassing 257,841 individuals. This research integrated previously published studies on general cognitive performance from the Cognitive Genomics Consortium (COGENT) with the UK Biobank’s new GWAS findings on cognitive performance, explored through sample size-weighted meta-analyses [22]. GWAS data for fluid intelligence score (*N* = 149,051) and prospective memory (*N* = 152,605) were obtained from the UK Biobank, which were evaluated by fluid intelligence questions and an image test, respectively [23]. The data for reaction time (*N* = 330,069) were derived from a study on genetic loci for general cognitive function carried out by the Center for Cognitive Aging and Cognitive Epidemiology (CCACE) at the University of Edinburgh [24]. This metric was based on the cumulative time that participants correctly completed card matching.

We sourced AD-related GWAS data from a two-stage, multi-cohort meta-analysis that revealed a novel genetic signature for AD and its associated dementias [25]. The data included in our study consisted of 85,934 AD cases or proxy cases and 401,577 control samples. Additionally, we also obtained GWAS for early-onset and late-onset AD separately from the FinnGen project to perform a complementary analysis of AD, considering the significant differences in genetic background of the two. To avoid population overlap, we only conducted it in the exploration cohort of periodontitis. GWAS dataet for vascular dementia were accessed from the UK Biobank with a phenotype code of 290.16, consisting of 444 cases and 410,833 controls [26]. Furthermore, we incorporated the largest whole-genome sequencing study of Lewy body dementia to date (2,591 cases; 4,027 controls), which identified novel loci associated with its pathogenesis [27]. Frontotemporal dementia (FTD) GWAS summary data were retrieved from the FinnGen project (129 cases; 392,463 controls) [19]. Notably, the FTD was only used in the exploration cohort for periodontitis to avoid a high overlap between exposure and outcome samples.

#### Measure of time-varying trait

As the aforementioned GWAS on measurements of brain atrophy and mild cognitive change were only single-dimensional indicators, when analyzed as exposures they will fail to capture the change rate in features, but the rate of which tends to be highly relevant to the occurrence and progression of the disease and should not be ignored. Therefore, we employed the specific GWAS for evaluating the rate of change in brain volume and cognitive decline in the reverse analyses to make our conclusions more robust. Four GWAS relating to the change rate of brain, including cortical thickness, were used in our study. They were obtained from the research of Brouwer et al., which has calculated the rate of change in brain structure from longitudinal MRI data of 15,640 individuals in 40 cohorts [28]. It was the first GWAS of brain morphological changes across the lifespan. GWAS summary data for trajectories of cognitive function over time were taken from the study by Kamboh et al. They investigated intra-individual slopes of decline over time in several cognitive domains within two longitudinal cohorts free of dementia at baseline [29]. We selected GWAS for four domains, including executive function and so on.

### Instrument identification

We implemented a series of selection criteria to identify IVs that fulfill the three main assumptions of the MR analyses. Initially, to ensure a robust correlation between IVs and exposure, we set a threshold of 5 × 10^-6^ for periodontitis and 5 × 10^-8^ for both brain atrophy measurements and cognitive impairment. Specifically, we adjusted the threshold for prospective memory, vascular dementia and the eight time-varying traits to 5 × 10^-6^ to capture enough IVs for follow-up analysis. SNPs with *p*-values below these thresholds were considered as candidates. Next, we removed SNPs with linkage disequilibrium by enforcing the criteria of R2 < 0.001 and a window size of 10,000 kb. Subsequently, target SNPs not presented in the outcome GWAS were discarded, and no proxy SNPs were used in our study. We also queried the Phenoscanner(v2) database to confirm that the selected SNPs were not associated with known confounders [30–32]. Finally, we harmonized the exposure and outcome datasets, ensuring that the effect allele was consistent across both, and excluded palindromic SNPs with intermediate allele frequencies.

Furthermore, we calculated the R^2^ and F statistics for each SNP. The R^2^ indicates the exposure variance explained by each SNP, and the F statistic was used to assess the presence of a weak IV bias in the selected SNPs. Genetic variants with F statistic less than 10 were considered weak instruments and were excluded.

### MR analysis

In this study, inverse-variance weighted (IVW), weighted median, MR Egger, and MR-RAPS (robust adjusted profile score) methods were used for MR analysis. We used IVW as the main analysis method, a weighted calculation method that utilizes the inverse of the variance of each IV as the weight under the premise that all IVs are valid, and has a valid ability to detect causal relationships [33]. The weighted median method provides consistent estimates under the condition that at least half of the instruments in the analysis are effective [34]. MR Egger assumes that all IVs are invalid and provides a consistent causal estimate [35]. MR-RAPS is more robust to weak IV bias [36]. Depending on the specific outcome variables, the results are presented either as a β-value or an odds ratio (OR) accompanied by a 95% confidence interval.

### Sensitivity analyses

To evaluate the influence of potential heterogeneity and pleiotropy on the findings, we undertook sensitivity analyses. The Cochran's Q test of the IVW method was used to assess the heterogeneity of the IVs and if *P* < 0.05, the presence of heterogeneity was indicated [37]. The MR Egger intercept test was deployed to identify potential horizontal pleiotropy, signifying its presence with a *p*-value below 0.05 [35]. The MR pleiotropy residual sum and outlier (MR-PRESSO) test was applied to evaluate the potential pleiotropy and outliers, with functions testing for significant differences in causal estimates before and after correcting for outliers. We also utilized leave-one-out analysis to verify the robustness of the results.

We applied Bonferroni's correction to multiple tests, setting a threshold of *P*<0.0021 (0.05/24) for statistical significance. Correlations with *p*-values ranging between 0.0021 and 0.05 were considered suggestive. All analyses were conducted in R (version 4.1.2) using the following packages: "TwoSampleMR" (version 0.5.7), "MRPRESSO" (version 1.0), "mr.raps" (version 0.4.1), and "MendelianRandomization" (version 0.8.0).

## Results

We obtained SNPs that fulfilled the selection criteria for IVs in the exploration cohort and the replication cohort, respectively. All SNPs exhibited an F-statistic greater than 10, confirming the absence of weak IVs that could potentially skew our results. Table S2 presents the characteristics of all included SNPs. Moreover, an extensive search of the PhenoScanner database revealed no genetic instruments associated with confounders in the forward MR analysis. But in the reverse analysis, we excluded a number of SNPs due to their associations with known confounding factors, such as smoking, obesity/body mass index (BMI), diabetes, and inflammation. Table S3 provides a specific account of these excluded SNPs.

**Fig. 2:**
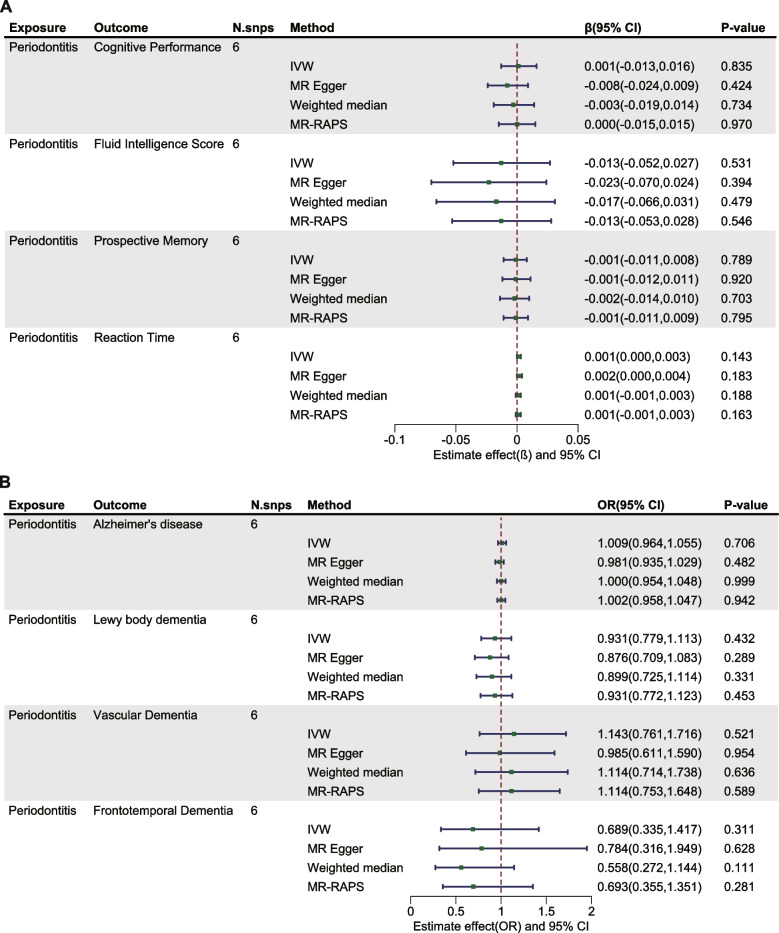
Estimated causal effects between periodontitis and cognitive impairment using different MR methods in exploration cohort. IVW: inverse variance weighted; MR-RAPS: MR- robust adjusted profile score; N.snps: number of SNPs used in MR

**Fig. 3:**
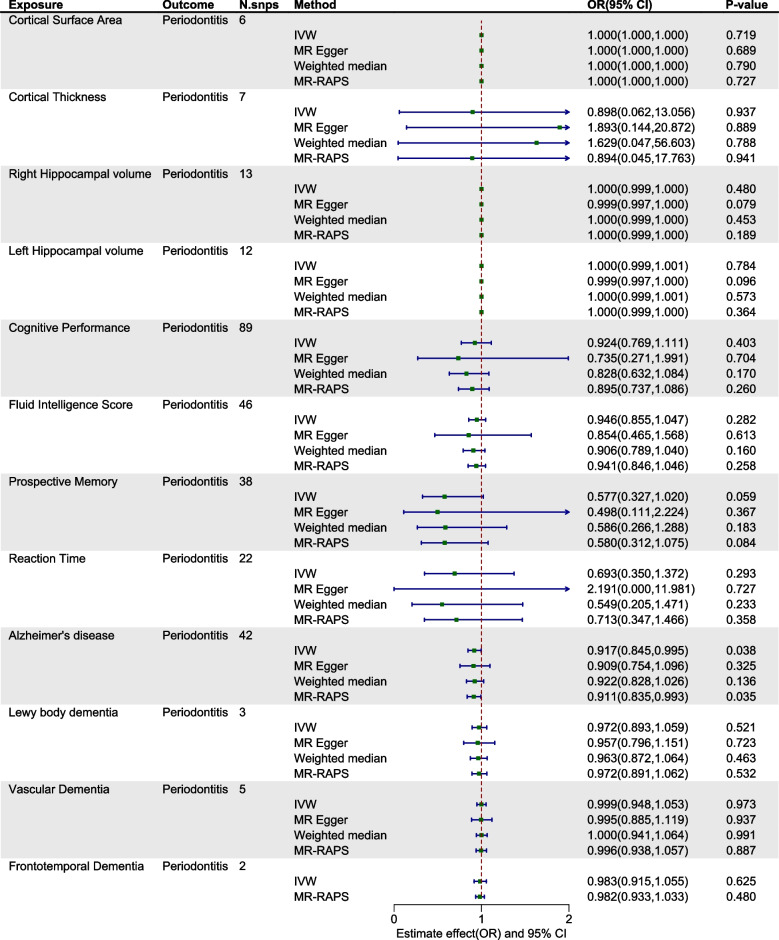
Estimated causal effects between brain atrophy, cognitive impairment and periodontitis using different MR methods in exploration cohort. IVW: inverse variance weighted; MR-RAPS: MR- robust adjusted profile score; N.snps: number of SNPs used in MR

**Fig. 4:**
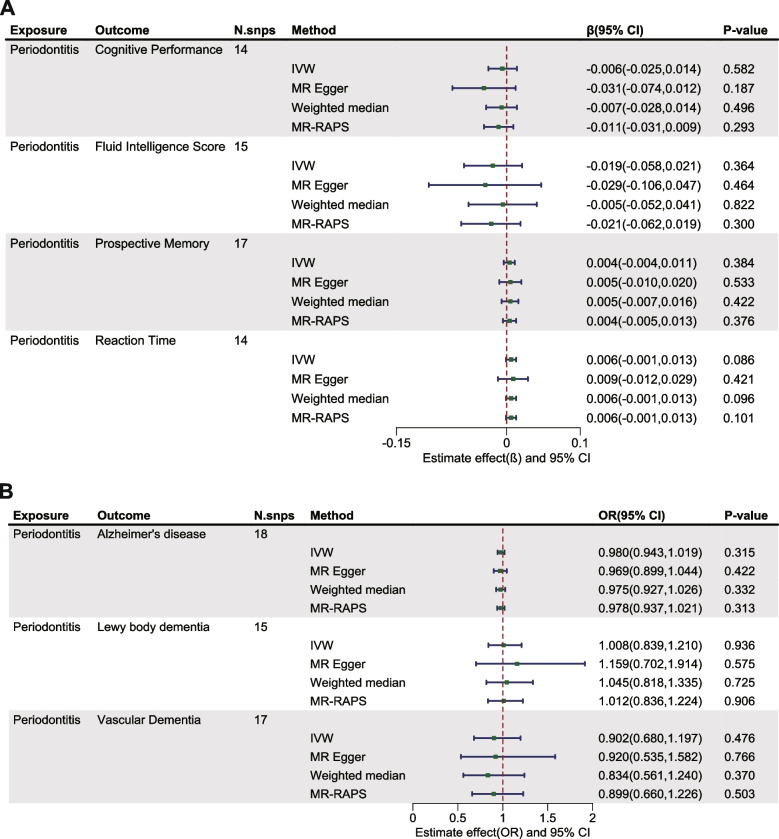
Estimated causal effects between periodontitis and cognitive impairment using different MR methods in replication cohort. IVW: inverse variance weighted; MR-RAPS: MR- robust adjusted profile score; N.snps: number of SNPs used in MR

**Fig. 5:**
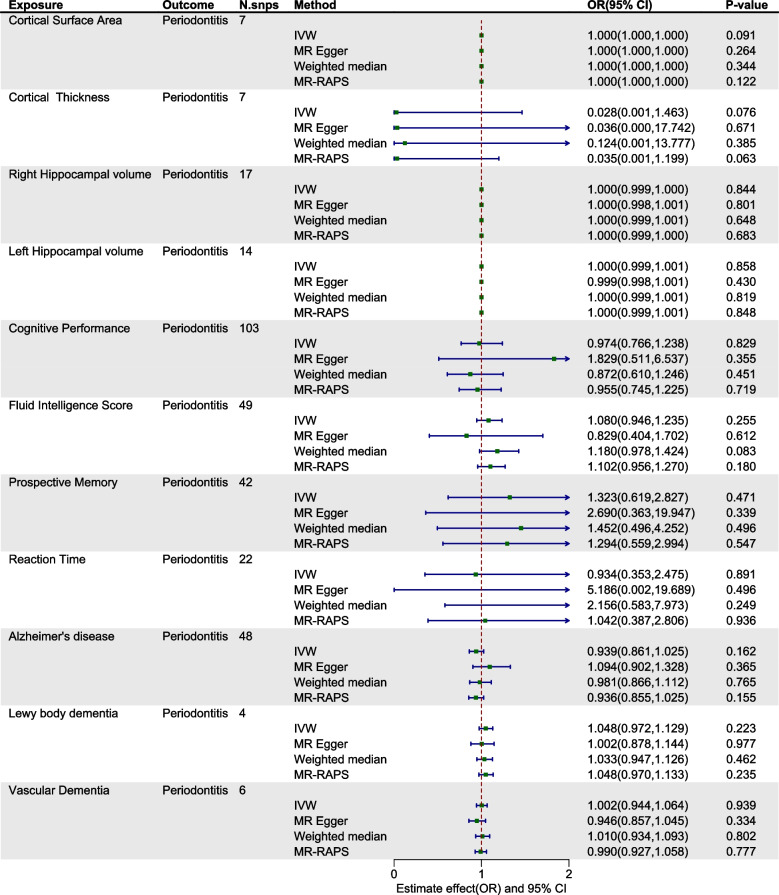
Estimated causal effects between brain atrophy, cognitive impairment and periodontitis using different MR methods in replication cohort. IVW: inverse variance weighted; MR-RAPS: MR- robust adjusted profile score; N.snps: number of SNPs used in MR

**Fig. 6:**
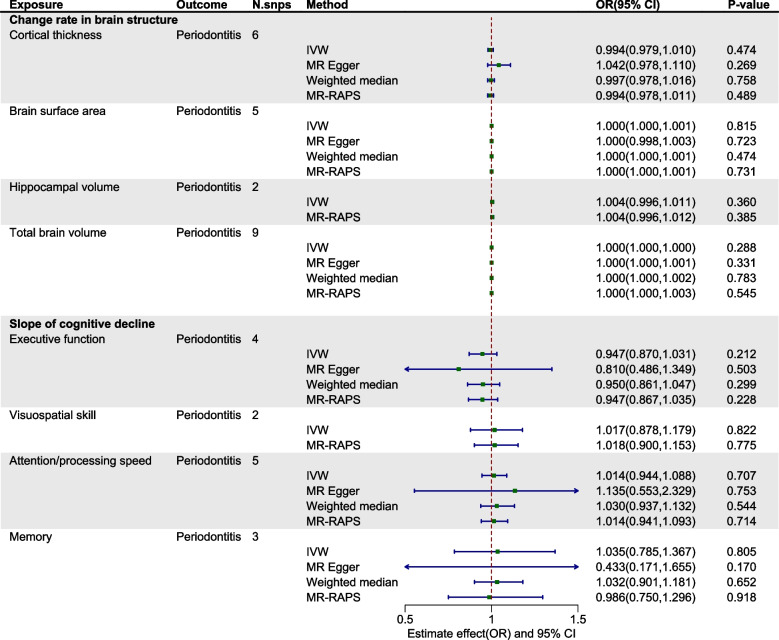
Estimated causal effects between the change rate in brain structure, slope of cognitive decline and periodontitis using different MR methods in exploration cohort. IVW: inverse variance weighted; MR-RAPS: MR- robust adjusted profile score; N.snps: number of SNPs used in MR

**Fig. 7:**
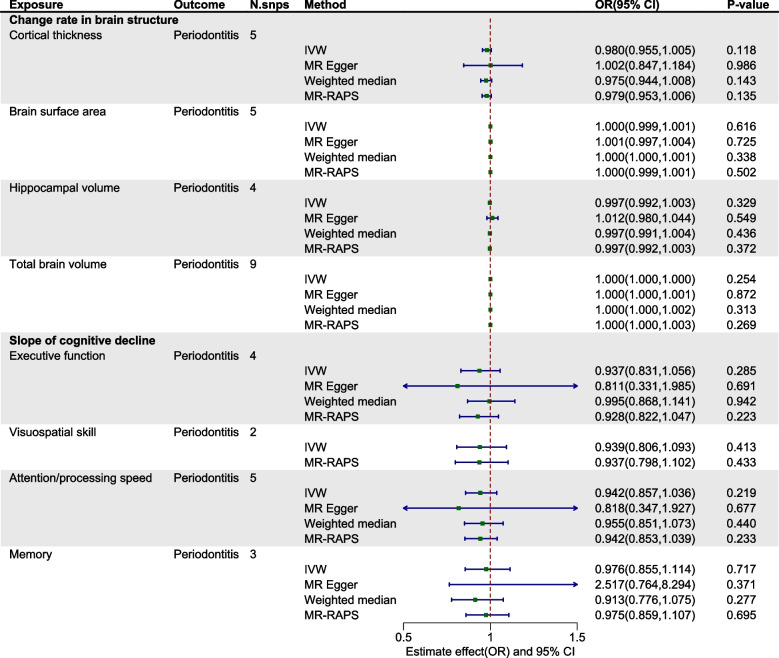
Estimated causal effects between the change rate in brain structure, slope of cognitive decline and periodontitis using different MR methods in replication cohort. IVW: inverse variance weighted; MR-RAPS: MR- robust adjusted profile score; N.snps: number of SNPs used in MR

Within the exploration cohort, the forward MR analysis did not reveal a causal link between periodontitis and either brain atrophy or cognitive impairment. In the reverse analysis, we found a potential causal relationship between Alzheimer's disease and periodontitis (IVW: OR= 0.917, 95% CI from 0.845 to 0.995, *P* = 0.038) (Table 2, Figs. 2 and 3, Figure S1-2). Nevertheless, when considering the aggregate evidence and after applying corrections for multiple testing (*P*=0.038>0.0021), we conservatively concluded that there was no significant causal relationship between brain atrophy, cognitive impairment and periodontitis. For both early-onset and late-onset AD, the results of our analyses in both directions also showed no causal relationship with periodontitis (Figure S3-4). Similarly, in the replication cohort, no causal associations were supported between periodontitis and brain atrophy or cognitive impairment in either direction of the MR analysis (Table 3, Figs. 4 and 5, Figure S5-6). Furthermore, in the supplementary analyses, no causal associations were detected between the change rate of brain structures, the slope of cognitive decline and periodontitis in both two cohorts (Figs. 6 and 7).

**Table 2:** Estimated causal effects between periodontitis and measurements of brain atrophy using different MR methods in exploration cohort

**Exposure**	**Outcome**	**N.snps**	**Method**	**Beta**	**95% CI**	***P*-value**
Periodontitis	Cortical Surface Area	4	IVW	-785.968	-2120.443,548.506	0.248
			MR Egger	-1682.17	-4409.23,1044.89	0.350
			Weighted median	-382.164	-2001.358,1237.03	0.644
			MR-RAPS	-806.826	-2236.143,622.491	0.269
	Cortical Thickness	4	IVW	-0.005	-0.014,0.004	0.301
			MR Egger	-0.007	-0.029,0.014	0.579
			Weighted median	-0.002	-0.013,0.009	0.762
			MR-RAPS	-0.005	-0.015,0.005	0.322
	Right Hippocampal volume	5	IVW	-3.834	-24.075,16.406	0.710
			MR Egger	-4.472	-32.312,23.369	0.774
			Weighted median	-4.617	-28.159,18.925	0.701
			MR-RAPS	-3.851	-24.765,17.062	0.718
	Left Hippocampal volume	5	IVW	-5.224	-24.303,13.856	0.592
			MR Egger	-5.799	-32.036,20.438	0.694
			Weighted median	-3.769	-27.831,20.292	0.759
			MR-RAPS	-5.302	-25.222,14.618	0.602

*IVW* Inverse variance weighted, *MR-RAPS* MR- robust adjusted profile score, *N.snps* Number of SNPs used in MR

**Table 3:** Estimated causal effects between periodontitis and measurements of brain atrophy using different MR methods in replication cohort

**Exposure**	**Outcome**	**N.snps**	**Method**	**Beta**	**95% CI**	***P*-value**
Periodontitis	Cortical Surface Area	16	IVW	246.432	-301.562,794.426	0.378
			MR Egger	207.610	-1064.112,1479.332	0.754
			Weighted median	206.822	-591.402,1005.046	0.612
			MR-RAPS	240.559	-356.737,837.854	0.430
	Cortical Thickness	18	IVW	-0.002	-0.006,0.002	0.263
			MR Egger	0.003	-0.003,0.01	0.334
			Weighted median	-0.001	-0.006,0.004	0.746
			MR-RAPS	-0.002	-0.006,0.002	0.292
	Right Hippocampal volume	15	IVW	-6.191	-21.355,8.972	0.424
			MR Egger	-10.543	-53.575,32.489	0.639
			Weighted median	-4.765	-23.572,14.042	0.619
			MR-RAPS	-6.198	-20.737,8.342	0.403
	Left Hippocampal volume	15	IVW	-6.360	-19.149,6.43	0.330
			MR Egger	-14.486	-49.52,20.547	0.432
			Weighted median	-1.261	-18.844,16.321	0.888
			MR-RAPS	-5.997	-19.606,7.612	0.388

*IVW* Inverse variance weighted, *MR-RAPS* MR- robust adjusted profile score, *N.snps* Number of SNPs used in MR

Sensitivity analyses conducted for both forward and reverse MR in the two cohorts revealed no evidence of horizontal pleiotropy, as indicated by the MR Egger intercept test (*P* > 0.05) for the IVs, as detailed in Table S4. Additionally, the MR-PRESSO test identified no outliers in any of the analyses (Table S5). The Cochran's Q test suggested no heterogeneity (*P* > 0.05) among the SNPs, except for certain pairs (Exploration cohort: Left and Right Hippocampal volumes to Periodontitis; Replication cohort: Periodontitis to Cognitive performance and Reaction time) (Table S4). Although heterogeneity existed in the IVs of these four conditions, the results had less impact on causality as we chose a random effect IVW. Ultimately, the leave-one-out analyses demonstrated that the results were robust and not driven by any single SNP (Figure S7-S10).

## Discussion

Utilizing publicly accessible GWAS data, we employed a bidirectional Mendelian randomization approach to investigate, for the first time, the causal relationship between periodontitis and both brain atrophy and cognitive impairment. The results from both our exploration and replication cohorts consistently indicated the absence of a causal relationship between periodontitis and both brain atrophy and cognitive impairment. These findings remained consistent in the reverse analysis, reiterating the lack of a causal link in both directions. In addition, we conducted supplementary analyses to investigate the association between the longitudinal rates of change in brain atrophy and cognitive function with periodontitis. These results similarly did not demonstrate any significant relationships.

Up to the present, numerous studies have explored the link between periodontitis and both brain atrophy and cognitive impairment. However, the findings from these studies have presented inconsistent results. Regarding the nexus between periodontitis and cognitive impairment, one study based on the National Health and Nutrition Examination Survey (NHANES) III, linked periodontal status with cognitive impairment in US adults [38]. A longitudinal cohort study from the Swedish National Aging and Care Study (SNAC) pinpointed periodontitis (characterized by bone loss of ≥4 mm at more than 30% of discernible sites in baseline panoramic radiographs) as a standalone risk marker for cognitive degeneration over 6 years [39]. However, another relevant MR study indicated a lack of substantial evidence supporting periodontitis as a risk factor for the onset of AD [40]. Both of our research cohorts did not uncover any causal relationship between periodontitis and the four cognitive-related phenotypes or the four dementia-related phenotypes. Among the four cognitive-related phenotypes we employed, cognitive performance has been found to be associated with a variety of factors, such that individuals with Parkinson's disease, AD, and other dementia spectrum disorders typically exhibit significant neurocognitive deficits. Meanwhile, current research posits that cognitive performance in early life stages can predict long-term disease progression [41]. Fluid intelligence scores are primarily rooted in neurophysiology and closely linked to genetic factors [42]. Prospective memory and reaction time can effectively reflect the state of attention-related functions in the brain. These assessment indicators have proven to be reliable [15]. Within dementia-related phenotypes, Alzheimer's and vascular dementia rank as the top two in terms of dementia onset, while Lewy body dementia and frontotemporal dementia represent notable categories within the spectrum. Consequently, collectively analyzing these phenotypes is meaningful and provides a comprehensive understanding of the relationship between periodontitis and dementia. However, no significant results were obtained in the aforementioned studies. Although reverse analyses in the exploration cohort initially suggested a tentative connection between AD and periodontitis, this link lost significance after correction. Given the potential genetic heterogeneity between early-onset and late-onset AD, we conducted additional MR analyses to investigate the causal relationships between periodontitis and the two AD subtypes separately. However, the results remained non-significant. Our MR study aligns with previous investigations, [40] and the use of different GWAS data for periodontitis further enhances the robustness of our conclusions.

Regarding the association between periodontitis and brain atrophy, an investigation within the Atherosclerosis Risk in Communities (ARIC) study, focusing on an older participant cohort, revealed that periodontal disease exhibited no association with changes in brain volumes, microhemorrhages, or β-amyloid positivity [12]. Contrasting opinions and results persist, however, as exemplified by a study highlighting the interplay between the number of teeth and the periodontitis severity correlating with morphological alterations in the left hippocampus [43]. Our MR study utilized the surface area and thickness of the cerebral cortex, as well as the volume of the hippocampus, as assessment indicators for brain atrophy. The results of our study indicated that there is no causal relationship between periodontitis and the four brain atrophy assessment indicators in both research cohorts, and this finding remained consistent in the reverse analysis.

Regarding the reverse MR analyses in our study, the brain atrophy-related metrics and several cognitive function metrics were longitudinal measures that changed over time. However, the MR method treated these as single, static measurements, without considering the rates of change in these indicators over time. This could potentially impact the robustness of the results. Therefore, we introduced additional multiple GWAS that captured the longitudinal rates of change in brain atrophy and cognitive function, in order to conduct MR analyses using these as the exposures in relation to periodontitis. The results again did not show any significant associations. This supplementary analysis further corroborated the conclusions from the reverse MR, bolstering the overall rigor and validity of our study's findings.

Indeed, there is a connection between brain atrophy and cognitive impairment. Brain atrophy occurs years before symptoms of dementia appear with a stereotypical pattern of early medial temporal lobe (entorhinal cortex and hippocampus) involvement progressively extending to neocortical damage. Consistent with the gradual decline across multiple cognitive domains, atrophy in the hippocampus is associated with behavioral impairment as evaluated by the Mini-Mental State Examination (MMSE) and Auditory Verbal Learning Test (AVLT) [44]. In addition, both brain atrophy and vascular changes can lead to other types of cognitive impairments and dementia. Therefore, the results of studies exploring the link between periodontitis and brain atrophy (a notable characteristic of AD and other dementia forms), [45, 46] and those probing the association between periodontitis and dementia could be corroborated with each other somewhat. Our MR analysis considered both aspects and returned consistently negative findings, adding authenticity to the results.

Currently, observational studies entail numerous factors that jeopardize the validity of findings. Previous studies, for instance, had several inherent limitations. The design of studies based on NHANES III lacked a longitudinal assessment of periodontal status and did not include an objective assessment of oral hygiene and plaque [38]. The cognitive evaluation tool in the SNAC study, the Mini-Mental State Examination, carries intrinsic limitations largely dependent on verbal capabilities and has a restrained capacity to discern between cognitive impairment subtypes and dementia phenotypes [39]. The 5-year Japanese cohort study mentioned in the introduction section had a limited sample size (*n*=179) and an older cohort age (≥75 years) among other issues [7]. These observational studies, as well as some others in the field, additionally contained varying amounts of confounding elements, like certain outcome-related genes (apolipoprotein E gene), socio-economic factors, diet, lifestyle, and other potential outcome-associated confounders such as hypertension and hyperglycemia, highlighted in the limitations of the corresponding studies [7, 8, 39, 43, 47–49]. Hence, it is plausible that their regression methodologies might not depict an accurate correlation between exposure and outcome, especially with these known but unincorporated confounders [50]. Given these factors, the current observational studies' evidence reliability falls short of substantiating the formulation of clinical prevention or therapeutic approaches. In contrast, our MR study possesses numerous merits. Primarily, employing genetic variants as proxies for the assessed exposures can address reverse causality concerns: exposure-associated genetic variant alleles were allocated randomly, ensuring they were not prone to reverse causality [50]. The reverse MR test further validated the absence of a causal effect of the outcome on the exposures in the forward study. The chosen SNPs had to satisfy the three primary MR prerequisites, considerably reducing bias due to confounding factors [13]. Additionally, our MR study incorporated multiple sensitivity analysis methods. Instrumental variable (IV) heterogeneity and certain outliers stemming from the analysis received adjustments via suitable solutions, effectively sidestepping study result biases. Moreover, the sensitivity analysis also discounted potential pleiotropy levels. Our exposure and outcome databases predominantly originated from European population samples, effectively curtailing biases from population stratification. Consequently, our MR study stood as more reliable, furnishing a superior evidence tier. The findings revealed no causal association between periodontitis and either brain atrophy or cognitive impairment, and the reverse MR analysis echoed a similar lack of a causal link between brain atrophy, cognitive impairment and periodontitis. These insights will aid in clinical decision-making.

However, our MR study was not devoid of limitations. First, to select an appropriate number of periodontitis SNPs for IV usage, we opted for a *p*-value of 5 × 10^-6^ instead of 5 × 10^-8^, meaning that the SNPs used as IVs for periodontitis had a weaker association with the disease. Subsequently, our periodontitis GWAS data in the replication cohort employed ICD codes rather than clinical diagnoses, which could result in biased outcomes. Moreover, the GWAS data for brain atrophy and cognitive impairment employed in our study had a broad timeframe and did not stratify the analyses by age groups. Given the strong age-dependence of these indicators, this undifferentiated approach may have impacted the effect estimates to some degree, and should be considered a limitation of our study. This also constrains further analyses to elucidate whether periodontitis may impact the onset of cognitive impairment within specific timeframe (such as midlife, as suggested for other dementia risk factors) [51]. Finally, our study primarily relied on European population genetics databases, making the findings applicable only to European demographics.

## Conclusion

Our bidirectional MR analyses found no causal link between periodontitis and brain atrophy or cognitive impairment in either direction. The supplementary analyses also did not reveal any associations between the longitudinal rates of change in cognitive function and brain atrophy with periodontitis. Given the methodological strengths of our MR approachs and the comprehensive study design, we believe our results provide valuable insights into the potential causal relationships between periodontitis with brain atrophy and cognitive impairment. Future studies should design more rigorous randomized controlled trials to further investigate the relationships between periodontitis with brain atrophy and cognitive impairment, with the aim of resolving the longstanding controversies in this field and providing stronger evidence to guide clinical prevention and treatment strategies for the relevant diseases.

### Supplementary Information

Supplementary Material 1: Table S1. Additional information on all GWAS included in the study.

Supplementary Material 2: Table S2. Characterization of all SNPs included in the MR analysis.

Supplementary Material 3: Table S3. Information about all confounders-related SNPs.

Supplementary Material 4: Table S4. Cochran's Q test for heterogeneity and MR-Egger test for directional pleiotropy.

Supplementary Material 5: Table S5. MR-PRESSO test for heterogeneity and outlier exclusion.

Supplementary Material 6: Figure S1. Scatter plots of causal relationships of periodontitis with brain atrophy measures and cognitive impairment utilizing different MR methods (In exploration cohort).

Supplementary Material 7: Figure S2. Scatter plots of causal relationships of brain atrophy measures and cognitive impairment with periodontitis utilizing different MR methods (In exploration cohort).

Supplementary Material 8: Figure S3. Estimated causal effects of periodontitis with early-onset AD and late-onset AD using different MR methods in exploration cohort (forward and reverse direction).

Supplementary Material 9: Figure S4. Scatter plots and leave-one-out analysis of periodontitis with early-onset AD and late-onset AD in exploration cohort (forward and reverse direction).

Supplementary Material 10: Figure S5. Scatter plots of causal relationships of periodontitis with brain atrophy measures and cognitive impairment utilizing different MR methods (In replication cohort).

Supplementary Material 11: Figure S6. Scatter plots of causal relationships of brain atrophy measures and cognitive impairment with periodontitis utilizing different MR methods (In replication cohort).

Supplementary Material 12: Figure S7.docx Leave-one-out analysis of periodontitis with brain atrophy measures and cognitive impairment (In exploration cohort).

Supplementary Material 13: Figure S8. Leave-one-out analysis of brain atrophy measures and cognitive impairment with periodontitis (In exploration cohort).

Supplementary Material 14: Figure S9. Leave-one-out analysis of periodontitis with brain atrophy measures and cognitive impairment (In replication cohort).

Supplementary Material 15: Figure S10. Leave-one-out analysis of brain atrophy measures and cognitive impairment with periodontitis (In replication cohort).

## Data Availability

The datasets generated or analyzed during this study are included in supplementary material or in the data repositories listed in the methods.

## Abbreviations

GWAS: genome-wide association study
AD: Alzheimer's disease
MR: Mendelian randomization
IV: instrumental variable
MRI: magnetic resonance imaging
SNP: single nucleotide polymorphisms
FTP: frontotemporal dementia
IVW: inverse-variance weightedMR-RAPS: MR-robust adjusted profile score
OR: odds ratio
MR-PRESSO: MR-pleiotropy residual sum and outlierNHANES: National Health and Nutrition Examination Survey
SNAC: Swedish National Aging and Care
