# Supplementary material for: No genetic causal associations between periodontitis and brain atrophy or cognitive impairment: evidence from a comprehensive bidirectional Mendelian randomization study
Source: BMC Oral Health. 2024 May 16;24:571. doi: 10.1186/s12903-024-04367-7 (PMC11100120; doi:10.1186/s12903-024-04367-7)
Supplement: Supplementary file 1 — Supplementary Material 1: Table S1. Additional information on all GWAS included in the study. [file 12903_2024_4367_MOESM1_ESM.docx]

**Supplementary Table 1. Additional information on all GWAS included in the study.**

| **Traits** | **Consortium** | | **Adjustment** | **Supplementary Description** |
| --- | --- | --- | --- | --- |
| Periodontitis | GLIDE | | Age, age-squared and other study-specific covariates were instead included as covariates in association tests. | - |
|  | FinnGen | | The researchers used sex, age, genotyping batch and ten PCs as covariates | - |
|  |  | |  |  |
| Cerebral cortex | ENIGMA | | Within each cohort, adjustments were applied for age, age squared, sex, sex-age interaction, and the first four multidimensional scaling components.  Furthermore, diagnostic status and dummy variables pertinent to the MRI scanner in use were also considered. | Cortical metrics were extracted from 34 MRI scans of brain structures defined by the common Desikan-Killiany brain atlas, which coarsely partitions the cortex. |
|  |  | |  |  |
| Hippocampal volume | UK Biobank | | - | The research identified genetic associations between brain morphology, aging, and cognitive performance by modeling the overall dimensions of variation in the morphological structure of the human brain, based on a genomic principal component analysis approach. |
|  |  | |  |  |
| Cognitive performance | COGENT and UK Biobank | | A full set of dummy variables for year of birth, an indicator variable for sex, a full set of interactions between sex and year of birth, and the first 10 principal components of the variance-covariance matrix of the genetic relatedness matrix. | For each study under the COGENT meta-analysis, cognitive ability was assessed using the first unrotated principal component derived from a minimum of three neuropsychological test performances. Whereas in UK Biobank, standardized scores for verbal-numerical reasoning were generated from a test comprising thirteen questions with a two-minute time limit. |
|  |  | |  |  |
| Fluid intelligence score | UK Biobank | | - | Fluid intelligence scores were generated based on a simple unweighted sum of the number of correct answers participants gave to the thirteen fluid intelligence questions, a phenotype we label as a multidomain global measure of "fluid intelligence". |
|  |  | |  |  |
| Prospective memory | UK Biobank | | - | For the prospective memory assessment, participants were given up to two opportunities to recall the colorful images previously presented on the screen. The results were categorized ordinally, where a lower category indicated a superior performance on the test. |
|  |  | |  |  |
| Reaction time | CCACE | | Adjustments for age, sex, and population stratification were included in the model for each cohort. | The reaction time scores were calculated from a test of identifying the same cards, in which participants were given two cards to judge in 12 rounds of testing, and if they were the same, a button on a table would be pressed. The cumulative time taken to correctly identify matching cards was recorded as their reaction time. |
|  |  | |  |  |
| Alzheimer’s disease (AD) | - | | Adjusted for age, sex, the first four PCs (to correct for potential population stratification) and the number of APOE-ε4 and APOE- ε2 alleles (assuming an additive effect) | Patients with AD incorporated in this GWAS include those with a clinical diagnosis of AD or cases as proxy AD and related dementia (proxy-ADD). Proxy cases were based on questionnaire data where participants were asked if their parents had dementia. While this approach is less specific than clinical or pathologic diagnosis, it has been proven to be effective in prior studies. |
|  |  | |  |  |
| Early-onset AD | FinnGen | | The researchers used sex, age, genotyping batch and ten PCs as covariates | - |
|  |  | |  |  |
| Late-onset AD | FinnGen | | The researchers used sex, age, genotyping batch and ten PCs as covariates | - |
|  |  | |  |  |
| Vascular dementia | | UK Biobank | Age, sex and 10 PCs | - |
|  | |  |  |  |
| Lewy body dementia | | - | Sex, age, and five principal components (PC1, PC3, PC4, PC5, PC7) as covariates in our model | The study recruited 2,591 cases with Lewy body dementia and 4,027 neurologically healthy control samples, all of European ancestry. Diagnoses for patients were based on either pathological confirmation or clinical probability, in line with established consensus criteria. |
|  | |  |  |  |
| Frontotemporal dementia | | FinnGen | - | Diagnosis: ICD-10: F020&G310  Control excludes: F5_DEMENTIA, G6_ALZHEIMER, G6_AD_WIDE  (The specific definitions of these codes can be found in the Finngen project: https://www.finngen.fi/en) |
| Change rate in brain structure | | ENIGMA | - | - |
|  | |  |  |  |
|  | |  |  |  |
| Slope of cognitive decline | | - | Age, sex, years of education | In each cognitive domain, z-scores were created by first standardizing each test score according to the sample baseline mean and standard deviation, and then averaging the standardized test scores within each domain for participants with at least one non-missing test score in that domain. Global z-scores were created by averaging all of the standardized test scores for participants who were not missing more than one test score. |
|  | |  |  |  |
