# Supplementary material for: No genetic causal associations between periodontitis and brain atrophy or cognitive impairment: evidence from a comprehensive bidirectional Mendelian randomization study
Source: BMC Oral Health. 2024 May 16;24:571. doi: 10.1186/s12903-024-04367-7 (PMC11100120; doi:10.1186/s12903-024-04367-7)
Supplement: Supplementary file 2 — Supplementary Material 2: Table S2. Characterization of all SNPs included in the MR analysis. [file 12903_2024_4367_MOESM2_ESM.docx]

**Supplementary Table 2.** Characterization of all SNPs included in the MR analysis.

| **Exposure** | **Outcome** | **SNP** | **A1** | **A2** | **EAF** | **Exposure** | | | **R2** | **F** | **Outcome** | | |
| --- | --- | --- | --- | --- | --- | --- | --- | --- | --- | --- | --- | --- | --- |
|  |  |  |  |  |  | **beta** | **se** | **pval** |  |  | **beta** | **se** | **pval** |
| **Exploration Cohort (GLIDE)** | | | | | | | | | | | | | |
| Periodontitis | Cortical Surface Area | rs10143801 | A | G |  | -0.084 | 0.017 | 8.7E-07 | 5.3E-04 | 24.1 | 22.958 | 123.414 | 0.852 |
| Periodontitis | Cortical Surface Area | rs151226594 | T | G |  | -0.367 | 0.077 | 1.8E-06 | 6.7E-04 | 22.8 | 162.758 | 451.415 | 0.718 |
| Periodontitis | Cortical Surface Area | rs76734229 | A | G |  | -0.176 | 0.037 | 1.9E-06 | 5.0E-04 | 22.7 | 435.514 | 230.177 | 0.058 |
| Periodontitis | Cortical Surface Area | rs9954920 | T | C |  | 0.077 | 0.016 | 2.4E-06 | 4.9E-04 | 22.3 | 29.552 | 114.982 | 0.797 |
| Periodontitis | Cortical Thickness | rs10143801 | A | G |  | -0.084 | 0.017 | 8.7E-07 | 5.3E-04 | 24.1 | 0.000 | 0.001 | 0.726 |
| Periodontitis | Cortical Thickness | rs151226594 | T | G |  | -0.367 | 0.077 | 1.8E-06 | 6.7E-04 | 22.8 | 0.000 | 0.003 | 0.947 |
| Periodontitis | Cortical Thickness | rs76734229 | A | G |  | -0.176 | 0.037 | 1.9E-06 | 5.0E-04 | 22.7 | 0.003 | 0.002 | 0.051 |
| Periodontitis | Cortical Thickness | rs9954920 | T | C |  | 0.077 | 0.016 | 2.4E-06 | 4.9E-04 | 22.3 | 0.000 | 0.001 | 0.703 |
| Periodontitis | Right Hippocampal volume | rs10143801 | A | G |  | -0.084 | 0.017 | 8.7E-07 | 5.3E-04 | 24.1 | 1.425 | 3.055 | 0.641 |
| Periodontitis | Right Hippocampal volume | rs151226594 | T | G |  | -0.367 | 0.077 | 1.8E-06 | 6.7E-04 | 22.8 | 5.830 | 10.851 | 0.591 |
| Periodontitis | Right Hippocampal volume | rs73155039 | A | G |  | 0.832 | 0.176 | 2.2E-06 | 8.4E-04 | 22.4 | -3.993 | 11.351 | 0.725 |
| Periodontitis | Right Hippocampal volume | rs76734229 | A | G |  | -0.176 | 0.037 | 1.9E-06 | 5.0E-04 | 22.7 | -2.629 | 4.758 | 0.581 |
| Periodontitis | Right Hippocampal volume | rs9954920 | T | C |  | 0.077 | 0.016 | 2.4E-06 | 4.9E-04 | 22.3 | 0.039 | 2.827 | 0.989 |
| Periodontitis | Left Hippocampal volume | rs10143801 | A | G |  | -0.084 | 0.017 | 8.7E-07 | 5.3E-04 | 24.1 | 1.432 | 2.882 | 0.619 |
| Periodontitis | Left Hippocampal volume | rs151226594 | T | G |  | -0.367 | 0.077 | 1.8E-06 | 6.7E-04 | 22.8 | 13.088 | 10.230 | 0.201 |
| Periodontitis | Left Hippocampal volume | rs73155039 | A | G |  | 0.832 | 0.176 | 2.2E-06 | 8.4E-04 | 22.4 | -3.151 | 10.695 | 0.768 |
| Periodontitis | Left Hippocampal volume | rs76734229 | A | G |  | -0.176 | 0.037 | 1.9E-06 | 5.0E-04 | 22.7 | -3.538 | 4.489 | 0.431 |
| Periodontitis | Left Hippocampal volume | rs9954920 | T | C |  | 0.077 | 0.016 | 2.4E-06 | 4.9E-04 | 22.3 | -0.254 | 2.667 | 0.924 |
| Periodontitis | Cognitive Performance | rs10143801 | A | G |  | -0.084 | 0.017 | 8.7E-07 | 5.3E-04 | 24.1 | -0.003 | 0.003 | 0.407 |
| Periodontitis | Cognitive Performance | rs138868497 | T | C |  | 1.639 | 0.332 | 8.2E-07 | 6.8E-03 | 24.3 | -0.004 | 0.016 | 0.812 |
| Periodontitis | Cognitive Performance | rs151226594 | T | G |  | -0.367 | 0.077 | 1.8E-06 | 6.7E-04 | 22.8 | 0.006 | 0.011 | 0.598 |
| Periodontitis | Cognitive Performance | rs73155039 | A | G |  | 0.832 | 0.176 | 2.2E-06 | 8.4E-04 | 22.4 | -0.004 | 0.012 | 0.740 |
| Periodontitis | Cognitive Performance | rs76734229 | A | G |  | -0.176 | 0.037 | 1.9E-06 | 5.0E-04 | 22.7 | -0.004 | 0.005 | 0.431 |
| Periodontitis | Cognitive Performance | rs9954920 | T | C |  | 0.077 | 0.016 | 2.4E-06 | 4.9E-04 | 22.3 | 0.005 | 0.003 | 0.085 |
| Periodontitis | Fluid Intelligence Score | rs10143801 | A | G |  | -0.084 | 0.017 | 8.7E-07 | 5.3E-04 | 24.1 | 0.002 | 0.009 | 0.790 |
| Periodontitis | Fluid Intelligence Score | rs138868497 | T | C |  | 1.639 | 0.332 | 8.2E-07 | 6.8E-03 | 24.3 | -0.038 | 0.045 | 0.400 |
| Periodontitis | Fluid Intelligence Score | rs151226594 | T | G |  | -0.367 | 0.077 | 1.8E-06 | 6.7E-04 | 22.8 | 0.005 | 0.031 | 0.860 |
| Periodontitis | Fluid Intelligence Score | rs73155039 | A | G |  | 0.832 | 0.176 | 2.2E-06 | 8.4E-04 | 22.4 | -0.010 | 0.033 | 0.750 |
| Periodontitis | Fluid Intelligence Score | rs76734229 | A | G |  | -0.176 | 0.037 | 1.9E-06 | 5.0E-04 | 22.7 | -0.005 | 0.013 | 0.710 |
| Periodontitis | Fluid Intelligence Score | rs9954920 | T | C |  | 0.077 | 0.016 | 2.4E-06 | 4.9E-04 | 22.3 | 0.006 | 0.008 | 0.450 |
| Periodontitis | Prospective Memory | rs10143801 | A | G |  | -0.084 | 0.017 | 8.7E-07 | 5.3E-04 | 24.1 | 0.001 | 0.002 | 0.720 |
| Periodontitis | Prospective Memory | rs138868497 | T | C |  | 1.639 | 0.332 | 8.2E-07 | 6.8E-03 | 24.3 | -0.004 | 0.011 | 0.690 |
| Periodontitis | Prospective Memory | rs151226594 | T | G |  | -0.367 | 0.077 | 1.8E-06 | 6.7E-04 | 22.8 | -0.003 | 0.008 | 0.700 |
| Periodontitis | Prospective Memory | rs73155039 | A | G |  | 0.832 | 0.176 | 2.2E-06 | 8.4E-04 | 22.4 | -0.001 | 0.008 | 0.870 |
| Periodontitis | Prospective Memory | rs76734229 | A | G |  | -0.176 | 0.037 | 1.9E-06 | 5.0E-04 | 22.7 | -0.003 | 0.003 | 0.430 |
| Periodontitis | Prospective Memory | rs9954920 | T | C |  | 0.077 | 0.016 | 2.4E-06 | 4.9E-04 | 22.3 | -0.001 | 0.002 | 0.530 |
| Periodontitis | Reaction Time | rs10143801 | A | G |  | -0.084 | 0.017 | 8.7E-07 | 5.3E-04 | 24.1 | 0.001 | 0.002 | 0.620 |
| Periodontitis | Reaction Time | rs138868497 | T | C |  | 1.639 | 0.332 | 8.2E-07 | 6.8E-03 | 24.3 | 0.002 | 0.002 | 0.236 |
| Periodontitis | Reaction Time | rs151226594 | T | G |  | -0.367 | 0.077 | 1.8E-06 | 6.7E-04 | 22.8 | -0.002 | 0.002 | 0.249 |
| Periodontitis | Reaction Time | rs73155039 | A | G |  | 0.832 | 0.176 | 2.2E-06 | 8.4E-04 | 22.4 | 0.001 | 0.002 | 0.421 |
| Periodontitis | Reaction Time | rs76734229 | A | G |  | -0.176 | 0.037 | 1.9E-06 | 5.0E-04 | 22.7 | 0.002 | 0.002 | 0.262 |
| Periodontitis | Reaction Time | rs9954920 | T | C |  | 0.077 | 0.016 | 2.4E-06 | 4.9E-04 | 22.3 | -0.001 | 0.002 | 0.627 |
| Periodontitis | Alzheimer's disease | rs10143801 | A | G |  | -0.084 | 0.017 | 8.7E-07 | 5.3E-04 | 24.1 | -0.014 | 0.009 | 0.138 |
| Periodontitis | Alzheimer's disease | rs138868497 | T | C |  | 1.639 | 0.332 | 8.2E-07 | 6.8E-03 | 24.3 | 0.012 | 0.044 | 0.787 |
| Periodontitis | Alzheimer's disease | rs151226594 | T | G |  | -0.367 | 0.077 | 1.8E-06 | 6.7E-04 | 22.8 | 0.014 | 0.033 | 0.668 |
| Periodontitis | Alzheimer's disease | rs73155039 | A | G |  | 0.832 | 0.176 | 2.2E-06 | 8.4E-04 | 22.4 | -0.026 | 0.036 | 0.463 |
| Periodontitis | Alzheimer's disease | rs76734229 | A | G |  | -0.176 | 0.037 | 1.9E-06 | 5.0E-04 | 22.7 | -0.002 | 0.015 | 0.883 |
| Periodontitis | Alzheimer's disease | rs9954920 | T | C |  | 0.077 | 0.016 | 2.4E-06 | 4.9E-04 | 22.3 | 0.016 | 0.009 | 0.066 |
| Periodontitis | Early-onset AD | rs10143801 | A | G |  | -0.084 | 0.017 | 8.7E-07 | 5.3E-04 | 24.1 | -0.010 | 0.041 | 0.796 |
| Periodontitis | Early-onset AD | rs138868497 | T | C |  | 1.639 | 0.332 | 8.2E-07 | 6.8E-03 | 24.3 | -0.143 | 0.178 | 0.420 |
| Periodontitis | Early-onset AD | rs151226594 | T | G |  | -0.367 | 0.077 | 1.8E-06 | 6.7E-04 | 22.8 | -0.041 | 0.137 | 0.764 |
| Periodontitis | Early-onset AD | rs73155039 | A | G |  | 0.832 | 0.176 | 2.2E-06 | 8.4E-04 | 22.4 | -0.108 | 0.244 | 0.656 |
| Periodontitis | Early-onset AD | rs76734229 | A | G |  | -0.176 | 0.037 | 1.9E-06 | 5.0E-04 | 22.7 | -0.068 | 0.068 | 0.319 |
| Periodontitis | Early-onset AD | rs9954920 | T | C |  | 0.077 | 0.016 | 2.4E-06 | 4.9E-04 | 22.3 | 0.027 | 0.041 | 0.520 |
| Periodontitis | Late-onset AD | rs10143801 | A | G |  | -0.084 | 0.017 | 8.7E-07 | 5.3E-04 | 24.1 | 0.003 | 0.021 | 0.903 |
| Periodontitis | Late-onset AD | rs138868497 | T | C |  | 1.639 | 0.332 | 8.2E-07 | 6.8E-03 | 24.3 | -0.080 | 0.087 | 0.356 |
| Periodontitis | Late-onset AD | rs151226594 | T | G |  | -0.367 | 0.077 | 1.8E-06 | 6.7E-04 | 22.8 | 0.065 | 0.069 | 0.348 |
| Periodontitis | Late-onset AD | rs73155039 | A | G |  | 0.832 | 0.176 | 2.2E-06 | 8.4E-04 | 22.4 | -0.080 | 0.126 | 0.524 |
| Periodontitis | Late-onset AD | rs76734229 | A | G |  | -0.176 | 0.037 | 1.9E-06 | 5.0E-04 | 22.7 | -0.055 | 0.034 | 0.104 |
| Periodontitis | Late-onset AD | rs9954920 | T | C |  | 0.077 | 0.016 | 2.4E-06 | 4.9E-04 | 22.3 | -0.011 | 0.021 | 0.606 |
| Periodontitis | Lewy body dementia | rs10143801 | A | G |  | -0.084 | 0.017 | 8.7E-07 | 5.3E-04 | 24.1 | -0.013 | 0.042 | 0.749 |
| Periodontitis | Lewy body dementia | rs138868497 | T | C |  | 1.639 | 0.332 | 8.2E-07 | 6.8E-03 | 24.3 | -0.209 | 0.196 | 0.285 |
| Periodontitis | Lewy body dementia | rs151226594 | T | G |  | -0.367 | 0.077 | 1.8E-06 | 6.7E-04 | 22.8 | 0.059 | 0.142 | 0.680 |
| Periodontitis | Lewy body dementia | rs73155039 | A | G |  | 0.832 | 0.176 | 2.2E-06 | 8.4E-04 | 22.4 | -0.060 | 0.154 | 0.695 |
| Periodontitis | Lewy body dementia | rs76734229 | A | G |  | -0.176 | 0.037 | 1.9E-06 | 5.0E-04 | 22.7 | -0.055 | 0.068 | 0.415 |
| Periodontitis | Lewy body dementia | rs9954920 | T | C |  | 0.077 | 0.016 | 2.4E-06 | 4.9E-04 | 22.3 | 0.014 | 0.038 | 0.718 |
| Periodontitis | Vascular Dementia | rs10143801 | A | G |  | -0.084 | 0.017 | 8.7E-07 | 5.3E-04 | 24.1 | -0.059 | 0.076 | 0.439 |
| Periodontitis | Vascular Dementia | rs138868497 | T | C |  | 1.639 | 0.332 | 8.2E-07 | 6.8E-03 | 24.3 | 0.417 | 0.396 | 0.293 |
| Periodontitis | Vascular Dementia | rs151226594 | T | G |  | -0.367 | 0.077 | 1.8E-06 | 6.7E-04 | 22.8 | -0.185 | 0.271 | 0.494 |
| Periodontitis | Vascular Dementia | rs73155039 | A | G |  | 0.832 | 0.176 | 2.2E-06 | 8.4E-04 | 22.4 | -0.273 | 0.289 | 0.345 |
| Periodontitis | Vascular Dementia | rs76734229 | A | G |  | -0.176 | 0.037 | 1.9E-06 | 5.0E-04 | 22.7 | 0.099 | 0.117 | 0.398 |
| Periodontitis | Vascular Dementia | rs9954920 | T | C |  | 0.077 | 0.016 | 2.4E-06 | 4.9E-04 | 22.3 | 0.134 | 0.070 | 0.055 |
| Periodontitis | Frontotemporal Dementia | rs10143801 | A | G |  | -0.084 | 0.017 | 8.7E-07 | 5.3E-04 | 24.1 | 0.045 | 0.130 | 0.730 |
| Periodontitis | Frontotemporal Dementia | rs138868497 | T | C |  | 1.639 | 0.332 | 8.2E-07 | 6.8E-03 | 24.3 | -0.991 | 0.570 | 0.082 |
| Periodontitis | Frontotemporal Dementia | rs151226594 | T | G |  | -0.367 | 0.077 | 1.8E-06 | 6.7E-04 | 22.8 | -0.270 | 0.431 | 0.531 |
| Periodontitis | Frontotemporal Dementia | rs73155039 | A | G |  | 0.832 | 0.176 | 2.2E-06 | 8.4E-04 | 22.4 | 1.060 | 0.858 | 0.216 |
| Periodontitis | Frontotemporal Dementia | rs76734229 | A | G |  | -0.176 | 0.037 | 1.9E-06 | 5.0E-04 | 22.7 | -0.113 | 0.222 | 0.610 |
| Periodontitis | Frontotemporal Dementia | rs9954920 | T | C |  | 0.077 | 0.016 | 2.4E-06 | 4.9E-04 | 22.3 | -0.265 | 0.134 | 0.048 |
|  |  |  |  |  |  |  |  |  |  |  |  |  |  |
|  |  |  |  |  |  |  |  |  |  |  |  |  |  |
| Cortical Surface Area | Periodontitis | rs10878349 | A | G | 0.483 | 1040.514 | 111.325 | 9.0E-21 | 2.6E-03 | 87.4 | 0.019 | 0.015 | 0.212 |
| Cortical Surface Area | Periodontitis | rs1628768 | T | C | 0.764 | -918.535 | 133.630 | 6.3E-12 | 1.4E-03 | 47.2 | 0.017 | 0.020 | 0.383 |
| Cortical Surface Area | Periodontitis | rs2301718 | A | G | 0.240 | 729.921 | 132.345 | 3.5E-08 | 9.1E-04 | 30.4 | 0.020 | 0.018 | 0.272 |
| Cortical Surface Area | Periodontitis | rs62057153 | T | C | 0.780 | 1685.224 | 145.149 | 3.7E-31 | 4.4E-03 | 134.8 | -0.014 | 0.025 | 0.572 |
| Cortical Surface Area | Periodontitis | rs7715167 | T | C | 0.386 | -690.511 | 120.803 | 1.1E-08 | 9.9E-04 | 32.7 | 0.003 | 0.016 | 0.849 |
| Cortical Surface Area | Periodontitis | rs9832327 | T | C | 0.749 | -720.991 | 127.737 | 1.7E-08 | 9.5E-04 | 31.9 | -0.002 | 0.019 | 0.930 |
| Cortical Thickness | Periodontitis | rs11612673 | T | C | 0.532 | 0.004 | 0.001 | 1.9E-08 | 8.2E-04 | 28.9 | -0.016 | 0.017 | 0.348 |
| Cortical Thickness | Periodontitis | rs11692435 | A | G | 0.090 | -0.009 | 0.002 | 1.3E-09 | 1.2E-03 | 36.0 | -0.006 | 0.030 | 0.844 |
| Cortical Thickness | Periodontitis | rs13110077 | T | C | 0.235 | 0.005 | 0.001 | 1.9E-08 | 8.4E-04 | 29.6 | 0.003 | 0.019 | 0.855 |
| Cortical Thickness | Periodontitis | rs2316767 | T | C | 0.791 | -0.007 | 0.001 | 7.3E-10 | 1.4E-03 | 37.1 | 0.000 | 0.030 | 0.998 |
| Cortical Thickness | Periodontitis | rs3816046 | T | C | 0.321 | -0.004 | 0.001 | 2.0E-08 | 9.3E-04 | 30.3 | -0.011 | 0.017 | 0.515 |
| Cortical Thickness | Periodontitis | rs630934 | A | C | 0.496 | -0.005 | 0.001 | 1.2E-12 | 1.3E-03 | 45.6 | 0.021 | 0.015 | 0.162 |
| Cortical Thickness | Periodontitis | rs7824177 | A | G | 0.823 | 0.005 | 0.001 | 3.6E-08 | 9.5E-04 | 33.4 | 0.031 | 0.020 | 0.122 |
| Right Hippocampal volume | Periodontitis | rs11206344 | C | A | 0.501 | 17.917 | 2.710 | 3.8E-11 | 1.2E-03 | 43.7 | 0.020 | 0.016 | 0.199 |
| Right Hippocampal volume | Periodontitis | rs1423642 | A | C | 0.653 | -17.337 | 2.855 | 1.3E-09 | 1.0E-03 | 36.9 | -0.009 | 0.017 | 0.576 |
| Right Hippocampal volume | Periodontitis | rs146607495 | C | T | 0.903 | -62.616 | 4.655 | 3.0E-41 | 4.9E-03 | 180.9 | 0.037 | 0.027 | 0.171 |
| Right Hippocampal volume | Periodontitis | rs17178006 | T | G | 0.883 | 54.969 | 4.229 | 1.3E-38 | 4.6E-03 | 168.9 | -0.070 | 0.031 | 0.021 |
| Right Hippocampal volume | Periodontitis | rs1741948 | T | G | 0.389 | -15.246 | 2.778 | 4.1E-08 | 8.2E-04 | 30.1 | 0.003 | 0.016 | 0.826 |
| Right Hippocampal volume | Periodontitis | rs2578475 | T | G | 0.657 | 19.904 | 2.886 | 5.3E-12 | 1.3E-03 | 47.6 | 0.040 | 0.018 | 0.029 |
| Right Hippocampal volume | Periodontitis | rs28758826 | A | G | 0.451 | 16.080 | 2.744 | 4.6E-09 | 9.3E-04 | 34.4 | 0.019 | 0.016 | 0.230 |
| Right Hippocampal volume | Periodontitis | rs2970931 | T | C | 0.428 | 22.435 | 2.778 | 6.6E-16 | 1.8E-03 | 65.2 | -0.005 | 0.016 | 0.761 |
| Right Hippocampal volume | Periodontitis | rs309587 | C | T | 0.317 | 21.238 | 2.915 | 3.2E-13 | 1.4E-03 | 53.1 | 0.012 | 0.017 | 0.493 |
| Right Hippocampal volume | Periodontitis | rs33931638 | G | A | 0.926 | 30.496 | 5.185 | 4.1E-09 | 9.4E-04 | 34.6 | -0.081 | 0.030 | 0.008 |
| Right Hippocampal volume | Periodontitis | rs55905347 | G | A | 0.640 | 17.174 | 2.883 | 2.6E-09 | 9.6E-04 | 35.5 | -0.004 | 0.017 | 0.795 |
| Right Hippocampal volume | Periodontitis | rs929387 | G | A | 0.683 | -16.545 | 2.939 | 1.8E-08 | 8.6E-04 | 31.7 | 0.037 | 0.016 | 0.026 |
| Right Hippocampal volume | Periodontitis | rs9322190 | G | A | 0.670 | -22.607 | 2.885 | 4.6E-15 | 1.7E-03 | 61.4 | -0.018 | 0.017 | 0.291 |
| Left Hippocampal volume | Periodontitis | rs11206344 | C | A | 0.501 | 18.751 | 2.557 | 2.3E-13 | 1.5E-03 | 53.8 | 0.020 | 0.016 | 0.199 |
| Left Hippocampal volume | Periodontitis | rs146607495 | C | T | 0.903 | -60.031 | 4.391 | 1.5E-42 | 5.1E-03 | 186.9 | 0.037 | 0.027 | 0.171 |
| Left Hippocampal volume | Periodontitis | rs17178006 | T | G | 0.883 | 49.303 | 3.990 | 4.5E-35 | 4.1E-03 | 152.7 | -0.070 | 0.031 | 0.021 |
| Left Hippocampal volume | Periodontitis | rs17205972 | G | T | 0.806 | -18.711 | 3.229 | 6.9E-09 | 9.1E-04 | 33.6 | 0.000 | 0.020 | 0.983 |
| Left Hippocampal volume | Periodontitis | rs2287509 | G | T | 0.426 | 17.937 | 2.622 | 7.8E-12 | 1.3E-03 | 46.8 | -0.001 | 0.016 | 0.939 |
| Left Hippocampal volume | Periodontitis | rs2578475 | T | G | 0.657 | 20.297 | 2.723 | 9.0E-14 | 1.5E-03 | 55.6 | 0.040 | 0.018 | 0.029 |
| Left Hippocampal volume | Periodontitis | rs28740897 | G | A | 0.660 | -20.462 | 2.738 | 7.8E-14 | 1.5E-03 | 55.9 | 0.094 | 0.145 | 0.517 |
| Left Hippocampal volume | Periodontitis | rs28758826 | A | G | 0.451 | 14.714 | 2.588 | 1.3E-08 | 8.8E-04 | 32.3 | 0.019 | 0.016 | 0.230 |
| Left Hippocampal volume | Periodontitis | rs35620312 | A | G | 0.772 | -17.678 | 3.100 | 1.2E-08 | 8.8E-04 | 32.5 | -0.001 | 0.021 | 0.949 |
| Left Hippocampal volume | Periodontitis | rs58490349 | T | C | 0.980 | -50.471 | 9.235 | 4.6E-08 | 8.1E-04 | 29.9 | -0.126 | 0.060 | 0.034 |
| Left Hippocampal volume | Periodontitis | rs61785580 | C | T | 0.928 | 30.292 | 5.028 | 1.7E-09 | 9.9E-04 | 36.3 | -0.069 | 0.032 | 0.028 |
| Left Hippocampal volume | Periodontitis | rs72761270 | C | T | 0.648 | -16.966 | 2.695 | 3.0E-10 | 1.1E-03 | 39.6 | -0.007 | 0.017 | 0.683 |
| Cognitive Performance | Periodontitis | rs1009950 | T | G | 0.418 | -0.019 | 0.003 | 8.2E-11 | 1.8E-04 | 45.5 | 0.046 | 0.020 | 0.020 |
| Cognitive Performance | Periodontitis | rs10129426 | G | A | 0.469 | -0.019 | 0.003 | 1.9E-11 | 1.8E-04 | 47.7 | 0.014 | 0.015 | 0.352 |
| Cognitive Performance | Periodontitis | rs10865397 | G | A | 0.522 | 0.017 | 0.003 | 6.8E-09 | 1.4E-04 | 36.1 | -0.004 | 0.017 | 0.821 |
| Cognitive Performance | Periodontitis | rs10875914 | G | A | 0.355 | 0.024 | 0.003 | 3.5E-16 | 2.6E-04 | 66.5 | -0.012 | 0.016 | 0.448 |
| Cognitive Performance | Periodontitis | rs10990610 | T | C | 0.835 | -0.025 | 0.004 | 8.7E-11 | 1.7E-04 | 44.3 | -0.015 | 0.022 | 0.498 |
| Cognitive Performance | Periodontitis | rs11138947 | C | T | 0.267 | -0.017 | 0.003 | 4.8E-08 | 1.2E-04 | 30.5 | 0.012 | 0.017 | 0.483 |
| Cognitive Performance | Periodontitis | rs112780312 | G | A | 0.747 | 0.021 | 0.003 | 3.7E-11 | 1.7E-04 | 43.9 | -0.016 | 0.017 | 0.366 |
| Cognitive Performance | Periodontitis | rs1144593 | G | A | 0.299 | 0.023 | 0.003 | 1.8E-13 | 2.2E-04 | 56.0 | 0.007 | 0.017 | 0.696 |
| Cognitive Performance | Periodontitis | rs11662271 | T | C | 0.485 | 0.023 | 0.003 | 4.4E-16 | 2.7E-04 | 69.3 | -0.007 | 0.016 | 0.641 |
| Cognitive Performance | Periodontitis | rs11720523 | C | A | 0.554 | -0.016 | 0.003 | 1.7E-08 | 1.3E-04 | 33.5 | -0.026 | 0.016 | 0.103 |
| Cognitive Performance | Periodontitis | rs12435486 | A | G | 0.248 | -0.018 | 0.003 | 3.8E-08 | 1.2E-04 | 32.0 | 0.019 | 0.018 | 0.292 |
| Cognitive Performance | Periodontitis | rs12439619 | G | T | 0.313 | 0.020 | 0.003 | 7.9E-11 | 1.7E-04 | 44.1 | 0.010 | 0.017 | 0.563 |
| Cognitive Performance | Periodontitis | rs12635303 | C | T | 0.708 | -0.018 | 0.003 | 2.2E-09 | 1.4E-04 | 36.5 | -0.026 | 0.017 | 0.117 |
| Cognitive Performance | Periodontitis | rs13253386 | T | G | 0.495 | -0.018 | 0.003 | 1.3E-10 | 1.7E-04 | 43.5 | -0.002 | 0.016 | 0.887 |
| Cognitive Performance | Periodontitis | rs13428598 | T | C | 0.379 | 0.020 | 0.003 | 1.3E-11 | 1.8E-04 | 47.5 | 0.000 | 0.016 | 0.992 |
| Cognitive Performance | Periodontitis | rs136554 | A | G | 0.459 | 0.016 | 0.003 | 3.5E-08 | 1.2E-04 | 32.2 | 0.017 | 0.016 | 0.279 |
| Cognitive Performance | Periodontitis | rs1391438 | T | C | 0.315 | 0.017 | 0.003 | 3.3E-08 | 1.2E-04 | 31.8 | 0.007 | 0.016 | 0.665 |
| Cognitive Performance | Periodontitis | rs1408579 | C | T | 0.500 | -0.017 | 0.003 | 4.1E-09 | 1.4E-04 | 36.4 | 0.007 | 0.016 | 0.667 |
| Cognitive Performance | Periodontitis | rs1415802 | T | G | 0.597 | -0.017 | 0.003 | 4.8E-09 | 1.4E-04 | 36.6 | 0.004 | 0.016 | 0.811 |
| Cognitive Performance | Periodontitis | rs1479073 | C | T | 0.311 | 0.017 | 0.003 | 1.9E-08 | 1.3E-04 | 33.1 | 0.006 | 0.017 | 0.726 |
| Cognitive Performance | Periodontitis | rs1507010 | G | A | 0.495 | 0.017 | 0.003 | 1.5E-09 | 1.5E-04 | 38.5 | -0.005 | 0.016 | 0.773 |
| Cognitive Performance | Periodontitis | rs1523048 | T | C | 0.400 | 0.018 | 0.003 | 2.1E-09 | 1.5E-04 | 39.0 | 0.014 | 0.016 | 0.372 |
| Cognitive Performance | Periodontitis | rs1567154 | C | T | 0.735 | 0.020 | 0.003 | 4.5E-09 | 1.6E-04 | 42.0 | 0.005 | 0.020 | 0.812 |
| Cognitive Performance | Periodontitis | rs159428 | T | C | 0.493 | 0.017 | 0.003 | 7.0E-09 | 1.4E-04 | 36.2 | -0.022 | 0.017 | 0.201 |
| Cognitive Performance | Periodontitis | rs17002025 | A | G | 0.138 | 0.029 | 0.004 | 3.0E-11 | 2.0E-04 | 51.0 | 0.042 | 0.032 | 0.186 |
| Cognitive Performance | Periodontitis | rs17106817 | T | C | 0.716 | 0.019 | 0.003 | 6.9E-09 | 1.4E-04 | 36.0 | 0.018 | 0.017 | 0.307 |
| Cognitive Performance | Periodontitis | rs17428810 | T | C | 0.731 | 0.018 | 0.003 | 8.1E-09 | 1.3E-04 | 32.4 | -0.030 | 0.017 | 0.076 |
| Cognitive Performance | Periodontitis | rs1892419 | C | T | 0.233 | -0.028 | 0.003 | 3.5E-16 | 2.7E-04 | 70.2 | 0.005 | 0.019 | 0.787 |
| Cognitive Performance | Periodontitis | rs2005078 | G | A | 0.333 | 0.019 | 0.003 | 2.4E-10 | 1.6E-04 | 42.4 | 0.007 | 0.016 | 0.663 |
| Cognitive Performance | Periodontitis | rs2143103 | A | G | 0.145 | 0.024 | 0.004 | 9.6E-09 | 1.4E-04 | 36.2 | 0.012 | 0.025 | 0.622 |
| Cognitive Performance | Periodontitis | rs2180111 | A | G | 0.294 | 0.018 | 0.003 | 1.6E-08 | 1.4E-04 | 35.2 | 0.005 | 0.017 | 0.769 |
| Cognitive Performance | Periodontitis | rs2439649 | G | A | 0.461 | -0.016 | 0.003 | 2.0E-08 | 1.3E-04 | 33.0 | 0.023 | 0.015 | 0.134 |
| Cognitive Performance | Periodontitis | rs2478281 | G | A | 0.247 | 0.023 | 0.003 | 3.1E-12 | 1.9E-04 | 49.5 | -0.021 | 0.018 | 0.229 |
| Cognitive Performance | Periodontitis | rs26046 | T | C | 0.367 | -0.020 | 0.003 | 2.4E-12 | 1.9E-04 | 50.1 | -0.038 | 0.016 | 0.019 |
| Cognitive Performance | Periodontitis | rs2647995 | C | T | 0.293 | 0.019 | 0.003 | 5.0E-09 | 1.4E-04 | 37.0 | 0.026 | 0.017 | 0.133 |
| Cognitive Performance | Periodontitis | rs2652454 | T | C | 0.478 | -0.016 | 0.003 | 3.2E-08 | 1.3E-04 | 32.4 | 0.019 | 0.015 | 0.224 |
| Cognitive Performance | Periodontitis | rs2721173 | C | T | 0.551 | 0.017 | 0.003 | 6.1E-09 | 1.4E-04 | 35.2 | -0.012 | 0.015 | 0.426 |
| Cognitive Performance | Periodontitis | rs2737339 | G | A | 0.410 | 0.018 | 0.003 | 4.1E-10 | 1.6E-04 | 41.6 | 0.005 | 0.016 | 0.747 |
| Cognitive Performance | Periodontitis | rs276626 | G | A | 0.162 | -0.021 | 0.004 | 4.8E-08 | 1.2E-04 | 30.1 | 0.027 | 0.020 | 0.167 |
| Cognitive Performance | Periodontitis | rs2799399 | G | T | 0.583 | 0.016 | 0.003 | 4.6E-08 | 1.2E-04 | 31.0 | 0.008 | 0.016 | 0.598 |
| Cognitive Performance | Periodontitis | rs2806048 | A | G | 0.643 | -0.019 | 0.003 | 1.3E-10 | 1.7E-04 | 42.6 | -0.034 | 0.017 | 0.043 |
| Cognitive Performance | Periodontitis | rs2852931 | A | G | 0.849 | 0.028 | 0.004 | 1.1E-10 | 2.1E-04 | 53.5 | 0.003 | 0.025 | 0.908 |
| Cognitive Performance | Periodontitis | rs2977464 | T | C | 0.168 | 0.021 | 0.004 | 9.9E-09 | 1.2E-04 | 31.6 | 0.012 | 0.019 | 0.523 |
| Cognitive Performance | Periodontitis | rs3128341 | T | C | 0.175 | -0.033 | 0.004 | 2.5E-21 | 3.2E-04 | 83.3 | 0.010 | 0.019 | 0.599 |
| Cognitive Performance | Periodontitis | rs335426 | C | A | 0.500 | -0.020 | 0.003 | 7.2E-12 | 1.9E-04 | 50.2 | 0.018 | 0.019 | 0.340 |
| Cognitive Performance | Periodontitis | rs34802460 | C | T | 0.743 | -0.019 | 0.004 | 4.9E-08 | 1.4E-04 | 36.2 | -0.003 | 0.025 | 0.919 |
| Cognitive Performance | Periodontitis | rs3860537 | T | C | 0.242 | 0.019 | 0.003 | 3.1E-08 | 1.3E-04 | 32.7 | -0.024 | 0.020 | 0.217 |
| Cognitive Performance | Periodontitis | rs39302 | C | T | 0.808 | 0.021 | 0.004 | 4.5E-09 | 1.4E-04 | 34.9 | -0.035 | 0.020 | 0.080 |
| Cognitive Performance | Periodontitis | rs3943667 | T | C | 0.723 | -0.019 | 0.003 | 3.6E-09 | 1.4E-04 | 35.4 | -0.018 | 0.017 | 0.292 |
| Cognitive Performance | Periodontitis | rs4342312 | C | A | 0.626 | 0.018 | 0.003 | 1.1E-09 | 1.5E-04 | 38.8 | -0.008 | 0.016 | 0.590 |
| Cognitive Performance | Periodontitis | rs4347883 | T | C | 0.561 | -0.016 | 0.003 | 2.8E-08 | 1.3E-04 | 32.3 | 0.007 | 0.016 | 0.654 |
| Cognitive Performance | Periodontitis | rs4463213 | A | G | 0.541 | 0.022 | 0.003 | 2.6E-14 | 2.4E-04 | 61.0 | 0.003 | 0.015 | 0.846 |
| Cognitive Performance | Periodontitis | rs4937860 | G | A | 0.094 | -0.026 | 0.005 | 2.4E-08 | 1.1E-04 | 29.3 | -0.011 | 0.026 | 0.664 |
| Cognitive Performance | Periodontitis | rs4976976 | A | G | 0.386 | 0.020 | 0.003 | 1.2E-11 | 1.9E-04 | 47.8 | -0.022 | 0.016 | 0.165 |
| Cognitive Performance | Periodontitis | rs56135595 | T | G | 0.141 | -0.022 | 0.004 | 3.0E-08 | 1.2E-04 | 30.7 | -0.009 | 0.022 | 0.688 |
| Cognitive Performance | Periodontitis | rs5751191 | C | T | 0.478 | -0.022 | 0.003 | 8.9E-15 | 2.4E-04 | 63.1 | 0.004 | 0.016 | 0.784 |
| Cognitive Performance | Periodontitis | rs58489175 | G | A | 0.282 | 0.021 | 0.003 | 2.0E-11 | 1.8E-04 | 47.0 | 0.017 | 0.018 | 0.327 |
| Cognitive Performance | Periodontitis | rs602512 | G | A | 0.614 | -0.019 | 0.003 | 2.9E-11 | 1.8E-04 | 46.4 | 0.008 | 0.016 | 0.617 |
| Cognitive Performance | Periodontitis | rs61815057 | A | G | 0.396 | 0.017 | 0.003 | 8.1E-09 | 1.4E-04 | 34.9 | 0.027 | 0.016 | 0.088 |
| Cognitive Performance | Periodontitis | rs62047970 | T | G | 0.578 | -0.019 | 0.003 | 9.9E-11 | 1.7E-04 | 44.4 | -0.004 | 0.018 | 0.817 |
| Cognitive Performance | Periodontitis | rs620729 | A | C | 0.685 | -0.019 | 0.003 | 3.1E-10 | 1.6E-04 | 40.7 | -0.015 | 0.017 | 0.352 |
| Cognitive Performance | Periodontitis | rs62169190 | T | C | 0.177 | -0.020 | 0.004 | 2.7E-08 | 1.2E-04 | 31.3 | -0.010 | 0.022 | 0.657 |
| Cognitive Performance | Periodontitis | rs6535809 | G | A | 0.500 | -0.020 | 0.003 | 6.9E-12 | 1.9E-04 | 49.3 | -0.006 | 0.016 | 0.723 |
| Cognitive Performance | Periodontitis | rs6550835 | G | A | 0.697 | 0.025 | 0.003 | 3.9E-16 | 2.6E-04 | 66.9 | -0.012 | 0.016 | 0.464 |
| Cognitive Performance | Periodontitis | rs6587843 | T | C | 0.471 | 0.018 | 0.003 | 4.8E-10 | 1.6E-04 | 40.4 | -0.017 | 0.016 | 0.281 |
| Cognitive Performance | Periodontitis | rs66752974 | C | A | 0.842 | 0.022 | 0.004 | 7.9E-09 | 1.3E-04 | 33.7 | 0.001 | 0.021 | 0.951 |
| Cognitive Performance | Periodontitis | rs6819372 | G | A | 0.536 | 0.019 | 0.003 | 6.6E-11 | 1.7E-04 | 44.6 | -0.017 | 0.015 | 0.279 |
| Cognitive Performance | Periodontitis | rs6903716 | G | A | 0.274 | -0.018 | 0.003 | 4.6E-09 | 1.3E-04 | 34.8 | 0.013 | 0.017 | 0.434 |
| Cognitive Performance | Periodontitis | rs6952104 | T | C | 0.522 | 0.019 | 0.003 | 6.3E-11 | 1.7E-04 | 44.7 | -0.014 | 0.015 | 0.357 |
| Cognitive Performance | Periodontitis | rs6975134 | C | T | 0.422 | 0.022 | 0.003 | 4.6E-14 | 2.3E-04 | 60.6 | -0.016 | 0.016 | 0.327 |
| Cognitive Performance | Periodontitis | rs702222 | C | T | 0.672 | 0.020 | 0.003 | 1.7E-11 | 1.8E-04 | 45.6 | -0.014 | 0.016 | 0.387 |
| Cognitive Performance | Periodontitis | rs7044246 | T | C | 0.696 | 0.017 | 0.003 | 2.0E-08 | 1.3E-04 | 33.4 | -0.003 | 0.017 | 0.844 |
| Cognitive Performance | Periodontitis | rs7256776 | G | A | 0.709 | 0.019 | 0.003 | 9.3E-10 | 1.4E-04 | 37.0 | 0.026 | 0.017 | 0.115 |
| Cognitive Performance | Periodontitis | rs72739469 | T | C | 0.946 | -0.036 | 0.006 | 7.5E-09 | 1.3E-04 | 34.0 | -0.071 | 0.045 | 0.111 |
| Cognitive Performance | Periodontitis | rs72821233 | T | G | 0.272 | -0.020 | 0.003 | 4.9E-10 | 1.6E-04 | 41.3 | -0.012 | 0.018 | 0.505 |
| Cognitive Performance | Periodontitis | rs7312770 | T | C | 0.514 | -0.016 | 0.003 | 2.5E-08 | 1.3E-04 | 33.0 | 0.014 | 0.016 | 0.392 |
| Cognitive Performance | Periodontitis | rs73845427 | A | G | 0.029 | -0.047 | 0.008 | 7.7E-09 | 1.2E-04 | 31.4 | 0.050 | 0.048 | 0.294 |
| Cognitive Performance | Periodontitis | rs73989053 | A | G | 0.162 | -0.022 | 0.004 | 6.3E-09 | 1.3E-04 | 34.2 | 0.029 | 0.022 | 0.179 |
| Cognitive Performance | Periodontitis | rs74370218 | T | C | 0.383 | -0.018 | 0.003 | 9.8E-10 | 1.5E-04 | 39.5 | 0.008 | 0.016 | 0.601 |
| Cognitive Performance | Periodontitis | rs75973558 | G | A | 0.119 | -0.025 | 0.004 | 7.4E-09 | 1.3E-04 | 32.8 | -0.004 | 0.026 | 0.863 |
| Cognitive Performance | Periodontitis | rs7599860 | A | C | 0.228 | 0.020 | 0.003 | 3.9E-09 | 1.4E-04 | 35.1 | 0.030 | 0.020 | 0.138 |
| Cognitive Performance | Periodontitis | rs77128898 | T | C | 0.022 | -0.046 | 0.008 | 1.7E-09 | 9.3E-05 | 23.9 | 0.093 | 0.069 | 0.176 |
| Cognitive Performance | Periodontitis | rs78358737 | T | G | 0.966 | -0.042 | 0.007 | 6.2E-10 | 1.2E-04 | 30.6 | 0.026 | 0.041 | 0.529 |
| Cognitive Performance | Periodontitis | rs78382112 | A | G | 0.058 | 0.037 | 0.006 | 8.5E-09 | 1.5E-04 | 38.1 | 0.011 | 0.038 | 0.780 |
| Cognitive Performance | Periodontitis | rs7963801 | C | T | 0.548 | -0.023 | 0.003 | 3.5E-15 | 2.6E-04 | 67.4 | -0.005 | 0.017 | 0.761 |
| Cognitive Performance | Periodontitis | rs80170948 | T | G | 0.947 | 0.046 | 0.007 | 8.0E-11 | 2.1E-04 | 54.2 | -0.044 | 0.047 | 0.342 |
| Cognitive Performance | Periodontitis | rs830383 | A | G | 0.369 | 0.020 | 0.003 | 3.7E-11 | 1.8E-04 | 45.8 | -0.001 | 0.016 | 0.938 |
| Cognitive Performance | Periodontitis | rs875361 | A | G | 0.449 | -0.016 | 0.003 | 1.5E-08 | 1.3E-04 | 33.6 | 0.004 | 0.016 | 0.807 |
| Cognitive Performance | Periodontitis | rs9930063 | C | T | 0.459 | 0.016 | 0.003 | 1.4E-08 | 1.3E-04 | 34.2 | -0.003 | 0.016 | 0.850 |
| Fluid Intelligence Score | Periodontitis | rs10015590 | A | G | 0.320 | 0.046 | 0.008 | 1.3E-08 | 9.3E-04 | 138.1 | 0.015 | 0.016 | 0.364 |
| Fluid Intelligence Score | Periodontitis | rs10129426 | A | G | 0.541 | 0.046 | 0.008 | 1.5E-09 | 1.1E-03 | 157.8 | -0.014 | 0.015 | 0.352 |
| Fluid Intelligence Score | Periodontitis | rs10402747 | C | T | 0.484 | -0.042 | 0.008 | 3.5E-08 | 8.8E-04 | 131.5 | 0.003 | 0.016 | 0.854 |
| Fluid Intelligence Score | Periodontitis | rs1043254 | A | G | 0.230 | -0.054 | 0.009 | 2.7E-09 | 1.0E-03 | 153.7 | -0.002 | 0.018 | 0.930 |
| Fluid Intelligence Score | Periodontitis | rs1054442 | C | A | 0.374 | 0.047 | 0.008 | 1.7E-09 | 1.0E-03 | 155.3 | -0.026 | 0.016 | 0.112 |
| Fluid Intelligence Score | Periodontitis | rs11678980 | A | G | 0.458 | -0.055 | 0.008 | 2.5E-12 | 1.5E-03 | 223.6 | -0.002 | 0.017 | 0.892 |
| Fluid Intelligence Score | Periodontitis | rs12646225 | T | C | 0.104 | 0.076 | 0.012 | 9.9E-10 | 1.1E-03 | 159.2 | 0.007 | 0.027 | 0.792 |
| Fluid Intelligence Score | Periodontitis | rs13262595 | G | A | 0.561 | 0.047 | 0.008 | 7.6E-10 | 1.1E-03 | 162.3 | -0.008 | 0.016 | 0.618 |
| Fluid Intelligence Score | Periodontitis | rs13428598 | T | C | 0.389 | 0.044 | 0.008 | 1.2E-08 | 9.3E-04 | 139.0 | 0.000 | 0.016 | 0.992 |
| Fluid Intelligence Score | Periodontitis | rs1355620 | G | A | 0.322 | 0.052 | 0.008 | 2.4E-10 | 1.2E-03 | 176.6 | 0.016 | 0.017 | 0.352 |
| Fluid Intelligence Score | Periodontitis | rs1389994 | T | C | 0.410 | 0.047 | 0.008 | 1.3E-09 | 1.1E-03 | 158.4 | 0.003 | 0.016 | 0.868 |
| Fluid Intelligence Score | Periodontitis | rs1391438 | C | T | 0.687 | -0.046 | 0.008 | 2.5E-08 | 8.9E-04 | 133.2 | -0.007 | 0.016 | 0.665 |
| Fluid Intelligence Score | Periodontitis | rs1567307 | G | T | 0.202 | -0.053 | 0.009 | 1.6E-08 | 9.1E-04 | 135.7 | 0.017 | 0.019 | 0.368 |
| Fluid Intelligence Score | Periodontitis | rs28667297 | G | A | 0.291 | 0.054 | 0.008 | 8.9E-11 | 1.2E-03 | 180.4 | 0.020 | 0.017 | 0.229 |
| Fluid Intelligence Score | Periodontitis | rs3173680 | A | G | 0.307 | -0.048 | 0.008 | 6.1E-09 | 9.7E-04 | 144.4 | -0.003 | 0.017 | 0.848 |
| Fluid Intelligence Score | Periodontitis | rs31771 | C | T | 0.663 | -0.045 | 0.008 | 3.1E-08 | 9.0E-04 | 133.9 | 0.009 | 0.016 | 0.563 |
| Fluid Intelligence Score | Periodontitis | rs34818820 | A | G | 0.447 | -0.045 | 0.008 | 2.7E-09 | 1.0E-03 | 151.4 | 0.022 | 0.016 | 0.169 |
| Fluid Intelligence Score | Periodontitis | rs424029 | A | G | 0.043 | -0.105 | 0.019 | 1.7E-08 | 9.2E-04 | 136.6 | 0.016 | 0.041 | 0.696 |
| Fluid Intelligence Score | Periodontitis | rs4342312 | A | C | 0.378 | -0.049 | 0.008 | 2.8E-10 | 1.1E-03 | 171.0 | 0.008 | 0.016 | 0.590 |
| Fluid Intelligence Score | Periodontitis | rs4456117 | A | C | 0.509 | -0.043 | 0.008 | 1.2E-08 | 9.3E-04 | 138.9 | 0.018 | 0.016 | 0.240 |
| Fluid Intelligence Score | Periodontitis | rs4463213 | A | G | 0.554 | 0.043 | 0.008 | 1.5E-08 | 9.2E-04 | 136.8 | 0.003 | 0.015 | 0.846 |
| Fluid Intelligence Score | Periodontitis | rs4731365 | A | G | 0.405 | -0.043 | 0.008 | 1.7E-08 | 9.1E-04 | 135.6 | 0.015 | 0.016 | 0.356 |
| Fluid Intelligence Score | Periodontitis | rs4778988 | C | T | 0.313 | 0.047 | 0.008 | 9.9E-09 | 9.5E-04 | 141.4 | 0.024 | 0.017 | 0.156 |
| Fluid Intelligence Score | Periodontitis | rs4852252 | C | T | 0.565 | 0.047 | 0.008 | 7.2E-10 | 1.1E-03 | 161.9 | -0.012 | 0.016 | 0.436 |
| Fluid Intelligence Score | Periodontitis | rs506523 | C | T | 0.348 | 0.044 | 0.008 | 2.9E-08 | 8.8E-04 | 131.6 | 0.031 | 0.017 | 0.066 |
| Fluid Intelligence Score | Periodontitis | rs55837830 | T | C | 0.446 | 0.043 | 0.008 | 2.1E-08 | 9.2E-04 | 136.8 | -0.006 | 0.016 | 0.700 |
| Fluid Intelligence Score | Periodontitis | rs5758605 | A | G | 0.461 | -0.042 | 0.008 | 4.4E-08 | 8.7E-04 | 129.3 | -0.002 | 0.016 | 0.916 |
| Fluid Intelligence Score | Periodontitis | rs6125540 | T | C | 0.425 | -0.047 | 0.008 | 7.0E-10 | 1.1E-03 | 163.2 | 0.026 | 0.016 | 0.095 |
| Fluid Intelligence Score | Periodontitis | rs61768374 | G | T | 0.299 | 0.051 | 0.008 | 9.5E-10 | 1.1E-03 | 159.8 | 0.005 | 0.017 | 0.782 |
| Fluid Intelligence Score | Periodontitis | rs62098051 | G | A | 0.520 | -0.051 | 0.008 | 1.6E-11 | 1.3E-03 | 195.3 | 0.013 | 0.016 | 0.420 |
| Fluid Intelligence Score | Periodontitis | rs62183031 | C | A | 0.297 | -0.046 | 0.008 | 3.7E-08 | 8.7E-04 | 129.4 | 0.013 | 0.018 | 0.473 |
| Fluid Intelligence Score | Periodontitis | rs62268046 | G | A | 0.206 | 0.051 | 0.009 | 5.0E-08 | 8.6E-04 | 127.6 | -0.024 | 0.021 | 0.257 |
| Fluid Intelligence Score | Periodontitis | rs6436555 | C | A | 0.513 | -0.046 | 0.008 | 1.4E-09 | 1.1E-03 | 158.3 | -0.004 | 0.016 | 0.824 |
| Fluid Intelligence Score | Periodontitis | rs7010173 | T | C | 0.611 | 0.049 | 0.008 | 2.4E-10 | 1.2E-03 | 172.3 | -0.009 | 0.016 | 0.564 |
| Fluid Intelligence Score | Periodontitis | rs709322 | T | C | 0.074 | -0.081 | 0.014 | 1.8E-08 | 9.1E-04 | 135.8 | 0.053 | 0.033 | 0.112 |
| Fluid Intelligence Score | Periodontitis | rs71454591 | T | C | 0.129 | -0.063 | 0.011 | 3.1E-08 | 8.8E-04 | 131.9 | 0.001 | 0.025 | 0.963 |
| Fluid Intelligence Score | Periodontitis | rs72783406 | A | G | 0.408 | 0.047 | 0.008 | 1.7E-09 | 1.0E-03 | 155.9 | -0.008 | 0.016 | 0.641 |
| Fluid Intelligence Score | Periodontitis | rs72784651 | T | C | 0.378 | 0.046 | 0.008 | 4.2E-09 | 1.0E-03 | 150.2 | 0.016 | 0.017 | 0.325 |
| Fluid Intelligence Score | Periodontitis | rs72823192 | C | T | 0.259 | -0.054 | 0.009 | 5.7E-10 | 1.1E-03 | 165.3 | -0.020 | 0.019 | 0.281 |
| Fluid Intelligence Score | Periodontitis | rs7372302 | C | T | 0.321 | -0.069 | 0.008 | 2.7E-17 | 2.0E-03 | 305.5 | 0.009 | 0.016 | 0.587 |
| Fluid Intelligence Score | Periodontitis | rs77128898 | T | C | 0.035 | -0.114 | 0.021 | 4.8E-08 | 8.9E-04 | 133.1 | 0.093 | 0.069 | 0.176 |
| Fluid Intelligence Score | Periodontitis | rs79254826 | A | G | 0.076 | -0.079 | 0.014 | 4.6E-08 | 8.7E-04 | 130.2 | -0.039 | 0.038 | 0.302 |
| Fluid Intelligence Score | Periodontitis | rs7963801 | C | T | 0.572 | -0.050 | 0.008 | 5.5E-11 | 1.2E-03 | 186.2 | -0.005 | 0.017 | 0.761 |
| Fluid Intelligence Score | Periodontitis | rs8054111 | G | A | 0.728 | 0.050 | 0.009 | 3.8E-09 | 9.9E-04 | 148.4 | 0.008 | 0.018 | 0.658 |
| Fluid Intelligence Score | Periodontitis | rs933827 | A | G | 0.524 | 0.049 | 0.008 | 1.2E-10 | 1.2E-03 | 178.4 | -0.006 | 0.015 | 0.707 |
| Fluid Intelligence Score | Periodontitis | rs9597451 | A | G | 0.060 | -0.088 | 0.016 | 3.6E-08 | 8.8E-04 | 131.0 | 0.009 | 0.035 | 0.797 |
| Prospective Memory | Periodontitis | rs10110806 | T | C | 0.630 | -0.009 | 0.002 | 4.1E-06 | 1.4E-04 | 21.2 | 0.011 | 0.016 | 0.507 |
| Prospective Memory | Periodontitis | rs11025859 | G | T | 0.131 | 0.013 | 0.003 | 2.6E-06 | 1.4E-04 | 22.1 | -0.004 | 0.021 | 0.860 |
| Prospective Memory | Periodontitis | rs11126332 | A | G | 0.238 | 0.011 | 0.002 | 7.9E-07 | 1.6E-04 | 24.4 | 0.013 | 0.019 | 0.485 |
| Prospective Memory | Periodontitis | rs113892579 | A | G | 0.034 | 0.026 | 0.005 | 8.0E-07 | 1.6E-04 | 24.4 | -0.082 | 0.044 | 0.064 |
| Prospective Memory | Periodontitis | rs11646834 | C | T | 0.523 | -0.009 | 0.002 | 2.3E-06 | 1.5E-04 | 22.3 | -0.001 | 0.016 | 0.949 |
| Prospective Memory | Periodontitis | rs118061227 | C | T | 0.008 | 0.056 | 0.011 | 2.6E-07 | 1.7E-04 | 26.5 | -0.023 | 0.142 | 0.869 |
| Prospective Memory | Periodontitis | rs12803679 | C | A | 0.327 | 0.010 | 0.002 | 4.6E-07 | 1.7E-04 | 25.4 | 0.006 | 0.016 | 0.733 |
| Prospective Memory | Periodontitis | rs12883954 | G | T | 0.798 | 0.011 | 0.002 | 4.1E-06 | 1.4E-04 | 21.2 | 0.004 | 0.019 | 0.841 |
| Prospective Memory | Periodontitis | rs13173232 | T | C | 0.309 | -0.009 | 0.002 | 3.7E-06 | 1.4E-04 | 21.4 | 0.033 | 0.017 | 0.058 |
| Prospective Memory | Periodontitis | rs138759654 | A | C | 0.013 | 0.039 | 0.008 | 2.9E-06 | 1.4E-04 | 21.9 | -0.609 | 0.627 | 0.331 |
| Prospective Memory | Periodontitis | rs143510783 | T | C | 0.032 | -0.029 | 0.005 | 9.6E-08 | 1.9E-04 | 28.5 | 0.053 | 0.046 | 0.250 |
| Prospective Memory | Periodontitis | rs144490799 | T | C | 0.020 | 0.034 | 0.007 | 3.4E-06 | 1.4E-04 | 21.6 | 0.144 | 0.120 | 0.230 |
| Prospective Memory | Periodontitis | rs145625753 | T | C | 0.032 | 0.026 | 0.006 | 4.9E-06 | 1.4E-04 | 20.9 | -0.018 | 0.074 | 0.804 |
| Prospective Memory | Periodontitis | rs17001547 | C | T | 0.024 | -0.029 | 0.006 | 3.0E-06 | 1.4E-04 | 21.8 | -0.029 | 0.049 | 0.552 |
| Prospective Memory | Periodontitis | rs17106063 | A | G | 0.162 | 0.012 | 0.003 | 1.9E-06 | 1.5E-04 | 22.7 | -0.032 | 0.023 | 0.172 |
| Prospective Memory | Periodontitis | rs17583177 | T | C | 0.074 | 0.018 | 0.004 | 6.6E-07 | 1.6E-04 | 24.7 | -0.035 | 0.040 | 0.386 |
| Prospective Memory | Periodontitis | rs187507943 | G | A | 0.047 | 0.021 | 0.005 | 2.9E-06 | 1.4E-04 | 21.9 | 0.022 | 0.039 | 0.563 |
| Prospective Memory | Periodontitis | rs27943 | C | T | 0.939 | -0.018 | 0.004 | 3.9E-06 | 1.4E-04 | 21.3 | 0.034 | 0.027 | 0.202 |
| Prospective Memory | Periodontitis | rs28483211 | C | T | 0.025 | 0.028 | 0.006 | 3.3E-06 | 1.4E-04 | 21.7 | 0.003 | 0.056 | 0.959 |
| Prospective Memory | Periodontitis | rs28537494 | C | T | 0.145 | -0.013 | 0.003 | 3.0E-06 | 1.4E-04 | 21.8 | -0.022 | 0.023 | 0.329 |
| Prospective Memory | Periodontitis | rs2925944 | A | G | 0.021 | 0.034 | 0.007 | 8.6E-07 | 1.6E-04 | 24.2 | -0.038 | 0.075 | 0.613 |
| Prospective Memory | Periodontitis | rs455771 | G | A | 0.780 | -0.011 | 0.002 | 3.9E-06 | 1.4E-04 | 21.3 | -0.019 | 0.018 | 0.306 |
| Prospective Memory | Periodontitis | rs4934572 | C | A | 0.018 | 0.033 | 0.007 | 2.2E-06 | 1.5E-04 | 22.4 | -0.044 | 0.039 | 0.259 |
| Prospective Memory | Periodontitis | rs55701287 | T | C | 0.098 | 0.015 | 0.003 | 1.8E-06 | 1.5E-04 | 22.8 | -0.001 | 0.027 | 0.981 |
| Prospective Memory | Periodontitis | rs56223232 | A | G | 0.045 | 0.021 | 0.005 | 4.9E-06 | 1.4E-04 | 20.9 | -0.025 | 0.034 | 0.472 |
| Prospective Memory | Periodontitis | rs60350712 | G | A | 0.015 | 0.038 | 0.008 | 9.6E-07 | 1.6E-04 | 24.0 | -0.026 | 0.063 | 0.685 |
| Prospective Memory | Periodontitis | rs61957386 | A | G | 0.049 | 0.022 | 0.004 | 9.5E-07 | 1.6E-04 | 24.0 | 0.012 | 0.042 | 0.781 |
| Prospective Memory | Periodontitis | rs62009918 | T | C | 0.234 | -0.015 | 0.002 | 3.2E-11 | 2.9E-04 | 44.1 | 0.016 | 0.019 | 0.409 |
| Prospective Memory | Periodontitis | rs62397635 | A | G | 0.099 | 0.016 | 0.003 | 7.2E-07 | 1.6E-04 | 24.6 | -0.044 | 0.029 | 0.129 |
| Prospective Memory | Periodontitis | rs6702781 | C | T | 0.012 | -0.044 | 0.009 | 4.2E-07 | 1.7E-04 | 25.6 | 0.160 | 0.120 | 0.185 |
| Prospective Memory | Periodontitis | rs73040360 | T | C | 0.147 | 0.014 | 0.003 | 4.7E-07 | 1.7E-04 | 25.4 | 0.009 | 0.024 | 0.721 |
| Prospective Memory | Periodontitis | rs73093577 | T | C | 0.123 | -0.014 | 0.003 | 6.0E-07 | 1.6E-04 | 24.9 | -0.008 | 0.024 | 0.753 |
| Prospective Memory | Periodontitis | rs73424022 | A | G | 0.282 | -0.010 | 0.002 | 1.2E-06 | 1.5E-04 | 23.6 | 0.041 | 0.017 | 0.016 |
| Prospective Memory | Periodontitis | rs7342602 | C | T | 0.816 | 0.011 | 0.002 | 4.9E-06 | 1.4E-04 | 20.9 | -0.005 | 0.019 | 0.801 |
| Prospective Memory | Periodontitis | rs77309031 | G | T | 0.042 | 0.023 | 0.005 | 1.5E-06 | 1.5E-04 | 23.2 | 0.051 | 0.041 | 0.211 |
| Prospective Memory | Periodontitis | rs8039474 | T | C | 0.938 | -0.020 | 0.004 | 4.6E-07 | 1.7E-04 | 25.4 | 0.018 | 0.034 | 0.586 |
| Prospective Memory | Periodontitis | rs9549218 | A | G | 0.093 | -0.015 | 0.003 | 4.8E-06 | 1.4E-04 | 20.9 | 0.023 | 0.035 | 0.517 |
| Prospective Memory | Periodontitis | rs9858813 | A | G | 0.094 | -0.016 | 0.003 | 5.9E-07 | 1.6E-04 | 25.0 | -0.039 | 0.027 | 0.143 |
| Reaction Time | Periodontitis | rs1054442 | C | A | 0.000 | 0.012 | 0.002 | 2.4E-12 | 1.5E-04 | 49.1 | -0.026 | 0.016 | 0.112 |
| Reaction Time | Periodontitis | rs10911301 | G | A | 0.000 | -0.010 | 0.002 | 1.8E-09 | 1.1E-04 | 36.1 | 0.028 | 0.016 | 0.076 |
| Reaction Time | Periodontitis | rs13028903 | T | C | 0.000 | 0.013 | 0.002 | 1.7E-14 | 1.8E-04 | 58.8 | 0.018 | 0.016 | 0.272 |
| Reaction Time | Periodontitis | rs13219424 | T | C | 0.000 | -0.010 | 0.002 | 1.3E-08 | 9.8E-05 | 32.3 | 0.018 | 0.017 | 0.308 |
| Reaction Time | Periodontitis | rs1351848 | T | C | 0.000 | 0.011 | 0.002 | 9.5E-10 | 1.1E-04 | 37.4 | -0.007 | 0.016 | 0.651 |
| Reaction Time | Periodontitis | rs1385253 | C | T | 0.000 | -0.010 | 0.002 | 1.7E-08 | 9.6E-05 | 31.8 | 0.008 | 0.018 | 0.674 |
| Reaction Time | Periodontitis | rs16822665 | T | C | 0.000 | -0.010 | 0.002 | 4.9E-09 | 1.0E-04 | 34.2 | -0.008 | 0.016 | 0.622 |
| Reaction Time | Periodontitis | rs16959783 | T | C | 0.000 | 0.010 | 0.002 | 1.8E-08 | 9.6E-05 | 31.7 | 0.025 | 0.020 | 0.195 |
| Reaction Time | Periodontitis | rs1734197 | T | C | 0.000 | 0.010 | 0.002 | 2.2E-08 | 9.5E-05 | 31.3 | 0.000 | 0.016 | 0.979 |
| Reaction Time | Periodontitis | rs2040879 | C | T | 0.000 | 0.011 | 0.002 | 3.3E-10 | 1.2E-04 | 39.5 | 0.013 | 0.020 | 0.515 |
| Reaction Time | Periodontitis | rs2604268 | T | C | 0.000 | 0.012 | 0.002 | 9.2E-12 | 1.4E-04 | 46.5 | -0.015 | 0.018 | 0.401 |
| Reaction Time | Periodontitis | rs323299 | T | G | 0.000 | -0.010 | 0.002 | 1.3E-08 | 9.8E-05 | 32.3 | 0.017 | 0.016 | 0.289 |
| Reaction Time | Periodontitis | rs35066740 | T | G | 0.000 | 0.010 | 0.002 | 3.7E-09 | 1.1E-04 | 34.8 | 0.013 | 0.020 | 0.521 |
| Reaction Time | Periodontitis | rs4627212 | G | A | 0.000 | 0.010 | 0.002 | 1.9E-09 | 1.1E-04 | 36.1 | 0.019 | 0.017 | 0.251 |
| Reaction Time | Periodontitis | rs4673905 | G | A | 0.000 | 0.012 | 0.002 | 4.0E-12 | 1.5E-04 | 48.1 | -0.007 | 0.017 | 0.682 |
| Reaction Time | Periodontitis | rs61786416 | G | A | 0.000 | 0.010 | 0.002 | 1.7E-08 | 9.7E-05 | 31.9 | 0.013 | 0.027 | 0.625 |
| Reaction Time | Periodontitis | rs6870103 | T | G | 0.000 | 0.010 | 0.002 | 1.9E-09 | 1.1E-04 | 36.1 | 0.002 | 0.015 | 0.874 |
| Reaction Time | Periodontitis | rs7075591 | A | G | 0.000 | 0.010 | 0.002 | 1.3E-08 | 9.8E-05 | 32.3 | 0.006 | 0.017 | 0.739 |
| Reaction Time | Periodontitis | rs75543711 | A | G | 0.000 | -0.009 | 0.002 | 4.6E-08 | 9.1E-05 | 29.9 | 0.006 | 0.025 | 0.809 |
| Reaction Time | Periodontitis | rs77998199 | G | A | 0.000 | 0.011 | 0.002 | 2.0E-10 | 1.2E-04 | 40.5 | -0.007 | 0.020 | 0.728 |
| Reaction Time | Periodontitis | rs925229 | G | A | 0.000 | 0.010 | 0.002 | 4.7E-08 | 9.0E-05 | 29.9 | -0.031 | 0.016 | 0.050 |
| Reaction Time | Periodontitis | rs936414 | G | A | 0.000 | 0.010 | 0.002 | 4.9E-08 | 9.0E-05 | 29.7 | -0.008 | 0.017 | 0.622 |
| Alzheimer's disease | Periodontitis | rs10792832 | A | G | 0.358 | -0.106 | 0.008 | 6.3E-36 | 5.1E-03 | 2511.2 | -0.007 | 0.016 | 0.643 |
| Alzheimer's disease | Periodontitis | rs11218343 | T | C | 0.961 | 0.165 | 0.021 | 1.0E-14 | 2.1E-03 | 1001.8 | 0.044 | 0.036 | 0.222 |
| Alzheimer's disease | Periodontitis | rs112481437 | A | G | 0.028 | 0.294 | 0.028 | 4.9E-26 | 4.7E-03 | 2304.7 | -0.094 | 0.098 | 0.337 |
| Alzheimer's disease | Periodontitis | rs117618017 | T | C | 0.144 | 0.113 | 0.012 | 1.7E-21 | 3.1E-03 | 1538.6 | -0.010 | 0.029 | 0.727 |
| Alzheimer's disease | Periodontitis | rs11769559 | T | C | 0.594 | 0.070 | 0.009 | 4.0E-16 | 2.3E-03 | 1138.3 | 0.247 | 0.124 | 0.046 |
| Alzheimer's disease | Periodontitis | rs12151021 | A | G | 0.336 | 0.106 | 0.009 | 4.1E-30 | 5.0E-03 | 2432.2 | -0.032 | 0.018 | 0.078 |
| Alzheimer's disease | Periodontitis | rs12444183 | A | G | 0.387 | -0.059 | 0.008 | 2.2E-12 | 1.7E-03 | 809.4 | -0.005 | 0.016 | 0.732 |
| Alzheimer's disease | Periodontitis | rs12590654 | A | G | 0.328 | -0.069 | 0.009 | 2.1E-15 | 2.1E-03 | 1034.1 | -0.003 | 0.017 | 0.878 |
| Alzheimer's disease | Periodontitis | rs143332484 | T | C | 0.013 | 0.335 | 0.038 | 6.0E-19 | 2.8E-03 | 1361.9 | -0.067 | 0.970 | 0.945 |
| Alzheimer's disease | Periodontitis | rs1582763 | A | G | 0.371 | -0.086 | 0.008 | 1.7E-24 | 3.5E-03 | 1688.6 | 0.008 | 0.016 | 0.633 |
| Alzheimer's disease | Periodontitis | rs1693551 | T | C | 0.533 | -0.046 | 0.008 | 1.8E-08 | 1.0E-03 | 511.9 | 0.026 | 0.015 | 0.087 |
| Alzheimer's disease | Periodontitis | rs190651665 | T | G | 0.970 | 0.160 | 0.026 | 8.4E-10 | 1.5E-03 | 723.3 | -0.012 | 0.048 | 0.795 |
| Alzheimer's disease | Periodontitis | rs2070902 | T | C | 0.254 | -0.054 | 0.009 | 1.0E-08 | 1.1E-03 | 533.2 | 0.011 | 0.017 | 0.509 |
| Alzheimer's disease | Periodontitis | rs2154481 | T | C | 0.524 | 0.050 | 0.008 | 1.0E-09 | 1.2E-03 | 608.8 | 0.005 | 0.016 | 0.759 |
| Alzheimer's disease | Periodontitis | rs2526377 | A | G | 0.555 | 0.045 | 0.008 | 4.1E-08 | 1.0E-03 | 492.4 | 0.017 | 0.016 | 0.275 |
| Alzheimer's disease | Periodontitis | rs2830489 | T | C | 0.281 | -0.055 | 0.009 | 1.7E-09 | 1.2E-03 | 590.0 | 0.015 | 0.018 | 0.398 |
| Alzheimer's disease | Periodontitis | rs28577986 | A | G | 0.263 | 0.070 | 0.011 | 3.4E-10 | 1.9E-03 | 923.0 | 0.064 | 0.032 | 0.043 |
| Alzheimer's disease | Periodontitis | rs365653 | A | G | 0.892 | 0.256 | 0.015 | 1.8E-68 | 1.3E-02 | 6244.8 | -0.029 | 0.024 | 0.228 |
| Alzheimer's disease | Periodontitis | rs4292 | T | C | 0.617 | 0.069 | 0.008 | 3.5E-16 | 2.2E-03 | 1093.1 | -0.003 | 0.017 | 0.867 |
| Alzheimer's disease | Periodontitis | rs4485362 | T | G | 0.540 | 0.046 | 0.008 | 2.0E-08 | 1.1E-03 | 519.8 | 0.030 | 0.017 | 0.070 |
| Alzheimer's disease | Periodontitis | rs4714447 | T | C | 0.345 | 0.058 | 0.009 | 1.7E-11 | 1.5E-03 | 737.1 | -0.041 | 0.017 | 0.014 |
| Alzheimer's disease | Periodontitis | rs57402520 | A | G | 0.122 | 0.091 | 0.013 | 6.6E-13 | 1.8E-03 | 855.1 | -0.041 | 0.024 | 0.090 |
| Alzheimer's disease | Periodontitis | rs5848 | T | C | 0.289 | 0.065 | 0.009 | 1.8E-12 | 1.7E-03 | 836.8 | -0.023 | 0.018 | 0.209 |
| Alzheimer's disease | Periodontitis | rs593742 | A | G | 0.705 | 0.061 | 0.009 | 1.0E-11 | 1.5E-03 | 755.4 | -0.022 | 0.016 | 0.190 |
| Alzheimer's disease | Periodontitis | rs6014724 | A | G | 0.910 | 0.118 | 0.015 | 4.8E-16 | 2.3E-03 | 1104.6 | -0.037 | 0.026 | 0.143 |
| Alzheimer's disease | Periodontitis | rs61762319 | A | G | 0.974 | -0.143 | 0.026 | 2.1E-08 | 1.0E-03 | 511.1 | 0.058 | 0.068 | 0.389 |
| Alzheimer's disease | Periodontitis | rs62375397 | T | C | 0.210 | 0.075 | 0.010 | 8.6E-14 | 1.8E-03 | 903.1 | -0.039 | 0.019 | 0.044 |
| Alzheimer's disease | Periodontitis | rs6586028 | T | C | 0.804 | 0.079 | 0.010 | 1.3E-14 | 2.0E-03 | 964.7 | -0.044 | 0.020 | 0.027 |
| Alzheimer's disease | Periodontitis | rs6656401 | A | G | 0.188 | 0.125 | 0.010 | 2.8E-33 | 4.8E-03 | 2349.1 | -0.010 | 0.021 | 0.630 |
| Alzheimer's disease | Periodontitis | rs67250450 | T | C | 0.788 | 0.056 | 0.010 | 2.0E-08 | 1.0E-03 | 510.4 | 0.020 | 0.018 | 0.255 |
| Alzheimer's disease | Periodontitis | rs6733839 | T | C | 0.389 | 0.169 | 0.008 | 6.5E-90 | 1.4E-02 | 6678.3 | 0.000 | 0.017 | 0.983 |
| Alzheimer's disease | Periodontitis | rs6846529 | T | C | 0.717 | -0.067 | 0.009 | 1.3E-13 | 1.8E-03 | 897.0 | -0.006 | 0.017 | 0.723 |
| Alzheimer's disease | Periodontitis | rs7068231 | T | G | 0.403 | -0.049 | 0.008 | 6.8E-09 | 1.1E-03 | 556.8 | -0.003 | 0.016 | 0.861 |
| Alzheimer's disease | Periodontitis | rs73223431 | T | C | 0.369 | 0.066 | 0.008 | 5.3E-15 | 2.0E-03 | 979.4 | -0.033 | 0.016 | 0.046 |
| Alzheimer's disease | Periodontitis | rs7384878 | T | C | 0.690 | 0.078 | 0.009 | 2.1E-18 | 2.6E-03 | 1255.9 | 0.015 | 0.017 | 0.392 |
| Alzheimer's disease | Periodontitis | rs74504435 | A | G | 0.907 | 0.084 | 0.014 | 2.0E-09 | 1.2E-03 | 586.6 | -0.002 | 0.029 | 0.934 |
| Alzheimer's disease | Periodontitis | rs74685827 | T | G | 0.981 | -0.196 | 0.030 | 8.6E-11 | 1.4E-03 | 687.5 | -0.080 | 0.064 | 0.212 |
| Alzheimer's disease | Periodontitis | rs74745468 | A | G | 0.088 | 0.082 | 0.015 | 1.4E-08 | 1.1E-03 | 526.6 | -0.047 | 0.031 | 0.128 |
| Alzheimer's disease | Periodontitis | rs7912495 | A | G | 0.538 | -0.057 | 0.008 | 2.9E-12 | 1.6E-03 | 794.2 | 0.011 | 0.016 | 0.503 |
| Alzheimer's disease | Periodontitis | rs867230 | A | C | 0.603 | 0.101 | 0.008 | 1.5E-33 | 4.9E-03 | 2387.7 | -0.005 | 0.016 | 0.758 |
| Alzheimer's disease | Periodontitis | rs9676738 | A | G | 0.042 | 0.169 | 0.023 | 1.2E-13 | 2.3E-03 | 1119.2 | -0.055 | 0.088 | 0.530 |
| Alzheimer's disease | Periodontitis | rs976271 | A | G | 0.361 | 0.047 | 0.008 | 3.2E-08 | 1.0E-03 | 491.2 | 0.026 | 0.016 | 0.102 |
| Early-onset AD | Periodontitis | rs10198199 | A | G | 0.836 | 0.243 | 0.053 | 4.9E-06 | 1.6E-02 | 3046.4 | 0.001 | 0.021 | 0.976 |
| Early-onset AD | Periodontitis | rs10268827 | G | A | 0.280 | -0.223 | 0.044 | 4.0E-07 | 2.0E-02 | 3787.0 | -0.012 | 0.020 | 0.539 |
| Early-onset AD | Periodontitis | rs113033863 | C | T | 0.011 | 0.766 | 0.154 | 6.5E-07 | 1.3E-02 | 2423.6 | 0.118 | 0.112 | 0.292 |
| Early-onset AD | Periodontitis | rs113097545 | G | T | 0.091 | 0.288 | 0.062 | 3.5E-06 | 1.4E-02 | 2569.3 | -0.039 | 0.033 | 0.233 |
| Early-onset AD | Periodontitis | rs11735752 | T | C | 0.126 | 0.274 | 0.055 | 6.0E-07 | 1.7E-02 | 3115.3 | 0.023 | 0.028 | 0.416 |
| Early-onset AD | Periodontitis | rs11879248 | C | T | 0.633 | -0.255 | 0.039 | 7.0E-11 | 3.0E-02 | 5791.6 | 0.002 | 0.017 | 0.923 |
| Early-onset AD | Periodontitis | rs142875505 | A | C | 0.014 | 0.651 | 0.141 | 3.9E-06 | 1.2E-02 | 2157.3 | 0.109 | 0.090 | 0.228 |
| Early-onset AD | Periodontitis | rs1586166 | T | C | 0.567 | -0.177 | 0.038 | 3.9E-06 | 1.5E-02 | 2878.7 | 0.024 | 0.016 | 0.123 |
| Early-onset AD | Periodontitis | rs2599437 | C | T | 0.356 | -0.200 | 0.040 | 6.4E-07 | 1.8E-02 | 3451.2 | -0.038 | 0.017 | 0.025 |
| Early-onset AD | Periodontitis | rs429358 | C | T | 0.179 | 1.066 | 0.040 | 3.5E-155 | 3.3E-01 | 92894.7 | -0.011 | 0.023 | 0.622 |
| Early-onset AD | Periodontitis | rs56368748 | A | G | 0.320 | 0.210 | 0.040 | 2.0E-07 | 1.9E-02 | 3636.9 | -0.005 | 0.018 | 0.788 |
| Early-onset AD | Periodontitis | rs647235 | G | A | 0.423 | -0.183 | 0.039 | 2.9E-06 | 1.6E-02 | 3070.8 | -0.005 | 0.016 | 0.754 |
| Early-onset AD | Periodontitis | rs72635719 | T | C | 0.018 | 0.585 | 0.126 | 3.3E-06 | 1.2E-02 | 2222.4 | -0.003 | 0.071 | 0.969 |
| Early-onset AD | Periodontitis | rs73387962 | G | A | 0.021 | 0.557 | 0.116 | 1.7E-06 | 1.3E-02 | 2430.0 | 0.013 | 0.034 | 0.690 |
| Early-onset AD | Periodontitis | rs7581359 | T | C | 0.073 | 0.317 | 0.069 | 3.9E-06 | 1.4E-02 | 2543.1 | -0.001 | 0.026 | 0.986 |
| Early-onset AD | Periodontitis | rs7616279 | T | C | 0.318 | -0.238 | 0.051 | 3.1E-06 | 2.4E-02 | 4645.9 | 0.006 | 0.031 | 0.845 |
| Early-onset AD | Periodontitis | rs78802426 | C | A | 0.047 | 0.480 | 0.080 | 2.1E-09 | 2.1E-02 | 3925.4 | -0.156 | 0.196 | 0.425 |
| Late-onset AD | Periodontitis | rs10914268 | T | G | 0.558 | -0.090 | 0.020 | 4.0E-06 | 4.0E-03 | 772.5 | -0.003 | 0.016 | 0.873 |
| Late-onset AD | Periodontitis | rs11185103 | A | C | 0.397 | -0.093 | 0.020 | 4.9E-06 | 4.2E-03 | 799.4 | 0.003 | 0.019 | 0.871 |
| Late-onset AD | Periodontitis | rs112866871 | G | A | 0.032 | -0.303 | 0.059 | 3.1E-07 | 5.8E-03 | 1105.1 | -0.002 | 0.062 | 0.972 |
| Late-onset AD | Periodontitis | rs1132899 | C | T | 0.555 | 0.241 | 0.020 | 2.2E-34 | 2.9E-02 | 5622.7 | 0.003 | 0.017 | 0.849 |
| Late-onset AD | Periodontitis | rs115403653 | T | C | 0.026 | 0.267 | 0.058 | 4.0E-06 | 3.7E-03 | 701.9 | 0.009 | 0.066 | 0.897 |
| Late-onset AD | Periodontitis | rs117090603 | A | C | 0.027 | -0.321 | 0.066 | 1.0E-06 | 5.4E-03 | 1045.6 | -0.008 | 0.042 | 0.854 |
| Late-onset AD | Periodontitis | rs147918179 | T | C | 0.027 | -0.346 | 0.064 | 7.5E-08 | 6.2E-03 | 1195.4 | 0.102 | 0.082 | 0.212 |
| Late-onset AD | Periodontitis | rs17190727 | A | C | 0.464 | -0.101 | 0.020 | 2.4E-07 | 5.1E-03 | 970.3 | 0.009 | 0.016 | 0.583 |
| Late-onset AD | Periodontitis | rs1721804 | G | A | 0.792 | 0.116 | 0.025 | 2.6E-06 | 4.4E-03 | 850.3 | -0.021 | 0.018 | 0.240 |
| Late-onset AD | Periodontitis | rs17650574 | C | T | 0.033 | -0.275 | 0.056 | 1.0E-06 | 4.9E-03 | 932.1 | 0.015 | 0.042 | 0.722 |
| Late-onset AD | Periodontitis | rs180719460 | A | G | 0.025 | -0.330 | 0.067 | 9.5E-07 | 5.4E-03 | 1033.8 | -0.124 | 0.078 | 0.111 |
| Late-onset AD | Periodontitis | rs189888235 | A | G | 0.015 | 0.356 | 0.077 | 3.7E-06 | 3.7E-03 | 712.9 | -0.075 | 0.077 | 0.330 |
| Late-onset AD | Periodontitis | rs2279590 | C | T | 0.578 | 0.094 | 0.020 | 1.9E-06 | 4.3E-03 | 831.8 | -0.001 | 0.016 | 0.967 |
| Late-onset AD | Periodontitis | rs2470890 | C | T | 0.434 | -0.111 | 0.020 | 1.9E-08 | 6.0E-03 | 1153.6 | -0.036 | 0.016 | 0.027 |
| Late-onset AD | Periodontitis | rs28615360 | A | G | 0.012 | 0.788 | 0.079 | 1.5E-23 | 1.5E-02 | 2835.9 | -0.004 | 0.069 | 0.954 |
| Late-onset AD | Periodontitis | rs34173062 | A | G | 0.056 | 0.217 | 0.042 | 2.3E-07 | 5.0E-03 | 952.4 | 0.107 | 0.075 | 0.155 |
| Late-onset AD | Periodontitis | rs4315653 | A | G | 0.110 | -0.146 | 0.032 | 4.1E-06 | 4.2E-03 | 808.7 | -0.006 | 0.020 | 0.775 |
| Late-onset AD | Periodontitis | rs457150 | C | T | 0.657 | 0.097 | 0.021 | 2.5E-06 | 4.2E-03 | 814.1 | 0.021 | 0.016 | 0.192 |
| Late-onset AD | Periodontitis | rs4683295 | G | A | 0.317 | 0.098 | 0.021 | 3.6E-06 | 4.2E-03 | 802.6 | 0.023 | 0.016 | 0.159 |
| Late-onset AD | Periodontitis | rs4789278 | A | G | 0.123 | 0.154 | 0.029 | 1.3E-07 | 5.1E-03 | 975.9 | -0.007 | 0.021 | 0.729 |
| Late-onset AD | Periodontitis | rs5848 | T | C | 0.344 | 0.124 | 0.020 | 1.2E-09 | 6.9E-03 | 1335.2 | -0.023 | 0.018 | 0.209 |
| Late-onset AD | Periodontitis | rs60949950 | A | G | 0.086 | 0.159 | 0.034 | 4.3E-06 | 3.9E-03 | 754.4 | -0.004 | 0.029 | 0.892 |
| Late-onset AD | Periodontitis | rs611267 | G | A | 0.682 | 0.141 | 0.021 | 2.5E-11 | 8.7E-03 | 1669.0 | -0.011 | 0.015 | 0.496 |
| Late-onset AD | Periodontitis | rs61972596 | T | G | 0.152 | -0.141 | 0.028 | 5.5E-07 | 5.1E-03 | 978.3 | -0.016 | 0.020 | 0.431 |
| Late-onset AD | Periodontitis | rs61977322 | C | T | 0.177 | -0.145 | 0.026 | 2.2E-08 | 6.1E-03 | 1177.7 | -0.016 | 0.020 | 0.433 |
| Late-onset AD | Periodontitis | rs6733839 | T | C | 0.373 | 0.132 | 0.020 | 6.5E-11 | 8.2E-03 | 1581.0 | 0.000 | 0.017 | 0.983 |
| Late-onset AD | Periodontitis | rs679515 | C | T | 0.807 | -0.142 | 0.024 | 5.6E-09 | 6.3E-03 | 1203.9 | 0.003 | 0.021 | 0.871 |
| Late-onset AD | Periodontitis | rs72835104 | G | A | 0.075 | -0.194 | 0.039 | 5.8E-07 | 5.2E-03 | 999.1 | -0.017 | 0.031 | 0.586 |
| Late-onset AD | Periodontitis | rs7657608 | T | C | 0.273 | 0.116 | 0.022 | 9.1E-08 | 5.3E-03 | 1025.4 | 0.009 | 0.017 | 0.585 |
| Late-onset AD | Periodontitis | rs77196557 | A | G | 0.047 | 0.205 | 0.044 | 3.9E-06 | 3.8E-03 | 724.1 | -0.017 | 0.066 | 0.794 |
| Late-onset AD | Periodontitis | rs78600181 | T | C | 0.005 | -0.731 | 0.154 | 2.1E-06 | 5.7E-03 | 1097.1 | -0.108 | 0.070 | 0.125 |
| Late-onset AD | Periodontitis | rs7912495 | G | A | 0.495 | 0.096 | 0.020 | 9.6E-07 | 4.6E-03 | 881.2 | -0.011 | 0.016 | 0.503 |
| Late-onset AD | Periodontitis | rs8113128 | C | A | 0.043 | 0.451 | 0.045 | 7.4E-24 | 1.7E-02 | 3257.2 | -0.048 | 0.049 | 0.325 |
| Late-onset AD | Periodontitis | rs9468 | C | T | 0.097 | -0.215 | 0.035 | 5.7E-10 | 8.1E-03 | 1557.1 | 0.010 | 0.029 | 0.725 |
| Lewy body dementia | Periodontitis | rs2230288 | T | C | 0.009 | 1.061 | 0.149 | 1.3E-12 | 2.0E-02 | 136.5 | -0.042 | 0.078 | 0.587 |
| Lewy body dementia | Periodontitis | rs6599388 | T | C | 0.310 | 0.220 | 0.040 | 3.5E-08 | 2.1E-02 | 140.5 | -0.010 | 0.017 | 0.577 |
| Lewy body dementia | Periodontitis | rs6733839 | T | C | 0.362 | 0.227 | 0.039 | 4.2E-09 | 2.4E-02 | 160.5 | 0.000 | 0.017 | 0.983 |
| Vascular Dementia | Periodontitis | rs10408790 | A | G | 0.430 | -0.311 | 0.068 | 4.3E-06 | 4.7E-02 | 20403.6 | 0.031 | 0.019 | 0.101 |
| Vascular Dementia | Periodontitis | rs36038448 | T | C | 0.056 | 1.021 | 0.222 | 4.0E-06 | 1.1E-01 | 51241.7 | -0.029 | 0.089 | 0.749 |
| Vascular Dementia | Periodontitis | rs529543 | G | T | 0.395 | -0.312 | 0.068 | 4.7E-06 | 4.6E-02 | 20010.9 | -0.022 | 0.016 | 0.149 |
| Vascular Dementia | Periodontitis | rs77422059 | A | G | 0.065 | 0.970 | 0.208 | 3.0E-06 | 1.1E-01 | 52976.5 | -0.002 | 0.058 | 0.970 |
| Vascular Dementia | Periodontitis | rs79891963 | T | C | 0.056 | 1.128 | 0.232 | 1.2E-06 | 1.3E-01 | 63344.6 | 0.002 | 0.046 | 0.968 |
| Frontotemporal Dementia | Periodontitis | rs73148325 | C | A | 0.212 | -0.726 | 0.158 | 4.5E-06 | 1.8E-01 | 84067.1 | 0.001 | 0.020 | 0.947 |
| Frontotemporal Dementia | Periodontitis | rs79130292 | A | G | 0.048 | 0.905 | 0.197 | 4.5E-06 | 7.5E-02 | 31602.7 | -0.090 | 0.056 | 0.104 |
|  |  |  |  |  |  |  |  |  |  |  |  |  |  |
| **Change rate in brain structure** | |  |  |  |  |  |  |  |  |  |  |  |  |
| Cortical thickness | Periodontitis | rs17006251 | A | C | 0.202 | -0.965 | 0.205 | 2.4E-06 | 1.4E-03 | 22.2 | -0.005 | 0.020 | 0.786 |
| Cortical thickness | Periodontitis | rs564158 | A | G | 0.826 | 1.025 | 0.216 | 2.1E-06 | 1.4E-03 | 22.5 | -0.009 | 0.019 | 0.648 |
| Cortical thickness | Periodontitis | rs595269 | A | C | 0.931 | 1.794 | 0.382 | 2.6E-06 | 1.4E-03 | 22.1 | 0.015 | 0.035 | 0.672 |
| Cortical thickness | Periodontitis | rs6435231 | T | C | 0.329 | -0.957 | 0.190 | 4.9E-07 | 1.6E-03 | 25.3 | 0.018 | 0.017 | 0.286 |
| Cortical thickness | Periodontitis | rs72698183 | T | G | 0.261 | -0.942 | 0.195 | 1.4E-06 | 1.5E-03 | 23.3 | 0.028 | 0.019 | 0.134 |
| Cortical thickness | Periodontitis | rs75595201 | T | C | 0.939 | 1.824 | 0.392 | 3.3E-06 | 1.4E-03 | 21.6 | 0.026 | 0.037 | 0.487 |
| Brain surface area | Periodontitis | rs11955994 | A | G | 0.159 | -58.727 | 12.03 | 1.1E-06 | 1.5E-03 | 23.8 | -0.030 | 0.022 | 0.166 |
| Brain surface area | Periodontitis | rs4740274 | A | G | 0.527 | 31.060 | 6.750 | 4.2E-06 | 1.4E-03 | 21.2 | 0.003 | 0.018 | 0.887 |
| Brain surface area | Periodontitis | rs58313139 | T | C | 0.071 | 62.257 | 12.83 | 1.2E-06 | 1.5E-03 | 23.5 | 0.027 | 0.031 | 0.383 |
| Brain surface area | Periodontitis | rs61895068 | A | G | 0.879 | -48.160 | 9.909 | 1.2E-06 | 1.5E-03 | 23.6 | 0.015 | 0.025 | 0.561 |
| Brain surface area | Periodontitis | rs66620635 | T | C | 0.131 | -54.004 | 11.19 | 1.4E-06 | 1.5E-03 | 23.3 | 0.034 | 0.023 | 0.143 |
| Hippocampal volume | Periodontitis | rs1913699 | A | C | 0.467 | -3.908 | 0.827 | 2.3E-06 | 1.4E-03 | 22.3 | -0.015 | 0.015 | 0.318 |
| Hippocampal volume | Periodontitis | rs4321178 | T | C | 0.560 | -4.915 | 1.076 | 5.0E-06 | 1.3E-03 | 20.9 | 0.051 | 0.117 | 0.663 |
| Total brain volume | Periodontitis | rs10790497 | A | G | 0.654 | 262.52 | 56.89 | 4.0E-06 | 1.4E-03 | 21.3 | -0.007 | 0.017 | 0.686 |
| Total brain volume | Periodontitis | rs12325429 | A | G | 0.433 | -303.84 | 55.34 | 4.0E-08 | 1.9E-03 | 30.1 | -0.010 | 0.016 | 0.530 |
| Total brain volume | Periodontitis | rs1532759 | T | C | 0.133 | 421.76 | 92.25 | 4.8E-06 | 1.3E-03 | 20.9 | 0.009 | 0.035 | 0.793 |
| Total brain volume | Periodontitis | rs1880248 | T | C | 0.800 | 349.83 | 71.37 | 9.5E-07 | 1.5E-03 | 24.0 | -0.005 | 0.020 | 0.816 |
| Total brain volume | Periodontitis | rs4141409 | T | G | 0.369 | -259.51 | 55.87 | 3.4E-06 | 1.4E-03 | 21.6 | 0.001 | 0.016 | 0.931 |
| Total brain volume | Periodontitis | rs55833149 | T | G | 0.133 | -405.3 | 84.94 | 1.8E-06 | 1.5E-03 | 22.8 | 0.061 | 0.022 | 0.007 |
| Total brain volume | Periodontitis | rs61804371 | T | C | 0.882 | 405.08 | 87.32 | 3.5E-06 | 1.4E-03 | 21.5 | 0.014 | 0.025 | 0.582 |
| Total brain volume | Periodontitis | rs71612691 | A | C | 0.137 | 380.75 | 80.93 | 2.5E-06 | 1.4E-03 | 22.1 | -0.023 | 0.027 | 0.382 |
| Total brain volume | Periodontitis | rs7601178 | A | G | 0.622 | -275.60 | 59.80 | 4.1E-06 | 1.4E-03 | 21.2 | -0.003 | 0.121 | 0.982 |
|  |  |  |  |  |  |  |  |  |  |  |  |  |  |
| **Slope of cognitive decline** | |  |  |  |  |  |  |  |  |  |  |  |  |
| Executive function | Periodontitis | rs10004897 | A | G |  | 0.250 | 0.054 | 4.4E-06 | 1.8E-02 | 20.5 | -0.009 | 0.021 | 0.668 |
| Executive function | Periodontitis | rs2546753 | A | G |  | -0.210 | 0.045 | 2.9E-06 | 1.7E-02 | 20.2 | 0.004 | 0.018 | 0.812 |
| Executive function | Periodontitis | rs34393106 | A | C |  | 0.280 | 0.061 | 4.4E-06 | 3.8E-02 | 45.4 | -0.024 | 0.028 | 0.389 |
| Executive function | Periodontitis | rs6545794 | A | G |  | -0.330 | 0.071 | 2.9E-06 | 1.6E-02 | 18.6 | 0.028 | 0.027 | 0.312 |
| Visuospatial skill | Periodontitis | rs2075199 | T | C |  | -0.260 | 0.057 | 4.3E-06 | 2.2E-02 | 25.3 | -0.024 | 0.021 | 0.257 |
| Visuospatial skill | Periodontitis | rs57169846 | A | G |  | 0.220 | 0.047 | 2.8E-06 | 1.9E-02 | 22.2 | -0.013 | 0.018 | 0.478 |
| Attention/processing speed | Periodontitis | rs1403649 | C | T |  | -0.210 | 0.043 | 8.7E-07 | 2.0E-02 | 23.1 | 0.010 | 0.016 | 0.563 |
| Attention/processing speed | Periodontitis | rs17532412 | A | C |  | 0.280 | 0.051 | 3.4E-08 | 2.2E-02 | 25.9 | -0.024 | 0.022 | 0.274 |
| Attention/processing speed | Periodontitis | rs658424 | C | T |  | -0.260 | 0.057 | 4.4E-06 | 1.6E-02 | 18.9 | -0.029 | 0.021 | 0.171 |
| Attention/processing speed | Periodontitis | rs66671632 | T | C |  | -0.280 | 0.059 | 2.5E-06 | 1.7E-02 | 19.2 | -0.017 | 0.024 | 0.483 |
| Attention/processing speed | Periodontitis | rs7332702 | C | T |  | -0.240 | 0.051 | 2.7E-06 | 1.8E-02 | 20.6 | -0.010 | 0.019 | 0.580 |
| Memory | Periodontitis | rs2295752 | A | C |  | -0.230 | 0.050 | 4.3E-06 | 1.9E-02 | 21.8 | -0.042 | 0.017 | 0.012 |
| Memory | Periodontitis | rs2897044 | A | C |  | 0.240 | 0.050 | 1.6E-06 | 1.9E-02 | 22.3 | -0.033 | 0.020 | 0.104 |
| Memory | Periodontitis | rs3991624 | G | A |  | 0.290 | 0.059 | 7.4E-07 | 2.0E-02 | 23.6 | -0.418 | 0.188 | 0.027 |
|  |  |  |  |  |  |  |  |  |  |  |  |  |  |
|  |  |  |  |  |  |  |  |  |  |  |  |  |  |
|  |  |  |  |  |  |  |  |  |  |  |  |  |  |
|  |  |  |  |  |  |  |  |  |  |  |  |  |  |
|  |  |  |  |  |  |  |  |  |  |  |  |  |  |
|  |  |  |  |  |  |  |  |  |  |  |  |  |  |
|  |  |  |  |  |  |  |  |  |  |  |  |  |  |
|  |  |  |  |  |  |  |  |  |  |  |  |  |  |
|  |  |  |  |  |  |  |  |  |  |  |  |  |  |
| **Replication Cohort (FinnGen)** | | | | | | | | | | | | | |
| Periodontitis | Cortical Surface Area | rs10268587 | G | A | 0.037 | -0.295 | 0.062 | 2.1E-06 | 6.2E-03 | 1635.9 | 284.783 | 272.445 | 0.296 |
| Periodontitis | Cortical Surface Area | rs115120340 | T | C | 0.092 | 0.185 | 0.036 | 3.0E-07 | 5.7E-03 | 1511.1 | -44.627 | 421.383 | 0.916 |
| Periodontitis | Cortical Surface Area | rs11605185 | C | T | 0.061 | -0.231 | 0.047 | 1.1E-06 | 6.1E-03 | 1629.5 | -361.080 | 195.250 | 0.064 |
| Periodontitis | Cortical Surface Area | rs1241497 | A | G | 0.817 | -0.128 | 0.028 | 3.6E-06 | 4.9E-03 | 1288.8 | -158.012 | 158.541 | 0.319 |
| Periodontitis | Cortical Surface Area | rs130985 | T | C | 0.030 | -0.313 | 0.068 | 4.8E-06 | 5.7E-03 | 1499.1 | 236.010 | 264.888 | 0.373 |
| Periodontitis | Cortical Surface Area | rs139232605 | G | A | 0.062 | 0.221 | 0.042 | 1.1E-07 | 5.7E-03 | 1506.1 | 554.235 | 354.563 | 0.118 |
| Periodontitis | Cortical Surface Area | rs141098993 | A | G | 0.032 | 0.269 | 0.057 | 2.2E-06 | 4.4E-03 | 1168.1 | -82.965 | 390.845 | 0.832 |
| Periodontitis | Cortical Surface Area | rs146734691 | A | G | 0.003 | 0.733 | 0.160 | 4.8E-06 | 3.0E-03 | 784.2 | 562.895 | 637.298 | 0.377 |
| Periodontitis | Cortical Surface Area | rs2847728 | T | G | 0.211 | -0.126 | 0.027 | 3.3E-06 | 5.3E-03 | 1398.7 | 46.060 | 147.937 | 0.756 |
| Periodontitis | Cortical Surface Area | rs35813112 | G | A | 0.043 | 0.236 | 0.049 | 1.8E-06 | 4.6E-03 | 1209.6 | -336.774 | 272.948 | 0.217 |
| Periodontitis | Cortical Surface Area | rs55875437 | T | C | 0.203 | 0.126 | 0.026 | 1.5E-06 | 5.1E-03 | 1354.2 | 57.897 | 140.847 | 0.681 |
| Periodontitis | Cortical Surface Area | rs56265851 | T | A | 0.044 | 0.236 | 0.049 | 1.6E-06 | 4.7E-03 | 1250.1 | -56.422 | 440.007 | 0.898 |
| Periodontitis | Cortical Surface Area | rs6845106 | T | C | 0.593 | -0.102 | 0.022 | 3.0E-06 | 5.0E-03 | 1322.2 | -30.680 | 110.591 | 0.782 |
| Periodontitis | Cortical Surface Area | rs72682016 | A | T | 0.066 | -0.217 | 0.046 | 2.9E-06 | 5.8E-03 | 1547.0 | 78.564 | 589.617 | 0.894 |
| Periodontitis | Cortical Surface Area | rs7629105 | A | G | 0.933 | -0.189 | 0.041 | 3.3E-06 | 4.5E-03 | 1179.0 | -253.248 | 156.956 | 0.107 |
| Periodontitis | Cortical Surface Area | rs9645299 | C | T | 0.713 | 0.110 | 0.024 | 4.9E-06 | 5.0E-03 | 1320.8 | -78.037 | 122.707 | 0.525 |
| Periodontitis | Cortical Thickness | rs10268587 | G | A | 0.037 | -0.295 | 0.062 | 2.1E-06 | 6.2E-03 | 1635.9 | -0.002 | 0.002 | 0.226 |
| Periodontitis | Cortical Thickness | rs113059383 | G | A | 0.006 | -0.840 | 0.180 | 2.9E-06 | 8.5E-03 | 2267.0 | -0.004 | 0.004 | 0.231 |
| Periodontitis | Cortical Thickness | rs1148464 | T | C | 0.866 | -0.143 | 0.031 | 3.3E-06 | 4.7E-03 | 1253.9 | -0.001 | 0.002 | 0.792 |
| Periodontitis | Cortical Thickness | rs115120340 | T | C | 0.092 | 0.185 | 0.036 | 3.0E-07 | 5.7E-03 | 1511.1 | -0.006 | 0.003 | 0.049 |
| Periodontitis | Cortical Thickness | rs11605185 | C | T | 0.061 | -0.231 | 0.047 | 1.1E-06 | 6.1E-03 | 1629.5 | 0.000 | 0.001 | 0.922 |
| Periodontitis | Cortical Thickness | rs1241497 | A | G | 0.817 | -0.128 | 0.028 | 3.6E-06 | 4.9E-03 | 1288.8 | 0.000 | 0.001 | 0.822 |
| Periodontitis | Cortical Thickness | rs130985 | T | C | 0.030 | -0.313 | 0.068 | 4.8E-06 | 5.7E-03 | 1499.1 | -0.001 | 0.002 | 0.758 |
| Periodontitis | Cortical Thickness | rs139232605 | G | A | 0.062 | 0.221 | 0.042 | 1.1E-07 | 5.7E-03 | 1506.1 | -0.001 | 0.002 | 0.616 |
| Periodontitis | Cortical Thickness | rs141098993 | A | G | 0.032 | 0.269 | 0.057 | 2.2E-06 | 4.4E-03 | 1168.1 | 0.003 | 0.003 | 0.338 |
| Periodontitis | Cortical Thickness | rs146734691 | A | G | 0.003 | 0.733 | 0.160 | 4.8E-06 | 3.0E-03 | 784.2 | -0.001 | 0.004 | 0.757 |
| Periodontitis | Cortical Thickness | rs2847728 | T | G | 0.211 | -0.126 | 0.027 | 3.3E-06 | 5.3E-03 | 1398.7 | 0.001 | 0.001 | 0.564 |
| Periodontitis | Cortical Thickness | rs35813112 | G | A | 0.043 | 0.236 | 0.049 | 1.8E-06 | 4.6E-03 | 1209.6 | -0.002 | 0.002 | 0.326 |
| Periodontitis | Cortical Thickness | rs55875437 | T | C | 0.203 | 0.126 | 0.026 | 1.5E-06 | 5.1E-03 | 1354.2 | -0.001 | 0.001 | 0.111 |
| Periodontitis | Cortical Thickness | rs56265851 | T | A | 0.044 | 0.236 | 0.049 | 1.6E-06 | 4.7E-03 | 1250.1 | -0.004 | 0.003 | 0.191 |
| Periodontitis | Cortical Thickness | rs6845106 | T | C | 0.593 | -0.102 | 0.022 | 3.0E-06 | 5.0E-03 | 1322.2 | 0.000 | 0.001 | 0.708 |
| Periodontitis | Cortical Thickness | rs72682016 | A | T | 0.066 | -0.217 | 0.046 | 2.9E-06 | 5.8E-03 | 1547.0 | 0.004 | 0.004 | 0.295 |
| Periodontitis | Cortical Thickness | rs7629105 | A | G | 0.933 | -0.189 | 0.041 | 3.3E-06 | 4.5E-03 | 1179.0 | 0.002 | 0.001 | 0.020 |
| Periodontitis | Cortical Thickness | rs9645299 | C | T | 0.713 | 0.110 | 0.024 | 4.9E-06 | 5.0E-03 | 1320.8 | 0.000 | 0.001 | 0.641 |
| Periodontitis | Right Hippocampal volume | rs10268587 | G | A | 0.037 | -0.295 | 0.062 | 2.1E-06 | 6.2E-03 | 1635.9 | -3.292 | 6.623 | 0.619 |
| Periodontitis | Right Hippocampal volume | rs1148464 | T | C | 0.866 | -0.143 | 0.031 | 3.3E-06 | 4.7E-03 | 1253.9 | -5.235 | 5.009 | 0.296 |
| Periodontitis | Right Hippocampal volume | rs115120340 | T | C | 0.092 | 0.185 | 0.036 | 3.0E-07 | 5.7E-03 | 1511.1 | -4.182 | 9.092 | 0.646 |
| Periodontitis | Right Hippocampal volume | rs11605185 | C | T | 0.061 | -0.231 | 0.047 | 1.1E-06 | 6.1E-03 | 1629.5 | 0.895 | 4.405 | 0.839 |
| Periodontitis | Right Hippocampal volume | rs1241497 | A | G | 0.817 | -0.128 | 0.028 | 3.6E-06 | 4.9E-03 | 1288.8 | -6.504 | 3.988 | 0.103 |
| Periodontitis | Right Hippocampal volume | rs130985 | T | C | 0.030 | -0.313 | 0.068 | 4.8E-06 | 5.7E-03 | 1499.1 | 4.666 | 5.800 | 0.421 |
| Periodontitis | Right Hippocampal volume | rs139232605 | G | A | 0.062 | 0.221 | 0.042 | 1.1E-07 | 5.7E-03 | 1506.1 | -6.084 | 8.881 | 0.493 |
| Periodontitis | Right Hippocampal volume | rs141098993 | A | G | 0.032 | 0.269 | 0.057 | 2.2E-06 | 4.4E-03 | 1168.1 | -9.866 | 9.033 | 0.275 |
| Periodontitis | Right Hippocampal volume | rs2847728 | T | G | 0.211 | -0.126 | 0.027 | 3.3E-06 | 5.3E-03 | 1398.7 | 3.204 | 3.460 | 0.354 |
| Periodontitis | Right Hippocampal volume | rs35813112 | G | A | 0.043 | 0.236 | 0.049 | 1.8E-06 | 4.6E-03 | 1209.6 | -15.386 | 6.420 | 0.017 |
| Periodontitis | Right Hippocampal volume | rs55875437 | T | C | 0.203 | 0.126 | 0.026 | 1.5E-06 | 5.1E-03 | 1354.2 | 0.160 | 3.487 | 0.963 |
| Periodontitis | Right Hippocampal volume | rs56265851 | T | A | 0.044 | 0.236 | 0.049 | 1.6E-06 | 4.7E-03 | 1250.1 | 17.557 | 10.494 | 0.094 |
| Periodontitis | Right Hippocampal volume | rs6845106 | T | C | 0.593 | -0.102 | 0.022 | 3.0E-06 | 5.0E-03 | 1322.2 | 0.599 | 2.766 | 0.829 |
| Periodontitis | Right Hippocampal volume | rs7629105 | A | G | 0.933 | -0.189 | 0.041 | 3.3E-06 | 4.5E-03 | 1179.0 | -1.744 | 3.887 | 0.654 |
| Periodontitis | Right Hippocampal volume | rs9645299 | C | T | 0.713 | 0.110 | 0.024 | 4.9E-06 | 5.0E-03 | 1320.8 | -4.459 | 3.083 | 0.148 |
| Periodontitis | Left Hippocampal volume | rs10268587 | G | A | 0.037 | -0.295 | 0.062 | 2.1E-06 | 6.2E-03 | 1635.9 | -1.236 | 6.241 | 0.843 |
| Periodontitis | Left Hippocampal volume | rs1148464 | T | C | 0.866 | -0.143 | 0.031 | 3.3E-06 | 4.7E-03 | 1253.9 | -3.501 | 4.728 | 0.459 |
| Periodontitis | Left Hippocampal volume | rs115120340 | T | C | 0.092 | 0.185 | 0.036 | 3.0E-07 | 5.7E-03 | 1511.1 | -11.861 | 8.575 | 0.167 |
| Periodontitis | Left Hippocampal volume | rs11605185 | C | T | 0.061 | -0.231 | 0.047 | 1.1E-06 | 6.1E-03 | 1629.5 | 0.598 | 4.153 | 0.885 |
| Periodontitis | Left Hippocampal volume | rs1241497 | A | G | 0.817 | -0.128 | 0.028 | 3.6E-06 | 4.9E-03 | 1288.8 | -2.704 | 3.762 | 0.472 |
| Periodontitis | Left Hippocampal volume | rs130985 | T | C | 0.030 | -0.313 | 0.068 | 4.8E-06 | 5.7E-03 | 1499.1 | 4.875 | 5.470 | 0.373 |
| Periodontitis | Left Hippocampal volume | rs139232605 | G | A | 0.062 | 0.221 | 0.042 | 1.1E-07 | 5.7E-03 | 1506.1 | -3.273 | 8.371 | 0.696 |
| Periodontitis | Left Hippocampal volume | rs141098993 | A | G | 0.032 | 0.269 | 0.057 | 2.2E-06 | 4.4E-03 | 1168.1 | -7.939 | 8.512 | 0.351 |
| Periodontitis | Left Hippocampal volume | rs2847728 | T | G | 0.211 | -0.126 | 0.027 | 3.3E-06 | 5.3E-03 | 1398.7 | 4.216 | 3.263 | 0.196 |
| Periodontitis | Left Hippocampal volume | rs35813112 | G | A | 0.043 | 0.236 | 0.049 | 1.8E-06 | 4.6E-03 | 1209.6 | -11.524 | 6.055 | 0.057 |
| Periodontitis | Left Hippocampal volume | rs55875437 | T | C | 0.203 | 0.126 | 0.026 | 1.5E-06 | 5.1E-03 | 1354.2 | 1.234 | 3.289 | 0.707 |
| Periodontitis | Left Hippocampal volume | rs56265851 | T | A | 0.044 | 0.236 | 0.049 | 1.6E-06 | 4.7E-03 | 1250.1 | 8.767 | 9.898 | 0.376 |
| Periodontitis | Left Hippocampal volume | rs6845106 | T | C | 0.593 | -0.102 | 0.022 | 3.0E-06 | 5.0E-03 | 1322.2 | -0.741 | 2.609 | 0.776 |
| Periodontitis | Left Hippocampal volume | rs7629105 | A | G | 0.933 | -0.189 | 0.041 | 3.3E-06 | 4.5E-03 | 1179.0 | -2.623 | 3.664 | 0.474 |
| Periodontitis | Left Hippocampal volume | rs9645299 | C | T | 0.713 | 0.110 | 0.024 | 4.9E-06 | 5.0E-03 | 1320.8 | -2.784 | 2.909 | 0.339 |
| Periodontitis | Cognitive Performance | rs10268587 | G | A | 0.037 | -0.295 | 0.062 | 2.1E-06 | 6.2E-03 | 1635.9 | 0.004 | 0.007 | 0.551 |
| Periodontitis | Cognitive Performance | rs1148464 | T | C | 0.866 | -0.143 | 0.031 | 3.3E-06 | 4.7E-03 | 1253.9 | -0.010 | 0.005 | 0.059 |
| Periodontitis | Cognitive Performance | rs115120340 | T | C | 0.092 | 0.185 | 0.036 | 3.0E-07 | 5.7E-03 | 1511.1 | -0.022 | 0.009 | 0.013 |
| Periodontitis | Cognitive Performance | rs1241497 | A | G | 0.817 | -0.128 | 0.028 | 3.6E-06 | 4.9E-03 | 1288.8 | 0.008 | 0.004 | 0.060 |
| Periodontitis | Cognitive Performance | rs130985 | T | C | 0.030 | -0.313 | 0.068 | 4.8E-06 | 5.7E-03 | 1499.1 | 0.002 | 0.006 | 0.765 |
| Periodontitis | Cognitive Performance | rs139232605 | G | A | 0.062 | 0.221 | 0.042 | 1.1E-07 | 5.7E-03 | 1506.1 | -0.009 | 0.009 | 0.288 |
| Periodontitis | Cognitive Performance | rs141098993 | A | G | 0.032 | 0.269 | 0.057 | 2.2E-06 | 4.4E-03 | 1168.1 | -0.005 | 0.009 | 0.585 |
| Periodontitis | Cognitive Performance | rs146734691 | A | G | 0.003 | 0.733 | 0.160 | 4.8E-06 | 3.0E-03 | 784.2 | -0.009 | 0.015 | 0.567 |
| Periodontitis | Cognitive Performance | rs2847728 | T | G | 0.211 | -0.126 | 0.027 | 3.3E-06 | 5.3E-03 | 1398.7 | 0.001 | 0.004 | 0.789 |
| Periodontitis | Cognitive Performance | rs55875437 | T | C | 0.203 | 0.126 | 0.026 | 1.5E-06 | 5.1E-03 | 1354.2 | 0.003 | 0.004 | 0.431 |
| Periodontitis | Cognitive Performance | rs56265851 | T | A | 0.044 | 0.236 | 0.049 | 1.6E-06 | 4.7E-03 | 1250.1 | -0.018 | 0.011 | 0.091 |
| Periodontitis | Cognitive Performance | rs6845106 | T | C | 0.593 | -0.102 | 0.022 | 3.0E-06 | 5.0E-03 | 1322.2 | -0.006 | 0.003 | 0.053 |
| Periodontitis | Cognitive Performance | rs72682016 | A | T | 0.066 | -0.217 | 0.046 | 2.9E-06 | 5.8E-03 | 1547.0 | -0.007 | 0.018 | 0.705 |
| Periodontitis | Cognitive Performance | rs7629105 | A | G | 0.933 | -0.189 | 0.041 | 3.3E-06 | 4.5E-03 | 1179.0 | -0.002 | 0.004 | 0.666 |
| Periodontitis | Fluid Intelligence Score | rs10268587 | G | A | 0.037 | -0.295 | 0.062 | 2.1E-06 | 6.2E-03 | 1635.9 | 0.015 | 0.018 | 0.420 |
| Periodontitis | Fluid Intelligence Score | rs113059383 | G | A | 0.006 | -0.840 | 0.180 | 2.9E-06 | 8.5E-03 | 2267.0 | -0.002 | 0.029 | 0.930 |
| Periodontitis | Fluid Intelligence Score | rs1148464 | T | C | 0.866 | -0.143 | 0.031 | 3.3E-06 | 4.7E-03 | 1253.9 | -0.026 | 0.014 | 0.061 |
| Periodontitis | Fluid Intelligence Score | rs115120340 | T | C | 0.092 | 0.185 | 0.036 | 3.0E-07 | 5.7E-03 | 1511.1 | -0.059 | 0.024 | 0.016 |
| Periodontitis | Fluid Intelligence Score | rs11605185 | C | T | 0.061 | -0.231 | 0.047 | 1.1E-06 | 6.1E-03 | 1629.5 | 0.017 | 0.012 | 0.170 |
| Periodontitis | Fluid Intelligence Score | rs1241497 | A | G | 0.817 | -0.128 | 0.028 | 3.6E-06 | 4.9E-03 | 1288.8 | 0.021 | 0.011 | 0.054 |
| Periodontitis | Fluid Intelligence Score | rs130985 | T | C | 0.030 | -0.313 | 0.068 | 4.8E-06 | 5.7E-03 | 1499.1 | -0.001 | 0.016 | 0.960 |
| Periodontitis | Fluid Intelligence Score | rs139232605 | G | A | 0.062 | 0.221 | 0.042 | 1.1E-07 | 5.7E-03 | 1506.1 | -0.012 | 0.025 | 0.630 |
| Periodontitis | Fluid Intelligence Score | rs141098993 | A | G | 0.032 | 0.269 | 0.057 | 2.2E-06 | 4.4E-03 | 1168.1 | -0.018 | 0.025 | 0.480 |
| Periodontitis | Fluid Intelligence Score | rs146734691 | A | G | 0.003 | 0.733 | 0.160 | 4.8E-06 | 3.0E-03 | 784.2 | -0.017 | 0.042 | 0.680 |
| Periodontitis | Fluid Intelligence Score | rs2847728 | T | G | 0.211 | -0.126 | 0.027 | 3.3E-06 | 5.3E-03 | 1398.7 | 0.005 | 0.010 | 0.580 |
| Periodontitis | Fluid Intelligence Score | rs55875437 | T | C | 0.203 | 0.126 | 0.026 | 1.5E-06 | 5.1E-03 | 1354.2 | 0.015 | 0.010 | 0.110 |
| Periodontitis | Fluid Intelligence Score | rs56265851 | T | A | 0.044 | 0.236 | 0.049 | 1.6E-06 | 4.7E-03 | 1250.1 | -0.039 | 0.029 | 0.190 |
| Periodontitis | Fluid Intelligence Score | rs6845106 | T | C | 0.593 | -0.102 | 0.022 | 3.0E-06 | 5.0E-03 | 1322.2 | -0.006 | 0.008 | 0.470 |
| Periodontitis | Fluid Intelligence Score | rs7629105 | A | G | 0.933 | -0.189 | 0.041 | 3.3E-06 | 4.5E-03 | 1179.0 | 0.001 | 0.011 | 0.910 |
| Periodontitis | Prospective Memory | rs10268587 | G | A | 0.037 | -0.295 | 0.062 | 2.1E-06 | 6.2E-03 | 1635.9 | -0.007 | 0.005 | 0.150 |
| Periodontitis | Prospective Memory | rs113059383 | G | A | 0.006 | -0.840 | 0.180 | 2.9E-06 | 8.5E-03 | 2267.0 | -0.012 | 0.007 | 0.095 |
| Periodontitis | Prospective Memory | rs1148464 | T | C | 0.866 | -0.143 | 0.031 | 3.3E-06 | 4.7E-03 | 1253.9 | 0.002 | 0.003 | 0.650 |
| Periodontitis | Prospective Memory | rs115120340 | T | C | 0.092 | 0.185 | 0.036 | 3.0E-07 | 5.7E-03 | 1511.1 | 0.000 | 0.006 | 0.980 |
| Periodontitis | Prospective Memory | rs11605185 | C | T | 0.061 | -0.231 | 0.047 | 1.1E-06 | 6.1E-03 | 1629.5 | -0.002 | 0.003 | 0.570 |
| Periodontitis | Prospective Memory | rs1241497 | A | G | 0.817 | -0.128 | 0.028 | 3.6E-06 | 4.9E-03 | 1288.8 | -0.006 | 0.003 | 0.033 |
| Periodontitis | Prospective Memory | rs130985 | T | C | 0.030 | -0.313 | 0.068 | 4.8E-06 | 5.7E-03 | 1499.1 | 0.006 | 0.004 | 0.120 |
| Periodontitis | Prospective Memory | rs139232605 | G | A | 0.062 | 0.221 | 0.042 | 1.1E-07 | 5.7E-03 | 1506.1 | -0.002 | 0.006 | 0.760 |
| Periodontitis | Prospective Memory | rs141098993 | A | G | 0.032 | 0.269 | 0.057 | 2.2E-06 | 4.4E-03 | 1168.1 | -0.002 | 0.006 | 0.750 |
| Periodontitis | Prospective Memory | rs146734691 | A | G | 0.003 | 0.733 | 0.160 | 4.8E-06 | 3.0E-03 | 784.2 | -0.010 | 0.010 | 0.350 |
| Periodontitis | Prospective Memory | rs2847728 | T | G | 0.211 | -0.126 | 0.027 | 3.3E-06 | 5.3E-03 | 1398.7 | -0.001 | 0.002 | 0.820 |
| Periodontitis | Prospective Memory | rs35813112 | G | A | 0.043 | 0.236 | 0.049 | 1.8E-06 | 4.6E-03 | 1209.6 | 0.006 | 0.004 | 0.190 |
| Periodontitis | Prospective Memory | rs55875437 | T | C | 0.203 | 0.126 | 0.026 | 1.5E-06 | 5.1E-03 | 1354.2 | -0.002 | 0.002 | 0.420 |
| Periodontitis | Prospective Memory | rs56265851 | T | A | 0.044 | 0.236 | 0.049 | 1.6E-06 | 4.7E-03 | 1250.1 | 0.000 | 0.007 | 0.990 |
| Periodontitis | Prospective Memory | rs6845106 | T | C | 0.593 | -0.102 | 0.022 | 3.0E-06 | 5.0E-03 | 1322.2 | -0.001 | 0.002 | 0.450 |
| Periodontitis | Prospective Memory | rs7629105 | A | G | 0.933 | -0.189 | 0.041 | 3.3E-06 | 4.5E-03 | 1179.0 | 0.001 | 0.003 | 0.800 |
| Periodontitis | Prospective Memory | rs9645299 | C | T | 0.713 | 0.110 | 0.024 | 4.9E-06 | 5.0E-03 | 1320.8 | -0.002 | 0.002 | 0.420 |
| Periodontitis | Reaction Time | rs10268587 | G | A | 0.037 | -0.295 | 0.062 | 2.1E-06 | 6.2E-03 | 1635.9 | -0.001 | 0.002 | 0.640 |
| Periodontitis | Reaction Time | rs1148464 | T | C | 0.866 | -0.143 | 0.031 | 3.3E-06 | 4.7E-03 | 1253.9 | 0.004 | 0.002 | 0.039 |
| Periodontitis | Reaction Time | rs115120340 | T | C | 0.092 | 0.185 | 0.036 | 3.0E-07 | 5.7E-03 | 1511.1 | -0.003 | 0.002 | 0.136 |
| Periodontitis | Reaction Time | rs11605185 | C | T | 0.061 | -0.231 | 0.047 | 1.1E-06 | 6.1E-03 | 1629.5 | -0.005 | 0.002 | 0.005 |
| Periodontitis | Reaction Time | rs1241497 | A | G | 0.817 | -0.128 | 0.028 | 3.6E-06 | 4.9E-03 | 1288.8 | 0.001 | 0.002 | 0.728 |
| Periodontitis | Reaction Time | rs130985 | T | C | 0.030 | -0.313 | 0.068 | 4.8E-06 | 5.7E-03 | 1499.1 | -0.002 | 0.002 | 0.229 |
| Periodontitis | Reaction Time | rs139232605 | G | A | 0.062 | 0.221 | 0.042 | 1.1E-07 | 5.7E-03 | 1506.1 | 0.002 | 0.002 | 0.260 |
| Periodontitis | Reaction Time | rs141098993 | A | G | 0.032 | 0.269 | 0.057 | 2.2E-06 | 4.4E-03 | 1168.1 | -0.001 | 0.002 | 0.610 |
| Periodontitis | Reaction Time | rs2847728 | T | G | 0.211 | -0.126 | 0.027 | 3.3E-06 | 5.3E-03 | 1398.7 | 0.000 | 0.002 | 0.854 |
| Periodontitis | Reaction Time | rs35813112 | G | A | 0.043 | 0.236 | 0.049 | 1.8E-06 | 4.6E-03 | 1209.6 | 0.005 | 0.002 | 0.002 |
| Periodontitis | Reaction Time | rs55875437 | T | C | 0.203 | 0.126 | 0.026 | 1.5E-06 | 5.1E-03 | 1354.2 | 0.000 | 0.002 | 0.961 |
| Periodontitis | Reaction Time | rs6845106 | T | C | 0.593 | -0.102 | 0.022 | 3.0E-06 | 5.0E-03 | 1322.2 | -0.001 | 0.002 | 0.733 |
| Periodontitis | Reaction Time | rs7629105 | A | G | 0.933 | -0.189 | 0.041 | 3.3E-06 | 4.5E-03 | 1179.0 | -0.002 | 0.002 | 0.329 |
| Periodontitis | Reaction Time | rs9645299 | C | T | 0.713 | 0.110 | 0.024 | 4.9E-06 | 5.0E-03 | 1320.8 | 0.005 | 0.002 | 0.010 |
| Periodontitis | Alzheimer's disease | rs10268587 | G | A | 0.037 | -0.295 | 0.062 | 2.1E-06 | 6.2E-03 | 1635.9 | 0.037 | 0.019 | 0.057 |
| Periodontitis | Alzheimer's disease | rs113059383 | G | A | 0.006 | -0.840 | 0.180 | 2.9E-06 | 8.5E-03 | 2267.0 | 0.022 | 0.031 | 0.469 |
| Periodontitis | Alzheimer's disease | rs1148464 | T | C | 0.866 | -0.143 | 0.031 | 3.3E-06 | 4.7E-03 | 1253.9 | 0.003 | 0.016 | 0.873 |
| Periodontitis | Alzheimer's disease | rs115120340 | T | C | 0.092 | 0.185 | 0.036 | 3.0E-07 | 5.7E-03 | 1511.1 | 0.029 | 0.025 | 0.240 |
| Periodontitis | Alzheimer's disease | rs11605185 | C | T | 0.061 | -0.231 | 0.047 | 1.1E-06 | 6.1E-03 | 1629.5 | 0.010 | 0.014 | 0.495 |
| Periodontitis | Alzheimer's disease | rs1241497 | A | G | 0.817 | -0.128 | 0.028 | 3.6E-06 | 4.9E-03 | 1288.8 | 0.022 | 0.012 | 0.066 |
| Periodontitis | Alzheimer's disease | rs130985 | T | C | 0.030 | -0.313 | 0.068 | 4.8E-06 | 5.7E-03 | 1499.1 | -0.011 | 0.017 | 0.524 |
| Periodontitis | Alzheimer's disease | rs139232605 | G | A | 0.062 | 0.221 | 0.042 | 1.1E-07 | 5.7E-03 | 1506.1 | -0.012 | 0.026 | 0.627 |
| Periodontitis | Alzheimer's disease | rs141098993 | A | G | 0.032 | 0.269 | 0.057 | 2.2E-06 | 4.4E-03 | 1168.1 | -0.025 | 0.028 | 0.367 |
| Periodontitis | Alzheimer's disease | rs146734691 | A | G | 0.003 | 0.733 | 0.160 | 4.8E-06 | 3.0E-03 | 784.2 | -0.031 | 0.043 | 0.478 |
| Periodontitis | Alzheimer's disease | rs2847728 | T | G | 0.211 | -0.126 | 0.027 | 3.3E-06 | 5.3E-03 | 1398.7 | 0.031 | 0.011 | 0.004 |
| Periodontitis | Alzheimer's disease | rs35813112 | G | A | 0.043 | 0.236 | 0.049 | 1.8E-06 | 4.6E-03 | 1209.6 | 0.013 | 0.020 | 0.509 |
| Periodontitis | Alzheimer's disease | rs55875437 | T | C | 0.203 | 0.126 | 0.026 | 1.5E-06 | 5.1E-03 | 1354.2 | 0.006 | 0.010 | 0.581 |
| Periodontitis | Alzheimer's disease | rs56265851 | T | A | 0.044 | 0.236 | 0.049 | 1.6E-06 | 4.7E-03 | 1250.1 | -0.011 | 0.032 | 0.722 |
| Periodontitis | Alzheimer's disease | rs6845106 | T | C | 0.593 | -0.102 | 0.022 | 3.0E-06 | 5.0E-03 | 1322.2 | -0.003 | 0.008 | 0.680 |
| Periodontitis | Alzheimer's disease | rs72682016 | A | T | 0.066 | -0.217 | 0.046 | 2.9E-06 | 5.8E-03 | 1547.0 | -0.011 | 0.052 | 0.836 |
| Periodontitis | Alzheimer's disease | rs7629105 | A | G | 0.933 | -0.189 | 0.041 | 3.3E-06 | 4.5E-03 | 1179.0 | -0.014 | 0.012 | 0.230 |
| Periodontitis | Alzheimer's disease | rs9645299 | C | T | 0.713 | 0.110 | 0.024 | 4.9E-06 | 5.0E-03 | 1320.8 | 0.012 | 0.009 | 0.186 |
| Periodontitis | Lewy body dementia | rs10268587 | G | A | 0.037 | -0.295 | 0.062 | 2.1E-06 | 6.2E-03 | 1635.9 | 0.037 | 0.088 | 0.673 |
| Periodontitis | Lewy body dementia | rs1148464 | T | C | 0.866 | -0.143 | 0.031 | 3.3E-06 | 4.7E-03 | 1253.9 | 0.023 | 0.066 | 0.722 |
| Periodontitis | Lewy body dementia | rs115120340 | T | C | 0.092 | 0.185 | 0.036 | 3.0E-07 | 5.7E-03 | 1511.1 | 0.007 | 0.115 | 0.951 |
| Periodontitis | Lewy body dementia | rs11605185 | C | T | 0.061 | -0.231 | 0.047 | 1.1E-06 | 6.1E-03 | 1629.5 | -0.034 | 0.064 | 0.589 |
| Periodontitis | Lewy body dementia | rs1241497 | A | G | 0.817 | -0.128 | 0.028 | 3.6E-06 | 4.9E-03 | 1288.8 | -0.033 | 0.054 | 0.537 |
| Periodontitis | Lewy body dementia | rs130985 | T | C | 0.030 | -0.313 | 0.068 | 4.8E-06 | 5.7E-03 | 1499.1 | -0.023 | 0.077 | 0.765 |
| Periodontitis | Lewy body dementia | rs139232605 | G | A | 0.062 | 0.221 | 0.042 | 1.1E-07 | 5.7E-03 | 1506.1 | -0.192 | 0.122 | 0.117 |
| Periodontitis | Lewy body dementia | rs141098993 | A | G | 0.032 | 0.269 | 0.057 | 2.2E-06 | 4.4E-03 | 1168.1 | 0.063 | 0.115 | 0.583 |
| Periodontitis | Lewy body dementia | rs2847728 | T | G | 0.211 | -0.126 | 0.027 | 3.3E-06 | 5.3E-03 | 1398.7 | -0.013 | 0.047 | 0.792 |
| Periodontitis | Lewy body dementia | rs35813112 | G | A | 0.043 | 0.236 | 0.049 | 1.8E-06 | 4.6E-03 | 1209.6 | 0.047 | 0.091 | 0.604 |
| Periodontitis | Lewy body dementia | rs55875437 | T | C | 0.203 | 0.126 | 0.026 | 1.5E-06 | 5.1E-03 | 1354.2 | -0.038 | 0.047 | 0.414 |
| Periodontitis | Lewy body dementia | rs56265851 | T | A | 0.044 | 0.236 | 0.049 | 1.6E-06 | 4.7E-03 | 1250.1 | 0.162 | 0.136 | 0.233 |
| Periodontitis | Lewy body dementia | rs6845106 | T | C | 0.593 | -0.102 | 0.022 | 3.0E-06 | 5.0E-03 | 1322.2 | 0.013 | 0.038 | 0.729 |
| Periodontitis | Lewy body dementia | rs7629105 | A | G | 0.933 | -0.189 | 0.041 | 3.3E-06 | 4.5E-03 | 1179.0 | 0.007 | 0.051 | 0.897 |
| Periodontitis | Lewy body dementia | rs9645299 | C | T | 0.713 | 0.110 | 0.024 | 4.9E-06 | 5.0E-03 | 1320.8 | -0.010 | 0.042 | 0.804 |
| Periodontitis | Vascular Dementia | rs10268587 | G | A | 0.037 | -0.295 | 0.062 | 2.1E-06 | 6.2E-03 | 1635.9 | 0.048 | 0.163 | 0.768 |
| Periodontitis | Vascular Dementia | rs113059383 | G | A | 0.006 | -0.840 | 0.180 | 2.9E-06 | 8.5E-03 | 2267.0 | 0.184 | 0.262 | 0.483 |
| Periodontitis | Vascular Dementia | rs1148464 | T | C | 0.866 | -0.143 | 0.031 | 3.3E-06 | 4.7E-03 | 1253.9 | -0.025 | 0.125 | 0.843 |
| Periodontitis | Vascular Dementia | rs115120340 | T | C | 0.092 | 0.185 | 0.036 | 3.0E-07 | 5.7E-03 | 1511.1 | 0.304 | 0.221 | 0.169 |
| Periodontitis | Vascular Dementia | rs11605185 | C | T | 0.061 | -0.231 | 0.047 | 1.1E-06 | 6.1E-03 | 1629.5 | 0.036 | 0.110 | 0.741 |
| Periodontitis | Vascular Dementia | rs1241497 | A | G | 0.817 | -0.128 | 0.028 | 3.6E-06 | 4.9E-03 | 1288.8 | 0.066 | 0.099 | 0.507 |
| Periodontitis | Vascular Dementia | rs130985 | T | C | 0.030 | -0.313 | 0.068 | 4.8E-06 | 5.7E-03 | 1499.1 | 0.143 | 0.143 | 0.319 |
| Periodontitis | Vascular Dementia | rs139232605 | G | A | 0.062 | 0.221 | 0.042 | 1.1E-07 | 5.7E-03 | 1506.1 | -0.021 | 0.224 | 0.927 |
| Periodontitis | Vascular Dementia | rs141098993 | A | G | 0.032 | 0.269 | 0.057 | 2.2E-06 | 4.4E-03 | 1168.1 | -0.125 | 0.220 | 0.569 |
| Periodontitis | Vascular Dementia | rs146734691 | A | G | 0.003 | 0.733 | 0.160 | 4.8E-06 | 3.0E-03 | 784.2 | -0.132 | 0.384 | 0.730 |
| Periodontitis | Vascular Dementia | rs2847728 | T | G | 0.211 | -0.126 | 0.027 | 3.3E-06 | 5.3E-03 | 1398.7 | -0.029 | 0.085 | 0.730 |
| Periodontitis | Vascular Dementia | rs35813112 | G | A | 0.043 | 0.236 | 0.049 | 1.8E-06 | 4.6E-03 | 1209.6 | 0.290 | 0.159 | 0.069 |
| Periodontitis | Vascular Dementia | rs55875437 | T | C | 0.203 | 0.126 | 0.026 | 1.5E-06 | 5.1E-03 | 1354.2 | -0.069 | 0.086 | 0.424 |
| Periodontitis | Vascular Dementia | rs56265851 | T | A | 0.044 | 0.236 | 0.049 | 1.6E-06 | 4.7E-03 | 1250.1 | 0.319 | 0.264 | 0.227 |
| Periodontitis | Vascular Dementia | rs6845106 | T | C | 0.593 | -0.102 | 0.022 | 3.0E-06 | 5.0E-03 | 1322.2 | 0.149 | 0.069 | 0.030 |
| Periodontitis | Vascular Dementia | rs7629105 | A | G | 0.933 | -0.189 | 0.041 | 3.3E-06 | 4.5E-03 | 1179.0 | -0.005 | 0.096 | 0.961 |
| Periodontitis | Vascular Dementia | rs9645299 | C | T | 0.713 | 0.110 | 0.024 | 4.9E-06 | 5.0E-03 | 1320.8 | 0.086 | 0.077 | 0.261 |
|  |  |  |  |  |  |  |  |  |  |  |  |  |  |
|  |  |  |  |  |  |  |  |  |  |  |  |  |  |
| Cortical Surface Area | Periodontitis | rs10878349 | A | G | 0.483 | 1040.514 | 111.325 | 9.0E-21 | 2.6E-03 | 87.4 | 0.007 | 0.022 | 0.733 |
| Cortical Surface Area | Periodontitis | rs1628768 | T | C | 0.764 | -918.535 | 133.630 | 6.3E-12 | 1.4E-03 | 47.2 | 0.012 | 0.024 | 0.619 |
| Cortical Surface Area | Periodontitis | rs2301718 | A | G | 0.240 | 729.921 | 132.345 | 3.5E-08 | 9.1E-04 | 30.4 | 0.011 | 0.023 | 0.636 |
| Cortical Surface Area | Periodontitis | rs34464850 | C | G | 0.156 | 1246.357 | 152.955 | 3.7E-16 | 2.0E-03 | 66.4 | 0.014 | 0.029 | 0.624 |
| Cortical Surface Area | Periodontitis | rs62057153 | T | C | 0.780 | 1685.224 | 145.149 | 3.7E-31 | 4.4E-03 | 134.8 | 0.088 | 0.039 | 0.023 |
| Cortical Surface Area | Periodontitis | rs7715167 | T | C | 0.386 | -690.511 | 120.803 | 1.1E-08 | 9.9E-04 | 32.7 | -0.019 | 0.022 | 0.381 |
| Cortical Surface Area | Periodontitis | rs9832327 | T | C | 0.749 | -720.991 | 127.737 | 1.7E-08 | 9.5E-04 | 31.9 | -0.012 | 0.026 | 0.644 |
| Cortical Thickness | Periodontitis | rs11612673 | T | C | 0.532 | 0.004 | 0.001 | 1.9E-08 | 8.2E-04 | 28.9 | -0.028 | 0.022 | 0.200 |
| Cortical Thickness | Periodontitis | rs11692435 | A | G | 0.090 | -0.009 | 0.002 | 1.3E-09 | 1.2E-03 | 36.0 | 0.021 | 0.026 | 0.423 |
| Cortical Thickness | Periodontitis | rs13110077 | T | C | 0.235 | 0.005 | 0.001 | 1.9E-08 | 8.4E-04 | 29.6 | 0.022 | 0.029 | 0.444 |
| Cortical Thickness | Periodontitis | rs2316767 | T | C | 0.791 | -0.007 | 0.001 | 7.3E-10 | 1.4E-03 | 37.1 | 0.088 | 0.039 | 0.024 |
| Cortical Thickness | Periodontitis | rs3816046 | T | C | 0.321 | -0.004 | 0.001 | 2.0E-08 | 9.3E-04 | 30.3 | 0.002 | 0.024 | 0.941 |
| Cortical Thickness | Periodontitis | rs630934 | A | C | 0.496 | -0.005 | 0.001 | 1.2E-12 | 1.3E-03 | 45.6 | 0.008 | 0.021 | 0.724 |
| Cortical Thickness | Periodontitis | rs7824177 | A | G | 0.823 | 0.005 | 0.001 | 3.6E-08 | 9.5E-04 | 33.4 | -0.074 | 0.034 | 0.031 |
| Right Hippocampal volume | Periodontitis | rs11206344 | C | A | 0.501 | 17.917 | 2.710 | 3.8E-11 | 1.2E-03 | 43.7 | 0.024 | 0.022 | 0.271 |
| Right Hippocampal volume | Periodontitis | rs1423642 | A | C | 0.653 | -17.337 | 2.855 | 1.3E-09 | 1.0E-03 | 36.9 | 0.008 | 0.023 | 0.740 |
| Right Hippocampal volume | Periodontitis | rs146607495 | C | T | 0.903 | -62.616 | 4.655 | 3.0E-41 | 4.9E-03 | 180.9 | -0.012 | 0.040 | 0.774 |
| Right Hippocampal volume | Periodontitis | rs17178006 | T | G | 0.883 | 54.969 | 4.229 | 1.3E-38 | 4.6E-03 | 168.9 | -0.046 | 0.045 | 0.304 |
| Right Hippocampal volume | Periodontitis | rs1741948 | T | G | 0.389 | -15.246 | 2.778 | 4.1E-08 | 8.2E-04 | 30.1 | 0.001 | 0.022 | 0.963 |
| Right Hippocampal volume | Periodontitis | rs2578475 | T | G | 0.657 | 19.904 | 2.886 | 5.3E-12 | 1.3E-03 | 47.6 | 0.031 | 0.023 | 0.173 |
| Right Hippocampal volume | Periodontitis | rs28758826 | A | G | 0.451 | 16.080 | 2.744 | 4.6E-09 | 9.3E-04 | 34.4 | -0.015 | 0.022 | 0.482 |
| Right Hippocampal volume | Periodontitis | rs2970931 | T | C | 0.428 | 22.435 | 2.778 | 6.6E-16 | 1.8E-03 | 65.2 | -0.015 | 0.022 | 0.497 |
| Right Hippocampal volume | Periodontitis | rs309587 | C | T | 0.317 | 21.238 | 2.915 | 3.2E-13 | 1.4E-03 | 53.1 | -0.030 | 0.022 | 0.173 |
| Right Hippocampal volume | Periodontitis | rs33931638 | G | A | 0.926 | 30.496 | 5.185 | 4.1E-09 | 9.4E-04 | 34.6 | -0.032 | 0.041 | 0.435 |
| Right Hippocampal volume | Periodontitis | rs55905347 | G | A | 0.640 | 17.174 | 2.883 | 2.6E-09 | 9.6E-04 | 35.5 | 0.008 | 0.022 | 0.701 |
| Right Hippocampal volume | Periodontitis | rs56072903 | T | C | 0.798 | 28.850 | 3.492 | 1.4E-16 | 1.9E-03 | 68.3 | 0.090 | 0.039 | 0.021 |
| Right Hippocampal volume | Periodontitis | rs6791514 | C | G | 0.276 | 24.408 | 3.056 | 1.4E-15 | 1.7E-03 | 63.8 | -0.004 | 0.024 | 0.875 |
| Right Hippocampal volume | Periodontitis | rs7852872 | C | G | 0.633 | -25.024 | 2.810 | 5.4E-19 | 2.2E-03 | 79.3 | 0.010 | 0.022 | 0.631 |
| Right Hippocampal volume | Periodontitis | rs929387 | G | A | 0.683 | -16.545 | 2.939 | 1.8E-08 | 8.6E-04 | 31.7 | 0.003 | 0.023 | 0.908 |
| Right Hippocampal volume | Periodontitis | rs9322190 | G | A | 0.670 | -22.607 | 2.885 | 4.6E-15 | 1.7E-03 | 61.4 | -0.005 | 0.023 | 0.815 |
| Right Hippocampal volume | Periodontitis | rs9638084 | A | G | 0.398 | 17.772 | 2.776 | 1.5E-10 | 1.1E-03 | 41.0 | -0.003 | 0.022 | 0.891 |
| Left Hippocampal volume | Periodontitis | rs11206344 | C | A | 0.501 | 18.751 | 2.557 | 2.3E-13 | 1.5E-03 | 53.8 | 0.024 | 0.022 | 0.271 |
| Left Hippocampal volume | Periodontitis | rs1404220 | C | A | 0.350 | 17.477 | 2.687 | 7.8E-11 | 1.1E-03 | 42.3 | 0.005 | 0.022 | 0.818 |
| Left Hippocampal volume | Periodontitis | rs146607495 | C | T | 0.903 | -60.031 | 4.391 | 1.5E-42 | 5.1E-03 | 186.9 | -0.012 | 0.040 | 0.774 |
| Left Hippocampal volume | Periodontitis | rs17178006 | T | G | 0.883 | 49.303 | 3.990 | 4.5E-35 | 4.1E-03 | 152.7 | -0.046 | 0.045 | 0.304 |
| Left Hippocampal volume | Periodontitis | rs17205972 | G | T | 0.806 | -18.711 | 3.229 | 6.9E-09 | 9.1E-04 | 33.6 | 0.024 | 0.024 | 0.318 |
| Left Hippocampal volume | Periodontitis | rs2287509 | G | T | 0.426 | 17.937 | 2.622 | 7.8E-12 | 1.3E-03 | 46.8 | -0.005 | 0.022 | 0.823 |
| Left Hippocampal volume | Periodontitis | rs2578475 | T | G | 0.657 | 20.297 | 2.723 | 9.0E-14 | 1.5E-03 | 55.6 | 0.031 | 0.023 | 0.173 |
| Left Hippocampal volume | Periodontitis | rs28687399 | C | G | 0.767 | -16.869 | 3.062 | 3.6E-08 | 8.2E-04 | 30.4 | -0.015 | 0.023 | 0.530 |
| Left Hippocampal volume | Periodontitis | rs28758826 | A | G | 0.451 | 14.714 | 2.588 | 1.3E-08 | 8.8E-04 | 32.3 | -0.015 | 0.022 | 0.482 |
| Left Hippocampal volume | Periodontitis | rs35620312 | A | G | 0.772 | -17.678 | 3.100 | 1.2E-08 | 8.8E-04 | 32.5 | -0.013 | 0.023 | 0.561 |
| Left Hippocampal volume | Periodontitis | rs58490349 | T | C | 0.980 | -50.471 | 9.235 | 4.6E-08 | 8.1E-04 | 29.9 | 0.057 | 0.056 | 0.311 |
| Left Hippocampal volume | Periodontitis | rs61785580 | C | T | 0.928 | 30.292 | 5.028 | 1.7E-09 | 9.9E-04 | 36.3 | -0.032 | 0.040 | 0.432 |
| Left Hippocampal volume | Periodontitis | rs6791514 | C | G | 0.276 | 21.116 | 2.880 | 2.3E-13 | 1.5E-03 | 53.7 | -0.004 | 0.024 | 0.875 |
| Left Hippocampal volume | Periodontitis | rs72761270 | C | T | 0.648 | -16.966 | 2.695 | 3.0E-10 | 1.1E-03 | 39.6 | 0.003 | 0.023 | 0.906 |
| Cognitive Performance | Periodontitis | rs1009950 | T | G | 0.418 | -0.019 | 0.003 | 8.2E-11 | 1.8E-04 | 45.5 | 0.013 | 0.022 | 0.569 |
| Cognitive Performance | Periodontitis | rs10129426 | G | A | 0.469 | -0.019 | 0.003 | 1.9E-11 | 1.8E-04 | 47.7 | 0.012 | 0.022 | 0.585 |
| Cognitive Performance | Periodontitis | rs1035738 | T | C | 0.027 | 0.051 | 0.008 | 3.5E-11 | 1.4E-04 | 35.4 | 0.169 | 0.121 | 0.164 |
| Cognitive Performance | Periodontitis | rs10865397 | G | A | 0.522 | 0.017 | 0.003 | 6.8E-09 | 1.4E-04 | 36.1 | 0.019 | 0.022 | 0.396 |
| Cognitive Performance | Periodontitis | rs10875914 | G | A | 0.355 | 0.024 | 0.003 | 3.5E-16 | 2.6E-04 | 66.5 | 0.019 | 0.022 | 0.375 |
| Cognitive Performance | Periodontitis | rs10990610 | T | C | 0.835 | -0.025 | 0.004 | 8.7E-11 | 1.7E-04 | 44.3 | 0.037 | 0.032 | 0.239 |
| Cognitive Performance | Periodontitis | rs11117646 | A | T | 0.191 | -0.022 | 0.004 | 3.7E-10 | 1.6E-04 | 40.2 | 0.011 | 0.030 | 0.714 |
| Cognitive Performance | Periodontitis | rs11138947 | C | T | 0.267 | -0.017 | 0.003 | 4.8E-08 | 1.2E-04 | 30.5 | -0.014 | 0.024 | 0.566 |
| Cognitive Performance | Periodontitis | rs113322852 | A | T | 0.201 | -0.026 | 0.004 | 1.5E-12 | 2.2E-04 | 56.4 | -0.092 | 0.048 | 0.054 |
| Cognitive Performance | Periodontitis | rs1144593 | G | A | 0.299 | 0.023 | 0.003 | 1.8E-13 | 2.2E-04 | 56.0 | -0.013 | 0.023 | 0.556 |
| Cognitive Performance | Periodontitis | rs11662271 | T | C | 0.485 | 0.023 | 0.003 | 4.4E-16 | 2.7E-04 | 69.3 | 0.007 | 0.022 | 0.745 |
| Cognitive Performance | Periodontitis | rs11720523 | C | A | 0.554 | -0.016 | 0.003 | 1.7E-08 | 1.3E-04 | 33.5 | -0.007 | 0.022 | 0.763 |
| Cognitive Performance | Periodontitis | rs12435486 | A | G | 0.248 | -0.018 | 0.003 | 3.8E-08 | 1.2E-04 | 32.0 | 0.032 | 0.026 | 0.230 |
| Cognitive Performance | Periodontitis | rs12439619 | G | T | 0.313 | 0.020 | 0.003 | 7.9E-11 | 1.7E-04 | 44.1 | 0.017 | 0.024 | 0.465 |
| Cognitive Performance | Periodontitis | rs12441495 | G | C | 0.864 | 0.026 | 0.004 | 1.2E-09 | 1.6E-04 | 40.2 | -0.031 | 0.040 | 0.441 |
| Cognitive Performance | Periodontitis | rs12535854 | G | C | 0.667 | 0.018 | 0.003 | 4.5E-09 | 1.4E-04 | 36.4 | 0.018 | 0.024 | 0.447 |
| Cognitive Performance | Periodontitis | rs12536800 | G | C | 0.719 | -0.019 | 0.003 | 1.5E-09 | 1.5E-04 | 39.1 | 0.025 | 0.026 | 0.325 |
| Cognitive Performance | Periodontitis | rs12635303 | C | T | 0.708 | -0.018 | 0.003 | 2.2E-09 | 1.4E-04 | 36.5 | 0.009 | 0.022 | 0.690 |
| Cognitive Performance | Periodontitis | rs12773747 | A | T | 0.226 | 0.021 | 0.003 | 6.7E-10 | 1.5E-04 | 37.9 | 0.023 | 0.024 | 0.329 |
| Cognitive Performance | Periodontitis | rs13120565 | T | A | 0.643 | 0.018 | 0.003 | 5.7E-10 | 1.5E-04 | 39.4 | -0.037 | 0.023 | 0.113 |
| Cognitive Performance | Periodontitis | rs13253386 | T | G | 0.495 | -0.018 | 0.003 | 1.3E-10 | 1.7E-04 | 43.5 | -0.011 | 0.022 | 0.624 |
| Cognitive Performance | Periodontitis | rs13428598 | T | C | 0.379 | 0.020 | 0.003 | 1.3E-11 | 1.8E-04 | 47.5 | 0.035 | 0.023 | 0.120 |
| Cognitive Performance | Periodontitis | rs136554 | A | G | 0.459 | 0.016 | 0.003 | 3.5E-08 | 1.2E-04 | 32.2 | -0.003 | 0.022 | 0.878 |
| Cognitive Performance | Periodontitis | rs1391438 | T | C | 0.315 | 0.017 | 0.003 | 3.3E-08 | 1.2E-04 | 31.8 | 0.017 | 0.023 | 0.458 |
| Cognitive Performance | Periodontitis | rs1408579 | C | T | 0.500 | -0.017 | 0.003 | 4.1E-09 | 1.4E-04 | 36.4 | 0.016 | 0.022 | 0.471 |
| Cognitive Performance | Periodontitis | rs1415802 | T | G | 0.597 | -0.017 | 0.003 | 4.8E-09 | 1.4E-04 | 36.6 | 0.018 | 0.023 | 0.423 |
| Cognitive Performance | Periodontitis | rs1479073 | C | T | 0.311 | 0.017 | 0.003 | 1.9E-08 | 1.3E-04 | 33.1 | -0.013 | 0.024 | 0.589 |
| Cognitive Performance | Periodontitis | rs1507010 | G | A | 0.495 | 0.017 | 0.003 | 1.5E-09 | 1.5E-04 | 38.5 | 0.015 | 0.022 | 0.487 |
| Cognitive Performance | Periodontitis | rs1523048 | T | C | 0.400 | 0.018 | 0.003 | 2.1E-09 | 1.5E-04 | 39.0 | -0.029 | 0.022 | 0.194 |
| Cognitive Performance | Periodontitis | rs1567154 | C | T | 0.735 | 0.020 | 0.003 | 4.5E-09 | 1.6E-04 | 42.0 | -0.027 | 0.029 | 0.347 |
| Cognitive Performance | Periodontitis | rs159428 | T | C | 0.493 | 0.017 | 0.003 | 7.0E-09 | 1.4E-04 | 36.2 | -0.017 | 0.022 | 0.439 |
| Cognitive Performance | Periodontitis | rs17106817 | T | C | 0.716 | 0.019 | 0.003 | 6.9E-09 | 1.4E-04 | 36.0 | 0.046 | 0.025 | 0.063 |
| Cognitive Performance | Periodontitis | rs17428810 | T | C | 0.731 | 0.018 | 0.003 | 8.1E-09 | 1.3E-04 | 32.4 | -0.024 | 0.023 | 0.299 |
| Cognitive Performance | Periodontitis | rs1892419 | C | T | 0.233 | -0.028 | 0.003 | 3.5E-16 | 2.7E-04 | 70.2 | -0.003 | 0.026 | 0.905 |
| Cognitive Performance | Periodontitis | rs2005078 | G | A | 0.333 | 0.019 | 0.003 | 2.4E-10 | 1.6E-04 | 42.4 | 0.015 | 0.025 | 0.542 |
| Cognitive Performance | Periodontitis | rs2143103 | A | G | 0.145 | 0.024 | 0.004 | 9.6E-09 | 1.4E-04 | 36.2 | -0.004 | 0.038 | 0.911 |
| Cognitive Performance | Periodontitis | rs2180111 | A | G | 0.294 | 0.018 | 0.003 | 1.6E-08 | 1.4E-04 | 35.2 | -0.039 | 0.026 | 0.141 |
| Cognitive Performance | Periodontitis | rs2439649 | G | A | 0.461 | -0.016 | 0.003 | 2.0E-08 | 1.3E-04 | 33.0 | -0.001 | 0.022 | 0.971 |
| Cognitive Performance | Periodontitis | rs2478281 | G | A | 0.247 | 0.023 | 0.003 | 3.1E-12 | 1.9E-04 | 49.5 | -0.014 | 0.025 | 0.565 |
| Cognitive Performance | Periodontitis | rs26046 | T | C | 0.367 | -0.020 | 0.003 | 2.4E-12 | 1.9E-04 | 50.1 | -0.013 | 0.022 | 0.562 |
| Cognitive Performance | Periodontitis | rs2647995 | C | T | 0.293 | 0.019 | 0.003 | 5.0E-09 | 1.4E-04 | 37.0 | 0.041 | 0.023 | 0.082 |
| Cognitive Performance | Periodontitis | rs2652454 | T | C | 0.478 | -0.016 | 0.003 | 3.2E-08 | 1.3E-04 | 32.4 | -0.034 | 0.022 | 0.120 |
| Cognitive Performance | Periodontitis | rs2721173 | C | T | 0.551 | 0.017 | 0.003 | 6.1E-09 | 1.4E-04 | 35.2 | -0.007 | 0.022 | 0.752 |
| Cognitive Performance | Periodontitis | rs2737339 | G | A | 0.410 | 0.018 | 0.003 | 4.1E-10 | 1.6E-04 | 41.6 | -0.008 | 0.024 | 0.748 |
| Cognitive Performance | Periodontitis | rs276626 | G | A | 0.162 | -0.021 | 0.004 | 4.8E-08 | 1.2E-04 | 30.1 | -0.063 | 0.029 | 0.030 |
| Cognitive Performance | Periodontitis | rs2799399 | G | T | 0.583 | 0.016 | 0.003 | 4.6E-08 | 1.2E-04 | 31.0 | 0.004 | 0.022 | 0.860 |
| Cognitive Performance | Periodontitis | rs2806048 | A | G | 0.643 | -0.019 | 0.003 | 1.3E-10 | 1.7E-04 | 42.6 | -0.039 | 0.022 | 0.071 |
| Cognitive Performance | Periodontitis | rs2852931 | A | G | 0.849 | 0.028 | 0.004 | 1.1E-10 | 2.1E-04 | 53.5 | 0.030 | 0.034 | 0.387 |
| Cognitive Performance | Periodontitis | rs297589 | A | T | 0.668 | 0.017 | 0.003 | 3.7E-08 | 1.3E-04 | 33.6 | -0.050 | 0.022 | 0.027 |
| Cognitive Performance | Periodontitis | rs2977464 | T | C | 0.168 | 0.021 | 0.004 | 9.9E-09 | 1.2E-04 | 31.6 | 0.011 | 0.026 | 0.683 |
| Cognitive Performance | Periodontitis | rs3128341 | T | C | 0.175 | -0.033 | 0.004 | 2.5E-21 | 3.2E-04 | 83.3 | -0.002 | 0.026 | 0.950 |
| Cognitive Performance | Periodontitis | rs335426 | C | A | 0.500 | -0.020 | 0.003 | 7.2E-12 | 1.9E-04 | 50.2 | 0.036 | 0.022 | 0.099 |
| Cognitive Performance | Periodontitis | rs34802460 | C | T | 0.743 | -0.019 | 0.004 | 4.9E-08 | 1.4E-04 | 36.2 | 0.030 | 0.025 | 0.234 |
| Cognitive Performance | Periodontitis | rs3740422 | G | C | 0.651 | 0.026 | 0.003 | 1.2E-17 | 3.0E-04 | 76.3 | -0.009 | 0.023 | 0.698 |
| Cognitive Performance | Periodontitis | rs3843954 | G | C | 0.750 | 0.021 | 0.003 | 6.3E-10 | 1.6E-04 | 42.0 | 0.028 | 0.024 | 0.245 |
| Cognitive Performance | Periodontitis | rs3860537 | T | C | 0.242 | 0.019 | 0.003 | 3.1E-08 | 1.3E-04 | 32.7 | -0.057 | 0.027 | 0.033 |
| Cognitive Performance | Periodontitis | rs39302 | C | T | 0.808 | 0.021 | 0.004 | 4.5E-09 | 1.4E-04 | 34.9 | -0.020 | 0.029 | 0.487 |
| Cognitive Performance | Periodontitis | rs3943667 | T | C | 0.723 | -0.019 | 0.003 | 3.6E-09 | 1.4E-04 | 35.4 | -0.004 | 0.023 | 0.868 |
| Cognitive Performance | Periodontitis | rs4342312 | C | A | 0.626 | 0.018 | 0.003 | 1.1E-09 | 1.5E-04 | 38.8 | -0.013 | 0.022 | 0.550 |
| Cognitive Performance | Periodontitis | rs4347883 | T | C | 0.561 | -0.016 | 0.003 | 2.8E-08 | 1.3E-04 | 32.3 | -0.013 | 0.022 | 0.550 |
| Cognitive Performance | Periodontitis | rs4463213 | A | G | 0.541 | 0.022 | 0.003 | 2.6E-14 | 2.4E-04 | 61.0 | -0.020 | 0.021 | 0.359 |
| Cognitive Performance | Periodontitis | rs4937860 | G | A | 0.094 | -0.026 | 0.005 | 2.4E-08 | 1.1E-04 | 29.3 | -0.053 | 0.042 | 0.203 |
| Cognitive Performance | Periodontitis | rs4976976 | A | G | 0.386 | 0.020 | 0.003 | 1.2E-11 | 1.9E-04 | 47.8 | -0.003 | 0.022 | 0.882 |
| Cognitive Performance | Periodontitis | rs56135595 | T | G | 0.141 | -0.022 | 0.004 | 3.0E-08 | 1.2E-04 | 30.7 | -0.019 | 0.027 | 0.465 |
| Cognitive Performance | Periodontitis | rs56290130 | A | T | 0.323 | -0.017 | 0.003 | 2.6E-08 | 1.3E-04 | 32.3 | -0.014 | 0.023 | 0.541 |
| Cognitive Performance | Periodontitis | rs5751191 | C | T | 0.478 | -0.022 | 0.003 | 8.9E-15 | 2.4E-04 | 63.1 | -0.056 | 0.022 | 0.010 |
| Cognitive Performance | Periodontitis | rs58489175 | G | A | 0.282 | 0.021 | 0.003 | 2.0E-11 | 1.8E-04 | 47.0 | -0.050 | 0.028 | 0.078 |
| Cognitive Performance | Periodontitis | rs602512 | G | A | 0.614 | -0.019 | 0.003 | 2.9E-11 | 1.8E-04 | 46.4 | 0.010 | 0.025 | 0.686 |
| Cognitive Performance | Periodontitis | rs61815057 | A | G | 0.396 | 0.017 | 0.003 | 8.1E-09 | 1.4E-04 | 34.9 | -0.016 | 0.022 | 0.452 |
| Cognitive Performance | Periodontitis | rs62047970 | T | G | 0.578 | -0.019 | 0.003 | 9.9E-11 | 1.7E-04 | 44.4 | -0.010 | 0.022 | 0.653 |
| Cognitive Performance | Periodontitis | rs620729 | A | C | 0.685 | -0.019 | 0.003 | 3.1E-10 | 1.6E-04 | 40.7 | 0.024 | 0.024 | 0.317 |
| Cognitive Performance | Periodontitis | rs62169190 | T | C | 0.177 | -0.020 | 0.004 | 2.7E-08 | 1.2E-04 | 31.3 | 0.022 | 0.028 | 0.422 |
| Cognitive Performance | Periodontitis | rs6509441 | T | A | 0.769 | -0.019 | 0.003 | 3.3E-08 | 1.3E-04 | 32.3 | 0.057 | 0.028 | 0.040 |
| Cognitive Performance | Periodontitis | rs6535809 | G | A | 0.500 | -0.020 | 0.003 | 6.9E-12 | 1.9E-04 | 49.3 | 0.037 | 0.022 | 0.089 |
| Cognitive Performance | Periodontitis | rs6550835 | G | A | 0.697 | 0.025 | 0.003 | 3.9E-16 | 2.6E-04 | 66.9 | 0.026 | 0.022 | 0.247 |
| Cognitive Performance | Periodontitis | rs6587843 | T | C | 0.471 | 0.018 | 0.003 | 4.8E-10 | 1.6E-04 | 40.4 | 0.025 | 0.022 | 0.245 |
| Cognitive Performance | Periodontitis | rs66752974 | C | A | 0.842 | 0.022 | 0.004 | 7.9E-09 | 1.3E-04 | 33.7 | -0.028 | 0.030 | 0.342 |
| Cognitive Performance | Periodontitis | rs6819372 | G | A | 0.536 | 0.019 | 0.003 | 6.6E-11 | 1.7E-04 | 44.6 | -0.010 | 0.022 | 0.652 |
| Cognitive Performance | Periodontitis | rs6903716 | G | A | 0.274 | -0.018 | 0.003 | 4.6E-09 | 1.3E-04 | 34.8 | -0.021 | 0.023 | 0.352 |
| Cognitive Performance | Periodontitis | rs6952104 | T | C | 0.522 | 0.019 | 0.003 | 6.3E-11 | 1.7E-04 | 44.7 | -0.015 | 0.022 | 0.487 |
| Cognitive Performance | Periodontitis | rs6975134 | C | T | 0.422 | 0.022 | 0.003 | 4.6E-14 | 2.3E-04 | 60.6 | -0.001 | 0.022 | 0.960 |
| Cognitive Performance | Periodontitis | rs702222 | C | T | 0.672 | 0.020 | 0.003 | 1.7E-11 | 1.8E-04 | 45.6 | -0.002 | 0.022 | 0.913 |
| Cognitive Performance | Periodontitis | rs7044246 | T | C | 0.696 | 0.017 | 0.003 | 2.0E-08 | 1.3E-04 | 33.4 | 0.015 | 0.024 | 0.536 |
| Cognitive Performance | Periodontitis | rs7256776 | G | A | 0.709 | 0.019 | 0.003 | 9.3E-10 | 1.4E-04 | 37.0 | -0.005 | 0.022 | 0.806 |
| Cognitive Performance | Periodontitis | rs72739469 | T | C | 0.946 | -0.036 | 0.006 | 7.5E-09 | 1.3E-04 | 34.0 | -0.022 | 0.035 | 0.537 |
| Cognitive Performance | Periodontitis | rs72821233 | T | G | 0.272 | -0.020 | 0.003 | 4.9E-10 | 1.6E-04 | 41.3 | -0.029 | 0.028 | 0.308 |
| Cognitive Performance | Periodontitis | rs7312770 | T | C | 0.514 | -0.016 | 0.003 | 2.5E-08 | 1.3E-04 | 33.0 | 0.010 | 0.022 | 0.632 |
| Cognitive Performance | Periodontitis | rs73845427 | A | G | 0.029 | -0.047 | 0.008 | 7.7E-09 | 1.2E-04 | 31.4 | 0.043 | 0.070 | 0.536 |
| Cognitive Performance | Periodontitis | rs73989053 | A | G | 0.162 | -0.022 | 0.004 | 6.3E-09 | 1.3E-04 | 34.2 | -0.021 | 0.030 | 0.476 |
| Cognitive Performance | Periodontitis | rs74370218 | T | C | 0.383 | -0.018 | 0.003 | 9.8E-10 | 1.5E-04 | 39.5 | 0.000 | 0.023 | 0.987 |
| Cognitive Performance | Periodontitis | rs7573001 | C | G | 0.415 | -0.016 | 0.003 | 2.4E-08 | 1.3E-04 | 34.0 | 0.024 | 0.023 | 0.309 |
| Cognitive Performance | Periodontitis | rs7588384 | C | G | 0.313 | -0.019 | 0.003 | 4.3E-09 | 1.5E-04 | 39.7 | 0.026 | 0.023 | 0.262 |
| Cognitive Performance | Periodontitis | rs75973558 | G | A | 0.119 | -0.025 | 0.004 | 7.4E-09 | 1.3E-04 | 32.8 | 0.043 | 0.039 | 0.275 |
| Cognitive Performance | Periodontitis | rs7599860 | A | C | 0.228 | 0.020 | 0.003 | 3.9E-09 | 1.4E-04 | 35.1 | -0.022 | 0.026 | 0.401 |
| Cognitive Performance | Periodontitis | rs77128898 | T | C | 0.022 | -0.046 | 0.008 | 1.7E-09 | 9.3E-05 | 23.9 | -0.052 | 0.049 | 0.285 |
| Cognitive Performance | Periodontitis | rs78358737 | T | G | 0.966 | -0.042 | 0.007 | 6.2E-10 | 1.2E-04 | 30.6 | 0.038 | 0.055 | 0.491 |
| Cognitive Performance | Periodontitis | rs78382112 | A | G | 0.058 | 0.037 | 0.006 | 8.5E-09 | 1.5E-04 | 38.1 | -0.030 | 0.054 | 0.575 |
| Cognitive Performance | Periodontitis | rs7963801 | C | T | 0.548 | -0.023 | 0.003 | 3.5E-15 | 2.6E-04 | 67.4 | 0.010 | 0.022 | 0.651 |
| Cognitive Performance | Periodontitis | rs8058881 | G | C | 0.250 | 0.018 | 0.003 | 4.1E-08 | 1.3E-04 | 32.4 | 0.004 | 0.024 | 0.851 |
| Cognitive Performance | Periodontitis | rs830383 | A | G | 0.369 | 0.020 | 0.003 | 3.7E-11 | 1.8E-04 | 45.8 | 0.031 | 0.023 | 0.171 |
| Cognitive Performance | Periodontitis | rs875361 | A | G | 0.449 | -0.016 | 0.003 | 1.5E-08 | 1.3E-04 | 33.6 | 0.003 | 0.022 | 0.873 |
| Cognitive Performance | Periodontitis | rs991871 | A | T | 0.755 | 0.024 | 0.003 | 5.7E-13 | 2.2E-04 | 56.3 | -0.005 | 0.026 | 0.860 |
| Cognitive Performance | Periodontitis | rs9930063 | C | T | 0.459 | 0.016 | 0.003 | 1.4E-08 | 1.3E-04 | 34.2 | 0.022 | 0.022 | 0.298 |
| Fluid Intelligence Score | Periodontitis | rs10015590 | A | G | 0.320 | 0.046 | 0.008 | 1.3E-08 | 9.3E-04 | 138.1 | 0.011 | 0.023 | 0.625 |
| Fluid Intelligence Score | Periodontitis | rs10129426 | A | G | 0.541 | 0.046 | 0.008 | 1.5E-09 | 1.1E-03 | 157.8 | -0.012 | 0.022 | 0.585 |
| Fluid Intelligence Score | Periodontitis | rs10402747 | C | T | 0.484 | -0.042 | 0.008 | 3.5E-08 | 8.8E-04 | 131.5 | 0.014 | 0.022 | 0.520 |
| Fluid Intelligence Score | Periodontitis | rs1043254 | A | G | 0.230 | -0.054 | 0.009 | 2.7E-09 | 1.0E-03 | 153.7 | -0.028 | 0.025 | 0.267 |
| Fluid Intelligence Score | Periodontitis | rs1054442 | C | A | 0.374 | 0.047 | 0.008 | 1.7E-09 | 1.0E-03 | 155.3 | 0.023 | 0.022 | 0.281 |
| Fluid Intelligence Score | Periodontitis | rs11678980 | A | G | 0.458 | -0.055 | 0.008 | 2.5E-12 | 1.5E-03 | 223.6 | 0.049 | 0.022 | 0.028 |
| Fluid Intelligence Score | Periodontitis | rs12646225 | T | C | 0.104 | 0.076 | 0.012 | 9.9E-10 | 1.1E-03 | 159.2 | 0.013 | 0.029 | 0.660 |
| Fluid Intelligence Score | Periodontitis | rs13117856 | T | A | 0.288 | -0.046 | 0.008 | 3.5E-08 | 8.7E-04 | 130.1 | 0.036 | 0.024 | 0.127 |
| Fluid Intelligence Score | Periodontitis | rs13262595 | G | A | 0.561 | 0.047 | 0.008 | 7.6E-10 | 1.1E-03 | 162.3 | -0.031 | 0.022 | 0.160 |
| Fluid Intelligence Score | Periodontitis | rs13428598 | T | C | 0.389 | 0.044 | 0.008 | 1.2E-08 | 9.3E-04 | 139.0 | 0.035 | 0.023 | 0.120 |
| Fluid Intelligence Score | Periodontitis | rs1355620 | G | A | 0.322 | 0.052 | 0.008 | 2.4E-10 | 1.2E-03 | 176.6 | 0.029 | 0.022 | 0.201 |
| Fluid Intelligence Score | Periodontitis | rs1389994 | T | C | 0.410 | 0.047 | 0.008 | 1.3E-09 | 1.1E-03 | 158.4 | 0.016 | 0.022 | 0.465 |
| Fluid Intelligence Score | Periodontitis | rs1391438 | C | T | 0.687 | -0.046 | 0.008 | 2.5E-08 | 8.9E-04 | 133.2 | -0.017 | 0.023 | 0.458 |
| Fluid Intelligence Score | Periodontitis | rs1567307 | G | T | 0.202 | -0.053 | 0.009 | 1.6E-08 | 9.1E-04 | 135.7 | -0.036 | 0.029 | 0.216 |
| Fluid Intelligence Score | Periodontitis | rs28667297 | G | A | 0.291 | 0.054 | 0.008 | 8.9E-11 | 1.2E-03 | 180.4 | 0.002 | 0.023 | 0.941 |
| Fluid Intelligence Score | Periodontitis | rs3173680 | A | G | 0.307 | -0.048 | 0.008 | 6.1E-09 | 9.7E-04 | 144.4 | -0.005 | 0.023 | 0.829 |
| Fluid Intelligence Score | Periodontitis | rs31771 | C | T | 0.663 | -0.045 | 0.008 | 3.1E-08 | 9.0E-04 | 133.9 | 0.001 | 0.023 | 0.964 |
| Fluid Intelligence Score | Periodontitis | rs34818820 | A | G | 0.447 | -0.045 | 0.008 | 2.7E-09 | 1.0E-03 | 151.4 | -0.013 | 0.023 | 0.569 |
| Fluid Intelligence Score | Periodontitis | rs424029 | A | G | 0.043 | -0.105 | 0.019 | 1.7E-08 | 9.2E-04 | 136.6 | 0.055 | 0.051 | 0.275 |
| Fluid Intelligence Score | Periodontitis | rs4342312 | A | C | 0.378 | -0.049 | 0.008 | 2.8E-10 | 1.1E-03 | 171.0 | 0.013 | 0.022 | 0.550 |
| Fluid Intelligence Score | Periodontitis | rs4456117 | A | C | 0.509 | -0.043 | 0.008 | 1.2E-08 | 9.3E-04 | 138.9 | -0.026 | 0.021 | 0.223 |
| Fluid Intelligence Score | Periodontitis | rs4463213 | A | G | 0.554 | 0.043 | 0.008 | 1.5E-08 | 9.2E-04 | 136.8 | -0.020 | 0.021 | 0.359 |
| Fluid Intelligence Score | Periodontitis | rs4731365 | A | G | 0.405 | -0.043 | 0.008 | 1.7E-08 | 9.1E-04 | 135.6 | -0.018 | 0.022 | 0.410 |
| Fluid Intelligence Score | Periodontitis | rs4778988 | C | T | 0.313 | 0.047 | 0.008 | 9.9E-09 | 9.5E-04 | 141.4 | 0.034 | 0.024 | 0.146 |
| Fluid Intelligence Score | Periodontitis | rs4852252 | C | T | 0.565 | 0.047 | 0.008 | 7.2E-10 | 1.1E-03 | 161.9 | -0.005 | 0.022 | 0.802 |
| Fluid Intelligence Score | Periodontitis | rs506523 | C | T | 0.348 | 0.044 | 0.008 | 2.9E-08 | 8.8E-04 | 131.6 | 0.009 | 0.024 | 0.712 |
| Fluid Intelligence Score | Periodontitis | rs55837830 | T | C | 0.446 | 0.043 | 0.008 | 2.1E-08 | 9.2E-04 | 136.8 | 0.006 | 0.022 | 0.793 |
| Fluid Intelligence Score | Periodontitis | rs5758605 | A | G | 0.461 | -0.042 | 0.008 | 4.4E-08 | 8.7E-04 | 129.3 | -0.042 | 0.021 | 0.050 |
| Fluid Intelligence Score | Periodontitis | rs6125540 | T | C | 0.425 | -0.047 | 0.008 | 7.0E-10 | 1.1E-03 | 163.2 | -0.012 | 0.021 | 0.574 |
| Fluid Intelligence Score | Periodontitis | rs61768374 | G | T | 0.299 | 0.051 | 0.008 | 9.5E-10 | 1.1E-03 | 159.8 | 0.025 | 0.023 | 0.268 |
| Fluid Intelligence Score | Periodontitis | rs62098051 | G | A | 0.520 | -0.051 | 0.008 | 1.6E-11 | 1.3E-03 | 195.3 | -0.004 | 0.022 | 0.854 |
| Fluid Intelligence Score | Periodontitis | rs62183031 | C | A | 0.297 | -0.046 | 0.008 | 3.7E-08 | 8.7E-04 | 129.4 | -0.018 | 0.024 | 0.444 |
| Fluid Intelligence Score | Periodontitis | rs62268046 | G | A | 0.206 | 0.051 | 0.009 | 5.0E-08 | 8.6E-04 | 127.6 | -0.051 | 0.027 | 0.062 |
| Fluid Intelligence Score | Periodontitis | rs6436555 | C | A | 0.513 | -0.046 | 0.008 | 1.4E-09 | 1.1E-03 | 158.3 | 0.021 | 0.022 | 0.343 |
| Fluid Intelligence Score | Periodontitis | rs6799069 | A | T | 0.313 | -0.045 | 0.008 | 4.5E-08 | 8.6E-04 | 127.9 | -0.018 | 0.023 | 0.420 |
| Fluid Intelligence Score | Periodontitis | rs7010173 | T | C | 0.611 | 0.049 | 0.008 | 2.4E-10 | 1.2E-03 | 172.3 | 0.019 | 0.022 | 0.384 |
| Fluid Intelligence Score | Periodontitis | rs709322 | T | C | 0.074 | -0.081 | 0.014 | 1.8E-08 | 9.1E-04 | 135.8 | 0.026 | 0.041 | 0.518 |
| Fluid Intelligence Score | Periodontitis | rs71454591 | T | C | 0.129 | -0.063 | 0.011 | 3.1E-08 | 8.8E-04 | 131.9 | -0.015 | 0.037 | 0.682 |
| Fluid Intelligence Score | Periodontitis | rs72784651 | T | C | 0.378 | 0.046 | 0.008 | 4.2E-09 | 1.0E-03 | 150.2 | -0.009 | 0.022 | 0.684 |
| Fluid Intelligence Score | Periodontitis | rs72823192 | C | T | 0.259 | -0.054 | 0.009 | 5.7E-10 | 1.1E-03 | 165.3 | -0.023 | 0.029 | 0.417 |
| Fluid Intelligence Score | Periodontitis | rs72839495 | T | A | 0.184 | 0.055 | 0.010 | 2.4E-08 | 9.0E-04 | 133.9 | 0.002 | 0.026 | 0.930 |
| Fluid Intelligence Score | Periodontitis | rs7372302 | C | T | 0.321 | -0.069 | 0.008 | 2.7E-17 | 2.0E-03 | 305.5 | -0.022 | 0.023 | 0.321 |
| Fluid Intelligence Score | Periodontitis | rs77128898 | T | C | 0.035 | -0.114 | 0.021 | 4.8E-08 | 8.9E-04 | 133.1 | -0.052 | 0.049 | 0.285 |
| Fluid Intelligence Score | Periodontitis | rs79254826 | A | G | 0.076 | -0.079 | 0.014 | 4.6E-08 | 8.7E-04 | 130.2 | 0.020 | 0.037 | 0.601 |
| Fluid Intelligence Score | Periodontitis | rs7963801 | C | T | 0.572 | -0.050 | 0.008 | 5.5E-11 | 1.2E-03 | 186.2 | 0.010 | 0.022 | 0.651 |
| Fluid Intelligence Score | Periodontitis | rs8054111 | G | A | 0.728 | 0.050 | 0.009 | 3.8E-09 | 9.9E-04 | 148.4 | 0.048 | 0.026 | 0.065 |
| Fluid Intelligence Score | Periodontitis | rs933827 | A | G | 0.524 | 0.049 | 0.008 | 1.2E-10 | 1.2E-03 | 178.4 | -0.022 | 0.022 | 0.306 |
| Fluid Intelligence Score | Periodontitis | rs9597254 | T | A | 0.321 | 0.046 | 0.008 | 1.9E-08 | 9.3E-04 | 138.6 | -0.028 | 0.024 | 0.254 |
| Fluid Intelligence Score | Periodontitis | rs9597451 | A | G | 0.060 | -0.088 | 0.016 | 3.6E-08 | 8.8E-04 | 131.0 | 0.072 | 0.066 | 0.272 |
| Prospective Memory | Periodontitis | rs10110806 | T | C | 0.630 | -0.009 | 0.002 | 4.1E-06 | 1.4E-04 | 21.2 | -0.016 | 0.022 | 0.465 |
| Prospective Memory | Periodontitis | rs11025859 | G | T | 0.131 | 0.013 | 0.003 | 2.6E-06 | 1.4E-04 | 22.1 | -0.034 | 0.030 | 0.255 |
| Prospective Memory | Periodontitis | rs11126332 | A | G | 0.238 | 0.011 | 0.002 | 7.9E-07 | 1.6E-04 | 24.4 | 0.012 | 0.027 | 0.645 |
| Prospective Memory | Periodontitis | rs113892579 | A | G | 0.034 | 0.026 | 0.005 | 8.0E-07 | 1.6E-04 | 24.4 | 0.053 | 0.058 | 0.361 |
| Prospective Memory | Periodontitis | rs11646834 | C | T | 0.523 | -0.009 | 0.002 | 2.3E-06 | 1.5E-04 | 22.3 | -0.002 | 0.022 | 0.938 |
| Prospective Memory | Periodontitis | rs118061227 | C | T | 0.008 | 0.056 | 0.011 | 2.6E-07 | 1.7E-04 | 26.5 | 0.200 | 0.302 | 0.508 |
| Prospective Memory | Periodontitis | rs12803679 | C | A | 0.327 | 0.010 | 0.002 | 4.6E-07 | 1.7E-04 | 25.4 | -0.055 | 0.023 | 0.015 |
| Prospective Memory | Periodontitis | rs12883954 | G | T | 0.798 | 0.011 | 0.002 | 4.1E-06 | 1.4E-04 | 21.2 | 0.019 | 0.027 | 0.490 |
| Prospective Memory | Periodontitis | rs13173232 | T | C | 0.309 | -0.009 | 0.002 | 3.7E-06 | 1.4E-04 | 21.4 | -0.019 | 0.023 | 0.405 |
| Prospective Memory | Periodontitis | rs138759654 | A | C | 0.013 | 0.039 | 0.008 | 2.9E-06 | 1.4E-04 | 21.9 | -0.103 | 0.160 | 0.520 |
| Prospective Memory | Periodontitis | rs143510783 | T | C | 0.032 | -0.029 | 0.005 | 9.6E-08 | 1.9E-04 | 28.5 | 0.018 | 0.066 | 0.786 |
| Prospective Memory | Periodontitis | rs143885862 | C | T | 0.014 | 0.037 | 0.008 | 4.0E-06 | 1.4E-04 | 21.3 | 0.051 | 0.061 | 0.403 |
| Prospective Memory | Periodontitis | rs144490799 | T | C | 0.020 | 0.034 | 0.007 | 3.4E-06 | 1.4E-04 | 21.6 | 0.119 | 0.133 | 0.371 |
| Prospective Memory | Periodontitis | rs145625753 | T | C | 0.032 | 0.026 | 0.006 | 4.9E-06 | 1.4E-04 | 20.9 | -0.059 | 0.069 | 0.396 |
| Prospective Memory | Periodontitis | rs17001547 | C | T | 0.024 | -0.029 | 0.006 | 3.0E-06 | 1.4E-04 | 21.8 | 0.015 | 0.128 | 0.905 |
| Prospective Memory | Periodontitis | rs17106063 | A | G | 0.162 | 0.012 | 0.003 | 1.9E-06 | 1.5E-04 | 22.7 | -0.004 | 0.036 | 0.902 |
| Prospective Memory | Periodontitis | rs17583177 | T | C | 0.074 | 0.018 | 0.004 | 6.6E-07 | 1.6E-04 | 24.7 | 0.026 | 0.035 | 0.466 |
| Prospective Memory | Periodontitis | rs187507943 | G | A | 0.047 | 0.021 | 0.005 | 2.9E-06 | 1.4E-04 | 21.9 | -0.012 | 0.062 | 0.850 |
| Prospective Memory | Periodontitis | rs27943 | C | T | 0.939 | -0.018 | 0.004 | 3.9E-06 | 1.4E-04 | 21.3 | 0.035 | 0.047 | 0.453 |
| Prospective Memory | Periodontitis | rs2805580 | G | C | 0.606 | 0.010 | 0.002 | 2.2E-07 | 1.8E-04 | 26.8 | 0.013 | 0.023 | 0.557 |
| Prospective Memory | Periodontitis | rs28483211 | C | T | 0.025 | 0.028 | 0.006 | 3.3E-06 | 1.4E-04 | 21.7 | -0.130 | 0.147 | 0.376 |
| Prospective Memory | Periodontitis | rs28537494 | C | T | 0.145 | -0.013 | 0.003 | 3.0E-06 | 1.4E-04 | 21.8 | -0.039 | 0.031 | 0.217 |
| Prospective Memory | Periodontitis | rs2925944 | A | G | 0.021 | 0.034 | 0.007 | 8.6E-07 | 1.6E-04 | 24.2 | 0.004 | 0.063 | 0.955 |
| Prospective Memory | Periodontitis | rs455771 | G | A | 0.780 | -0.011 | 0.002 | 3.9E-06 | 1.4E-04 | 21.3 | 0.017 | 0.028 | 0.552 |
| Prospective Memory | Periodontitis | rs4934572 | C | A | 0.018 | 0.033 | 0.007 | 2.2E-06 | 1.5E-04 | 22.4 | 0.017 | 0.067 | 0.806 |
| Prospective Memory | Periodontitis | rs55701287 | T | C | 0.098 | 0.015 | 0.003 | 1.8E-06 | 1.5E-04 | 22.8 | -0.024 | 0.034 | 0.478 |
| Prospective Memory | Periodontitis | rs56223232 | A | G | 0.045 | 0.021 | 0.005 | 4.9E-06 | 1.4E-04 | 20.9 | -0.083 | 0.071 | 0.239 |
| Prospective Memory | Periodontitis | rs60350712 | G | A | 0.015 | 0.038 | 0.008 | 9.6E-07 | 1.6E-04 | 24.0 | 0.118 | 0.171 | 0.492 |
| Prospective Memory | Periodontitis | rs61957386 | A | G | 0.049 | 0.022 | 0.004 | 9.5E-07 | 1.6E-04 | 24.0 | -0.055 | 0.056 | 0.321 |
| Prospective Memory | Periodontitis | rs62009918 | T | C | 0.234 | -0.015 | 0.002 | 3.2E-11 | 2.9E-04 | 44.1 | 0.056 | 0.029 | 0.055 |
| Prospective Memory | Periodontitis | rs62397635 | A | G | 0.099 | 0.016 | 0.003 | 7.2E-07 | 1.6E-04 | 24.6 | 0.002 | 0.041 | 0.956 |
| Prospective Memory | Periodontitis | rs6702781 | C | T | 0.012 | -0.044 | 0.009 | 4.2E-07 | 1.7E-04 | 25.6 | -0.221 | 0.226 | 0.329 |
| Prospective Memory | Periodontitis | rs73040360 | T | C | 0.147 | 0.014 | 0.003 | 4.7E-07 | 1.7E-04 | 25.4 | 0.002 | 0.027 | 0.925 |
| Prospective Memory | Periodontitis | rs73093577 | T | C | 0.123 | -0.014 | 0.003 | 6.0E-07 | 1.6E-04 | 24.9 | -0.051 | 0.026 | 0.051 |
| Prospective Memory | Periodontitis | rs73424022 | A | G | 0.282 | -0.010 | 0.002 | 1.2E-06 | 1.5E-04 | 23.6 | 0.041 | 0.023 | 0.072 |
| Prospective Memory | Periodontitis | rs7342602 | C | T | 0.816 | 0.011 | 0.002 | 4.9E-06 | 1.4E-04 | 20.9 | 0.021 | 0.028 | 0.452 |
| Prospective Memory | Periodontitis | rs77309031 | G | T | 0.042 | 0.023 | 0.005 | 1.5E-06 | 1.5E-04 | 23.2 | 0.112 | 0.037 | 0.002 |
| Prospective Memory | Periodontitis | rs7814166 | T | A | 0.629 | -0.010 | 0.002 | 8.6E-07 | 1.6E-04 | 24.2 | -0.016 | 0.022 | 0.463 |
| Prospective Memory | Periodontitis | rs8039474 | T | C | 0.938 | -0.020 | 0.004 | 4.6E-07 | 1.7E-04 | 25.4 | 0.030 | 0.043 | 0.477 |
| Prospective Memory | Periodontitis | rs9306943 | T | A | 0.153 | 0.014 | 0.003 | 8.2E-08 | 1.9E-04 | 28.8 | 0.013 | 0.030 | 0.672 |
| Prospective Memory | Periodontitis | rs9549218 | A | G | 0.093 | -0.015 | 0.003 | 4.8E-06 | 1.4E-04 | 20.9 | 0.017 | 0.045 | 0.704 |
| Prospective Memory | Periodontitis | rs9858813 | A | G | 0.094 | -0.016 | 0.003 | 5.9E-07 | 1.6E-04 | 25.0 | -0.054 | 0.046 | 0.238 |
| Reaction Time | Periodontitis | rs1054442 | C | A |  | 0.012 | 0.002 | 2.4E-12 | 1.5E-04 | 49.1 | 0.023 | 0.022 | 0.281 |
| Reaction Time | Periodontitis | rs10911301 | G | A |  | -0.010 | 0.002 | 1.8E-09 | 1.1E-04 | 36.1 | -0.032 | 0.022 | 0.141 |
| Reaction Time | Periodontitis | rs13028903 | T | C |  | 0.013 | 0.002 | 1.7E-14 | 1.8E-04 | 58.8 | -0.021 | 0.022 | 0.329 |
| Reaction Time | Periodontitis | rs13219424 | T | C |  | -0.010 | 0.002 | 1.3E-08 | 9.8E-05 | 32.3 | 0.005 | 0.022 | 0.812 |
| Reaction Time | Periodontitis | rs1351848 | T | C |  | 0.011 | 0.002 | 9.5E-10 | 1.1E-04 | 37.4 | 0.007 | 0.022 | 0.754 |
| Reaction Time | Periodontitis | rs1385253 | C | T |  | -0.010 | 0.002 | 1.7E-08 | 9.6E-05 | 31.8 | -0.013 | 0.024 | 0.572 |
| Reaction Time | Periodontitis | rs16822665 | T | C |  | -0.010 | 0.002 | 4.9E-09 | 1.0E-04 | 34.2 | -0.010 | 0.022 | 0.649 |
| Reaction Time | Periodontitis | rs16959783 | T | C |  | 0.010 | 0.002 | 1.8E-08 | 9.6E-05 | 31.7 | -0.031 | 0.024 | 0.191 |
| Reaction Time | Periodontitis | rs1734197 | T | C |  | 0.010 | 0.002 | 2.2E-08 | 9.5E-05 | 31.3 | 0.023 | 0.023 | 0.316 |
| Reaction Time | Periodontitis | rs2040879 | C | T |  | 0.011 | 0.002 | 3.3E-10 | 1.2E-04 | 39.5 | -0.016 | 0.036 | 0.658 |
| Reaction Time | Periodontitis | rs2604268 | T | C |  | 0.012 | 0.002 | 9.2E-12 | 1.4E-04 | 46.5 | 0.013 | 0.025 | 0.605 |
| Reaction Time | Periodontitis | rs323299 | T | G |  | -0.010 | 0.002 | 1.3E-08 | 9.8E-05 | 32.3 | 0.041 | 0.023 | 0.069 |
| Reaction Time | Periodontitis | rs35066740 | T | G |  | 0.010 | 0.002 | 3.7E-09 | 1.1E-04 | 34.8 | 0.019 | 0.027 | 0.470 |
| Reaction Time | Periodontitis | rs4627212 | G | A |  | 0.010 | 0.002 | 1.9E-09 | 1.1E-04 | 36.1 | -0.011 | 0.023 | 0.625 |
| Reaction Time | Periodontitis | rs4673905 | G | A |  | 0.012 | 0.002 | 4.0E-12 | 1.5E-04 | 48.1 | 0.011 | 0.023 | 0.631 |
| Reaction Time | Periodontitis | rs61786416 | G | A |  | 0.010 | 0.002 | 1.7E-08 | 9.7E-05 | 31.9 | 0.019 | 0.031 | 0.530 |
| Reaction Time | Periodontitis | rs6870103 | T | G |  | 0.010 | 0.002 | 1.9E-09 | 1.1E-04 | 36.1 | -0.024 | 0.022 | 0.264 |
| Reaction Time | Periodontitis | rs7075591 | A | G |  | 0.010 | 0.002 | 1.3E-08 | 9.8E-05 | 32.3 | 0.000 | 0.022 | 0.995 |
| Reaction Time | Periodontitis | rs75543711 | A | G |  | -0.009 | 0.002 | 4.6E-08 | 9.1E-05 | 29.9 | 0.091 | 0.036 | 0.012 |
| Reaction Time | Periodontitis | rs77998199 | G | A |  | 0.011 | 0.002 | 2.0E-10 | 1.2E-04 | 40.5 | 0.018 | 0.030 | 0.538 |
| Reaction Time | Periodontitis | rs925229 | G | A |  | 0.010 | 0.002 | 4.7E-08 | 9.0E-05 | 29.9 | -0.025 | 0.022 | 0.239 |
| Reaction Time | Periodontitis | rs936414 | G | A |  | 0.010 | 0.002 | 4.9E-08 | 9.0E-05 | 29.7 | 0.016 | 0.024 | 0.518 |
| Alzheimer's disease | Periodontitis | rs1065712 | C | G | 0.053 | 0.108 | 0.019 | 5.5E-09 | 1.2E-03 | 572.5 | -0.027 | 0.037 | 0.462 |
| Alzheimer's disease | Periodontitis | rs10792832 | A | G | 0.358 | -0.106 | 0.008 | 6.3E-36 | 5.1E-03 | 2511.2 | -0.001 | 0.023 | 0.976 |
| Alzheimer's disease | Periodontitis | rs10933431 | C | G | 0.766 | 0.085 | 0.010 | 1.0E-17 | 2.6E-03 | 1273.1 | 0.010 | 0.025 | 0.704 |
| Alzheimer's disease | Periodontitis | rs11218343 | T | C | 0.961 | 0.165 | 0.021 | 1.0E-14 | 2.1E-03 | 1001.8 | 0.016 | 0.067 | 0.809 |
| Alzheimer's disease | Periodontitis | rs112481437 | A | G | 0.028 | 0.294 | 0.028 | 4.9E-26 | 4.7E-03 | 2304.7 | -0.008 | 0.077 | 0.923 |
| Alzheimer's disease | Periodontitis | rs113706587 | A | G | 0.110 | 0.093 | 0.013 | 3.4E-12 | 1.7E-03 | 820.1 | -0.014 | 0.035 | 0.690 |
| Alzheimer's disease | Periodontitis | rs1140239 | T | C | 0.379 | -0.059 | 0.009 | 4.6E-12 | 1.6E-03 | 805.5 | 0.018 | 0.022 | 0.414 |
| Alzheimer's disease | Periodontitis | rs117618017 | T | C | 0.144 | 0.113 | 0.012 | 1.7E-21 | 3.1E-03 | 1538.6 | -0.007 | 0.033 | 0.844 |
| Alzheimer's disease | Periodontitis | rs11769559 | T | C | 0.594 | 0.070 | 0.009 | 4.0E-16 | 2.3E-03 | 1138.3 | -0.009 | 0.022 | 0.687 |
| Alzheimer's disease | Periodontitis | rs12151021 | A | G | 0.336 | 0.106 | 0.009 | 4.1E-30 | 5.0E-03 | 2432.2 | 0.012 | 0.024 | 0.632 |
| Alzheimer's disease | Periodontitis | rs12444183 | A | G | 0.387 | -0.059 | 0.008 | 2.2E-12 | 1.7E-03 | 809.4 | 0.042 | 0.023 | 0.065 |
| Alzheimer's disease | Periodontitis | rs12590654 | A | G | 0.328 | -0.069 | 0.009 | 2.1E-15 | 2.1E-03 | 1034.1 | 0.045 | 0.023 | 0.055 |
| Alzheimer's disease | Periodontitis | rs143332484 | T | C | 0.013 | 0.335 | 0.038 | 6.0E-19 | 2.8E-03 | 1361.9 | 0.064 | 0.120 | 0.596 |
| Alzheimer's disease | Periodontitis | rs148601586 | C | G | 0.986 | -0.648 | 0.037 | 2.7E-70 | 1.2E-02 | 5927.0 | -0.057 | 0.200 | 0.776 |
| Alzheimer's disease | Periodontitis | rs1582763 | A | G | 0.371 | -0.086 | 0.008 | 1.7E-24 | 3.5E-03 | 1688.6 | -0.028 | 0.024 | 0.249 |
| Alzheimer's disease | Periodontitis | rs1693551 | T | C | 0.533 | -0.046 | 0.008 | 1.8E-08 | 1.0E-03 | 511.9 | 0.014 | 0.022 | 0.514 |
| Alzheimer's disease | Periodontitis | rs1800978 | C | G | 0.870 | -0.068 | 0.012 | 1.6E-08 | 1.1E-03 | 513.5 | 0.014 | 0.035 | 0.697 |
| Alzheimer's disease | Periodontitis | rs190651665 | T | G | 0.970 | 0.160 | 0.026 | 8.4E-10 | 1.5E-03 | 723.3 | -0.060 | 0.126 | 0.633 |
| Alzheimer's disease | Periodontitis | rs2070902 | T | C | 0.254 | -0.054 | 0.009 | 1.0E-08 | 1.1E-03 | 533.2 | 0.030 | 0.024 | 0.218 |
| Alzheimer's disease | Periodontitis | rs2154481 | T | C | 0.524 | 0.050 | 0.008 | 1.0E-09 | 1.2E-03 | 608.8 | -0.012 | 0.022 | 0.569 |
| Alzheimer's disease | Periodontitis | rs2526377 | A | G | 0.555 | 0.045 | 0.008 | 4.1E-08 | 1.0E-03 | 492.4 | 0.004 | 0.022 | 0.871 |
| Alzheimer's disease | Periodontitis | rs2830489 | T | C | 0.281 | -0.055 | 0.009 | 1.7E-09 | 1.2E-03 | 590.0 | 0.001 | 0.024 | 0.968 |
| Alzheimer's disease | Periodontitis | rs28577986 | A | G | 0.263 | 0.070 | 0.011 | 3.4E-10 | 1.9E-03 | 923.0 | -0.008 | 0.023 | 0.740 |
| Alzheimer's disease | Periodontitis | rs365653 | A | G | 0.892 | 0.256 | 0.015 | 1.8E-68 | 1.3E-02 | 6244.8 | -0.016 | 0.038 | 0.681 |
| Alzheimer's disease | Periodontitis | rs4292 | T | C | 0.617 | 0.069 | 0.008 | 3.5E-16 | 2.2E-03 | 1093.1 | 0.008 | 0.022 | 0.723 |
| Alzheimer's disease | Periodontitis | rs4485362 | T | G | 0.540 | 0.046 | 0.008 | 2.0E-08 | 1.1E-03 | 519.8 | -0.003 | 0.022 | 0.904 |
| Alzheimer's disease | Periodontitis | rs4714447 | T | C | 0.345 | 0.058 | 0.009 | 1.7E-11 | 1.5E-03 | 737.1 | -0.002 | 0.023 | 0.918 |
| Alzheimer's disease | Periodontitis | rs563366239 | A | G | 0.988 | 0.298 | 0.043 | 3.3E-12 | 2.1E-03 | 1044.3 | -0.015 | 0.090 | 0.863 |
| Alzheimer's disease | Periodontitis | rs57402520 | A | G | 0.122 | 0.091 | 0.013 | 6.6E-13 | 1.8E-03 | 855.1 | 0.008 | 0.029 | 0.776 |
| Alzheimer's disease | Periodontitis | rs5848 | T | C | 0.289 | 0.065 | 0.009 | 1.8E-12 | 1.7E-03 | 836.8 | -0.027 | 0.023 | 0.241 |
| Alzheimer's disease | Periodontitis | rs593742 | A | G | 0.705 | 0.061 | 0.009 | 1.0E-11 | 1.5E-03 | 755.4 | -0.034 | 0.022 | 0.118 |
| Alzheimer's disease | Periodontitis | rs6014724 | A | G | 0.910 | 0.118 | 0.015 | 4.8E-16 | 2.3E-03 | 1104.6 | -0.010 | 0.048 | 0.840 |
| Alzheimer's disease | Periodontitis | rs61762319 | A | G | 0.974 | -0.143 | 0.026 | 2.1E-08 | 1.0E-03 | 511.1 | -0.004 | 0.076 | 0.960 |
| Alzheimer's disease | Periodontitis | rs62375397 | T | C | 0.210 | 0.075 | 0.010 | 8.6E-14 | 1.8E-03 | 903.1 | -0.039 | 0.027 | 0.155 |
| Alzheimer's disease | Periodontitis | rs6586028 | T | C | 0.804 | 0.079 | 0.010 | 1.3E-14 | 2.0E-03 | 964.7 | -0.044 | 0.026 | 0.087 |
| Alzheimer's disease | Periodontitis | rs6656401 | A | G | 0.188 | 0.125 | 0.010 | 2.8E-33 | 4.8E-03 | 2349.1 | 0.039 | 0.027 | 0.158 |
| Alzheimer's disease | Periodontitis | rs67250450 | T | C | 0.788 | 0.056 | 0.010 | 2.0E-08 | 1.0E-03 | 510.4 | 0.010 | 0.028 | 0.707 |
| Alzheimer's disease | Periodontitis | rs6733839 | T | C | 0.389 | 0.169 | 0.008 | 6.5E-90 | 1.4E-02 | 6678.3 | 0.003 | 0.023 | 0.894 |
| Alzheimer's disease | Periodontitis | rs6846529 | T | C | 0.717 | -0.067 | 0.009 | 1.3E-13 | 1.8E-03 | 897.0 | -0.020 | 0.027 | 0.472 |
| Alzheimer's disease | Periodontitis | rs7068231 | T | G | 0.403 | -0.049 | 0.008 | 6.8E-09 | 1.1E-03 | 556.8 | 0.000 | 0.022 | 0.993 |
| Alzheimer's disease | Periodontitis | rs73223431 | T | C | 0.369 | 0.066 | 0.008 | 5.3E-15 | 2.0E-03 | 979.4 | -0.010 | 0.022 | 0.645 |
| Alzheimer's disease | Periodontitis | rs7384878 | T | C | 0.690 | 0.078 | 0.009 | 2.1E-18 | 2.6E-03 | 1255.9 | -0.034 | 0.023 | 0.145 |
| Alzheimer's disease | Periodontitis | rs74504435 | A | G | 0.907 | 0.084 | 0.014 | 2.0E-09 | 1.2E-03 | 586.6 | 0.070 | 0.034 | 0.040 |
| Alzheimer's disease | Periodontitis | rs74685827 | T | G | 0.981 | -0.196 | 0.030 | 8.6E-11 | 1.4E-03 | 687.5 | -0.089 | 0.101 | 0.375 |
| Alzheimer's disease | Periodontitis | rs7912495 | A | G | 0.538 | -0.057 | 0.008 | 2.9E-12 | 1.6E-03 | 794.2 | 0.024 | 0.022 | 0.268 |
| Alzheimer's disease | Periodontitis | rs867230 | A | C | 0.603 | 0.101 | 0.008 | 1.5E-33 | 4.9E-03 | 2387.7 | 0.001 | 0.022 | 0.950 |
| Alzheimer's disease | Periodontitis | rs9676738 | A | G | 0.042 | 0.169 | 0.023 | 1.2E-13 | 2.3E-03 | 1119.2 | -0.064 | 0.044 | 0.140 |
| Alzheimer's disease | Periodontitis | rs976271 | A | G | 0.361 | 0.047 | 0.008 | 3.2E-08 | 1.0E-03 | 491.2 | 0.006 | 0.022 | 0.773 |
| Lewy body dementia | Periodontitis | rs2230288 | T | C | 0.009 | 1.061 | 0.149 | 1.3E-12 | 2.0E-02 | 136.5 | 0.021 | 0.055 | 0.705 |
| Lewy body dementia | Periodontitis | rs6599388 | T | C | 0.310 | 0.220 | 0.040 | 3.5E-08 | 2.1E-02 | 140.5 | 0.034 | 0.024 | 0.166 |
| Lewy body dementia | Periodontitis | rs6733839 | T | C | 0.362 | 0.227 | 0.039 | 4.2E-09 | 2.4E-02 | 160.5 | 0.003 | 0.023 | 0.894 |
| Lewy body dementia | Periodontitis | rs7680557 | C | A | 0.504 | -0.240 | 0.037 | 9.7E-11 | 2.9E-02 | 196.6 | -0.021 | 0.022 | 0.338 |
| Vascular Dementia | Periodontitis | rs10408790 | A | G | 0.430 | -0.311 | 0.068 | 4.3E-06 | 4.7E-02 | 20403.6 | -0.021 | 0.022 | 0.338 |
| Vascular Dementia | Periodontitis | rs17881670 | C | T | 0.024 | 2.248 | 0.484 | 3.3E-06 | 2.3E-01 | 124865.0 | -0.037 | 0.147 | 0.804 |
| Vascular Dementia | Periodontitis | rs36038448 | T | C | 0.056 | 1.021 | 0.222 | 4.0E-06 | 1.1E-01 | 51241.7 | -0.092 | 0.180 | 0.611 |
| Vascular Dementia | Periodontitis | rs529543 | G | T | 0.395 | -0.312 | 0.068 | 4.7E-06 | 4.6E-02 | 20010.9 | -0.018 | 0.022 | 0.410 |
| Vascular Dementia | Periodontitis | rs538579727 | T | G | 0.037 | 1.543 | 0.323 | 1.8E-06 | 1.7E-01 | 85196.0 | 0.027 | 0.121 | 0.822 |
| Vascular Dementia | Periodontitis | rs79891963 | T | C | 0.056 | 1.128 | 0.232 | 1.2E-06 | 1.3E-01 | 63344.6 | -0.092 | 0.073 | 0.210 |
|  |  |  |  |  |  |  |  |  |  |  |  |  |  |
| **Change rate in brain structure** | |  |  |  |  |  |  |  |  |  |  |  |  |
| Cortical thickness | Periodontitis | rs17006251 | A | C | 0.202 | -0.965 | 0.205 | 2.4E-06 | 1.4E-03 | 22.2 | 0.019 | 0.032 | 0.551 |
| Cortical thickness | Periodontitis | rs564158 | A | G | 0.826 | 1.025 | 0.216 | 2.1E-06 | 1.4E-03 | 22.5 | 0.016 | 0.028 | 0.563 |
| Cortical thickness | Periodontitis | rs595269 | A | C | 0.931 | 1.794 | 0.382 | 2.6E-06 | 1.4E-03 | 22.1 | -0.040 | 0.071 | 0.574 |
| Cortical thickness | Periodontitis | rs6435231 | T | C | 0.329 | -0.957 | 0.190 | 4.9E-07 | 1.6E-03 | 25.3 | 0.039 | 0.025 | 0.119 |
| Cortical thickness | Periodontitis | rs72698183 | T | G | 0.261 | -0.942 | 0.195 | 1.4E-06 | 1.5E-03 | 23.3 | 0.029 | 0.024 | 0.220 |
| Brain surface area | Periodontitis | rs11955994 | A | G | 0.159 | -58.727 | 12.03 | 1.1E-06 | 1.5E-03 | 23.8 | 0.039 | 0.032 | 0.212 |
| Brain surface area | Periodontitis | rs4740274 | A | G | 0.527 | 31.060 | 6.750 | 4.2E-06 | 1.4E-03 | 21.2 | -0.011 | 0.022 | 0.610 |
| Brain surface area | Periodontitis | rs58313139 | T | C | 0.071 | 62.257 | 12.83 | 1.2E-06 | 1.5E-03 | 23.5 | 0.036 | 0.050 | 0.476 |
| Brain surface area | Periodontitis | rs61895068 | A | G | 0.879 | -48.160 | 9.909 | 1.2E-06 | 1.5E-03 | 23.6 | -0.072 | 0.040 | 0.076 |
| Brain surface area | Periodontitis | rs66620635 | T | C | 0.131 | -54.004 | 11.19 | 1.4E-06 | 1.5E-03 | 23.3 | -0.036 | 0.029 | 0.224 |
| Hippocampal volume | Periodontitis | rs1913699 | A | C | 0.467 | -3.908 | 0.827 | 2.3E-06 | 1.4E-03 | 22.3 | 0.039 | 0.022 | 0.069 |
| Hippocampal volume | Periodontitis | rs231400 | A | T | 0.093 | 7.427 | 1.564 | 2.1E-06 | 1.4E-03 | 22.5 | -0.019 | 0.051 | 0.709 |
| Hippocampal volume | Periodontitis | rs362636 | T | C | 0.295 | 4.736 | 1.002 | 2.3E-06 | 1.4E-03 | 22.4 | -0.013 | 0.022 | 0.572 |
| Hippocampal volume | Periodontitis | rs4321178 | T | C | 0.560 | -4.915 | 1.076 | 5.0E-06 | 1.3E-03 | 20.9 | -0.012 | 0.022 | 0.589 |
| Total brain volume | Periodontitis | rs10790497 | A | G | 0.654 | 262.52 | 56.89 | 4.0E-06 | 1.4E-03 | 21.3 | -0.010 | 0.025 | 0.685 |
| Total brain volume | Periodontitis | rs12325429 | A | G | 0.433 | -303.84 | 55.34 | 4.0E-08 | 1.9E-03 | 30.1 | 0.011 | 0.022 | 0.605 |
| Total brain volume | Periodontitis | rs1880248 | T | C | 0.800 | 349.83 | 71.37 | 9.5E-07 | 1.5E-03 | 24.0 | 0.007 | 0.025 | 0.788 |
| Total brain volume | Periodontitis | rs35648592 | A | C | 0.578 | 275.28 | 59.13 | 3.2E-06 | 1.4E-03 | 21.7 | -0.026 | 0.022 | 0.237 |
| Total brain volume | Periodontitis | rs4141409 | T | G | 0.369 | -259.51 | 55.87 | 3.4E-06 | 1.4E-03 | 21.6 | 0.007 | 0.023 | 0.767 |
| Total brain volume | Periodontitis | rs55833149 | T | G | 0.133 | -405.39 | 84.94 | 1.8E-06 | 1.5E-03 | 22.8 | -0.013 | 0.028 | 0.655 |
| Total brain volume | Periodontitis | rs61804371 | T | C | 0.882 | 405.08 | 87.32 | 3.5E-06 | 1.4E-03 | 21.5 | -0.026 | 0.033 | 0.443 |
| Total brain volume | Periodontitis | rs71612691 | A | C | 0.137 | 380.75 | 80.93 | 2.5E-06 | 1.4E-03 | 22.1 | -0.029 | 0.028 | 0.307 |
| Total brain volume | Periodontitis | rs7601178 | A | G | 0.622 | -275.60 | 59.80 | 4.1E-06 | 1.4E-03 | 21.2 | 0.002 | 0.022 | 0.921 |
|  |  |  |  |  |  |  |  |  |  |  |  |  |  |
| **Slope of cognitive decline** | |  |  |  |  |  |  |  |  |  |  |  |  |
| Executive function | Periodontitis | rs10004897 | A | G |  | 0.250 | 0.054 | 4.4E-06 | 1.8E-02 | 20.5 | -0.068 | 0.036 | 0.059 |
| Executive function | Periodontitis | rs2546753 | A | G |  | -0.210 | 0.045 | 2.9E-06 | 1.7E-02 | 20.2 | 0.002 | 0.024 | 0.947 |
| Executive function | Periodontitis | rs34393106 | A | C |  | 0.280 | 0.061 | 4.4E-06 | 3.8E-02 | 45.4 | 0.007 | 0.023 | 0.753 |
| Executive function | Periodontitis | rs6545794 | A | G |  | -0.330 | 0.071 | 2.9E-06 | 1.6E-02 | 18.6 | 0.048 | 0.033 | 0.144 |
| Visuospatial skill | Periodontitis | rs2075199 | T | C |  | -0.260 | 0.057 | 4.3E-06 | 2.2E-02 | 25.3 | 0.034 | 0.027 | 0.211 |
| Visuospatial skill | Periodontitis | rs57169846 | A | G |  | 0.220 | 0.047 | 2.8E-06 | 1.9E-02 | 22.2 | 0.004 | 0.025 | 0.881 |
| Attention/processing speed | Periodontitis | rs1403649 | C | T |  | -0.210 | 0.043 | 8.7E-07 | 2.0E-02 | 23.1 | 0.010 | 0.023 | 0.668 |
| Attention/processing speed | Periodontitis | rs17532412 | A | C |  | 0.280 | 0.051 | 3.4E-08 | 2.2E-02 | 25.9 | -0.014 | 0.028 | 0.620 |
| Attention/processing speed | Periodontitis | rs658424 | C | T |  | -0.260 | 0.057 | 4.4E-06 | 1.6E-02 | 18.9 | 0.008 | 0.026 | 0.757 |
| Attention/processing speed | Periodontitis | rs66671632 | T | C |  | -0.280 | 0.059 | 2.5E-06 | 1.7E-02 | 19.2 | 0.047 | 0.036 | 0.197 |
| Attention/processing speed | Periodontitis | rs7332702 | C | T |  | -0.240 | 0.051 | 2.7E-06 | 1.8E-02 | 20.6 | 0.010 | 0.027 | 0.705 |
| Memory | Periodontitis | rs2295752 | A | C |  | -0.230 | 0.050 | 4.3E-06 | 1.9E-02 | 21.8 | 0.022 | 0.024 | 0.345 |
| Memory | Periodontitis | rs2897044 | A | C |  | 0.240 | 0.050 | 1.6E-06 | 1.9E-02 | 22.3 | -0.022 | 0.026 | 0.397 |
| Memory | Periodontitis | rs3991624 | G | A |  | 0.290 | 0.059 | 7.4E-07 | 2.0E-02 | 23.6 | 0.031 | 0.029 | 0.300 |

A1: Effect Allele; A2: Other Allele; AD: Alzheimer's disease
