# Supplementary material for: No genetic causal associations between periodontitis and brain atrophy or cognitive impairment: evidence from a comprehensive bidirectional Mendelian randomization study
Source: BMC Oral Health. 2024 May 16;24:571. doi: 10.1186/s12903-024-04367-7 (PMC11100120; doi:10.1186/s12903-024-04367-7)
Supplement: Supplementary file 3 — Supplementary Material 3: Table S3. Information about all confounders-related SNPs. [file 12903_2024_4367_MOESM3_ESM.docx]

**Supplementary Table 3. Information about all confounders-related SNPs**

| **Exposure** | **SNP** | **Locus(hg19)** | **Locus(hg38)** | **Confounders** |
| --- | --- | --- | --- | --- |
| Cortical Surface Area | rs2802292 | chr6:108908518 | chr6:108587315 | BMI |
|  | rs11759026 | chr6:126792095 | chr6:126470949 | Diabetes |
|  | rs12357321 | chr10:21790476 | chr10:21501547 | BMI |
|  | rs10876864 | chr12:56401085 | chr12:56007301 | BMI, Inflammation, Diabetes, Alcohol consumption |
|  |  |  |  |  |
| Right Hippocampal volume | rs3800232 | chr6:108998953 | chr6:108677750 | BMI |
|  | rs4787495 | chr16:30165725 | chr16:30154404 | BMI |
|  | rs8614 | chr17:27588806 | chr17:29261788 | Alcohol consumption, Smoking |
|  |  |  |  |  |
|  |  |  |  |  |
| Left Hippocampal volume | rs61192764 | chr6:108995187 | chr6:108673984 | BMI |
|  | rs11245347 | chr10:126427037 | chr10:124738468 | BMI |
|  | rs12373078 | chr16:30187676 | chr16:30176355 | BMI |
|  | rs2732711 | chr17:44350293 | chr17:46272927 | BMI, Inflammation, Alcohol consumption |
|  | rs61192764 | chr6:108995187 | chr6:108673984 | BMI |
|  | rs11245347 | chr10:126427037 | chr10:124738468 | BMI |
|  | rs12373078 | chr16:30187676 | chr16:30176355 | BMI |
|  |  |  |  |  |
| Cognitive Performance | rs2352974 | chr3:49890613 | chr3:49853180 | BMI, Diabetes |
|  | rs1906252 | chr6:98550289 | chr6:98102413 | BMI, Smoking, Alcohol consumption |
|  | rs13107325 | chr4:103188709 | chr4:102267552 | BMI |
|  | rs9384679 | chr6:108864419 | chr6:108543216 | BMI |
|  | rs11793831 | chr9:23362311 | chr9:23362313 | BMI |
|  | rs148696809 | chr6:28934352 | chr6:28966575 | Obesity, Inflammation, Diabetes |
|  | rs12448902 | chr16:28871191 | chr16:28859870 | BMI, Alcohol consumption |
|  | rs8054299 | chr16:53498655 | chr16:53464743 | BMI |
|  | rs11123820 | chr2:100871186 | chr2:100254724 | BMI, Alcohol consumption |
|  | rs13163336 | chr5:87943710 | chr5:88647892 | BMI |
|  | rs34811474 | chr4:25408838 | chr4:25407216 | BMI |
|  | rs10189857 | chr2:60713235 | chr2:60486100 | BMI, Smoking |
|  | rs2239647 | chr14:33292743 | chr14:32823537 | BMI |
|  | rs3735478 | chr7:44800176 | chr7:44760577 | BMI, Smoking |
|  | rs11693702 | chr2:162802184 | chr2:161945674 | Smoking |
|  | rs9436866 | chr1:69427576 | chr1:68961893 | BMI |
|  | rs2295499 | chr4:2717690 | chr4:2715963 | BMI |
|  | rs11079849 | chr17:47090785 | chr17:49013423 | BMI, Alcohol consumption, Diabetes |
|  | rs5757670 | chr22:39829736 | chr22:39433731 | Inflammation |
|  | rs1064608 | chr11:47640429 | chr11:47618877 | BMI, Smoking, Alcohol consumption |
|  | rs7626560 | chr3:12475088 | chr3:12433589 | BMI |
|  | rs11210871 | chr1:44029353 | chr1:43563682 | Smoking, Alcohol consumption |
|  | rs4744250 | chr9:96271752 | chr9:93509470 | BMI |
|  | rs17049085 | chr2:57948003 | chr2:57720868 | BMI |
|  | rs73189617 | chr3:184069126 | chr3:184351338 | BMI, Smoking |
|  | rs889169 | chr19:47548678 | chr19:47045420 | BMI |
|  | rs6798941 | chr3:52893465 | chr3:52859449 | BMI |
|  | rs6860626 | chr5:59636727 | chr5:60340900 | Diabetes |
|  | rs35853157 | chr4:140780768 | chr4:139859614 | BMI |
|  | rs6708515 | chr2:41606931 | chr2:41379791 | BMI |
|  | rs1812587 | chr5:62991802 | chr5:63695975 | BMI |
|  | rs11259916 | chr15:84361518 | chr15:83692766 | BMI |
|  | rs2836921 | chr21:40516070 | chr21:39144144 | BMI |
|  |  |  |  |  |
| Fluid Intelligence Score | rs9436866 | chr1:69427576 | chr1:68961893 | BMI |
|  | rs12128707 | chr1:72588119 | chr1:72122436 | BMI |
|  | rs6678734 | chr1:96176563 | chr1:95711007 | Alcohol consumption |
|  | rs7599488 | chr2:60718347 | chr2:60491212 | BMI, Diabetes |
|  | rs12712072 | chr2:100797912 | chr2:100181450 | BMI |
|  | rs2352974 | chr3:49890613 | chr3:49853180 | BMI, Diabetes, Alcohol consumption |
|  | rs1529675 | chr3:71640776 | chr3:71591625 | BMI, Alcohol consumption |
|  | rs9853960 | chr3:136487989 | chr3:136769147 | BMI |
|  | rs34811474 | chr4:25408838 | chr4:25407216 | BMI |
|  | rs13107325 | chr4:103188709 | chr4:102267552 | BMI |
|  | rs141729694 | chr5:87999371 | chr5:88703554 | BMI |
|  | rs148257233 | chr5:102623315 | chr5:103287614 | Diabetes |
|  | rs7775835 | chr6:28678357 | chr6:28710580 | BMI, Diabetes, Smoking |
|  | rs1487441 | chr6:98553894 | chr6:98106018 | BMI, Alcohol consumption, Smoking |
|  | rs9384679 | chr6:108864419 | chr6:108543216 | BMI |
|  | rs3735478 | chr7:44800176 | chr7:44760577 | BMI |
|  | rs11793831 | chr9:23362311 | chr9:23362313 | BMI |
|  | rs75742406 | chr11:17070365 | chr11:17048818 | Smoking |
|  | rs11605348 | chr11:47606483 | chr11:47584931 | BMI, Alcohol consumption, Smoking |
|  | rs7146202 | chr14:33303517 | chr14:32834311 | BMI, Smoking |
|  | rs214271 | chr14:73632670 | chr14:73165962 | Alcohol consumption |
|  | rs865719 | chr16:28379443 | chr16:28368122 | BMI, Alcohol consumption |
|  | rs7189726 | chr16:53497774 | chr16:53463862 | BMI |
|  | rs80048023 | chr19:18227357 | chr19:18116547 | BMI |
|  | rs5750830 | chr22:39840828 | chr22:39444823 | Inflammation |
|  |  |  |  |  |
| Prospective Memory | rs1451533 | chr2:105466005 | chr2:104849547 | BMI |
|  | rs13107325 | chr4:103188709 | chr4:102267552 | BMI |
|  | rs1233578 | chr6:28712247 | chr6:28744470 | Inflammation, Diabetes |
|  | rs9388612 | chr6:128312324 | chr6:127991179 | Smoking, Alcohol consumption |
|  | rs7790230 | chr7:50522857 | chr7:50455159 | Alcohol consumption |
|  | rs7777391 | chr7:117621877 | chr7:117981823 | Alcohol consumption |
|  | rs9410446 | chr9:91326422 | chr9:88711507 | Obesity |
|  | rs11191050 | chr10:103331152 | chr10:101571395 | Obesity |
|  | rs1821446 | chr18:44894422 | chr18:47368051 | BMI |
|  | rs2838000 | chr21:42646518 | chr21:41274591 | BMI |
|  |  |  |  |  |
| Reaction Time | rs648997 | chr12:111976776 | chr12:111538972 | Obesity, Smoking |
|  | rs10775404 | chr17:44167366 | chr17:46090000 | Obesity |
|  | rs66534382 | chr19:4056366 | chr19:4056368 | BMI |
|  | rs11205668 | chr1:50416830 | chr1:49951158 | BMI |
|  | rs264979 | chr2:104269262 | chr2:103652804 | BMI, Alcohol consumption |
|  | rs56335290 | chr5:112036634 | chr5:112700937 | BMI |
|  | rs4946935 | chr6:109000742 | chr6:108679539 | BMI |
|  | rs10125715 | chr9:86528858 | chr9:83913943 | Alcohol consumption |
|  |  |  |  |  |
| Alzheimer's disease | rs4690197 | chr4:953974 | chr4:960186 | Obesity |
|  | rs4734295 | chr8:96000919 | chr8:94988691 | Diabetes |
|  | rs34173062 | chr8:145158607 | chr8:144103704 | BMI |
|  | rs11500477 | chr11:47409051 | chr11:47387500 | BMI |
|  | rs17125924 | chr14:53391680 | chr14:52924962 | BMI |
|  | rs7225002 | chr17:44189067 | chr17:46111701 | Inflammation |
|  | rs12972720 | chr19:1854151 | chr19:1854152 | BMI |
|  | rs117310449 | chr19:45393516 | chr19:44890259 | BMI |
|  | rs61679753 | chr19:45400747 | chr19:44897490 | BMI |
|  |  |  |  |  |
| Lewy body dementia | rs769449 | chr19:45410002 | chr19:44906745 | BMI, Diabetes |
|  |  |  |  |  |
| Vascular Dementia | rs429358 | chr19:45411941 | chr19:44908684 | BMI |
