# Supplementary material for: No genetic causal associations between periodontitis and brain atrophy or cognitive impairment: evidence from a comprehensive bidirectional Mendelian randomization study
Source: BMC Oral Health. 2024 May 16;24:571. doi: 10.1186/s12903-024-04367-7 (PMC11100120; doi:10.1186/s12903-024-04367-7)
Supplement: Supplementary file 4 — Supplementary Material 4: Table S4. Cochran's Q test for heterogeneity and MR-Egger test for directional pleiotropy. [file 12903_2024_4367_MOESM4_ESM.docx]

**Supplementary Table 4.** Cochran's Q test for heterogeneity and MR-Egger test for directional pleiotropy.

| **Exposure** | **Outcome** | **Heterogeneity** | | | | **Pleiotropy*** | | | |
| --- | --- | --- | --- | --- | --- | --- | --- | --- | --- |
|  |  | **Cochran's Q** | | **df** | **P-value** | **Egger_intercept** | | **SE** | **P-value** |
| **Exploration Cohort (GLIDE)** | | | | | | | | | |
| Periodontitis | Cortical Surface Area | 2.478 | | 3 | 0.479 | 117.476 | | 159.054 | 0.537 |
| Periodontitis | Cortical Thickness | 2.922 | | 3 | 0.404 | 0.000 | | 0.001 | 0.822 |
| Periodontitis | Right Hippocampal volume | 0.798 | | 4 | 0.939 | 0.166 | | 2.542 | 0.952 |
| Periodontitis | Left Hippocampal volume | 2.312 | | 4 | 0.679 | 0.150 | | 2.398 | 0.954 |
| Periodontitis | Cognitive Performance | 4.677 | | 5 | 0.457 | 0.004 | | 0.002 | 0.123 |
| Periodontitis | Fluid Intelligence Score | 1.231 | | 5 | 0.942 | 0.005 | | 0.006 | 0.478 |
| Periodontitis | Prospective Memory | 1.421 | | 5 | 0.922 | 0.000 | | 0.002 | 0.843 |
| Periodontitis | Reaction Time | 2.982 | | 5 | 0.703 | -0.001 | | 0.001 | 0.471 |
| Periodontitis | Alzheimer's disease (AD) | 6.209 | | 5 | 0.286 | 0.014 | | 0.007 | 0.103 |
| Periodontitis | Early-onset AD | 2.289 | | 5 | 0.808 | 0.036 | | 0.030 | 0.300 |
| Periodontitis | Late-onset AD | 4.174 | | 5 | 0.525 | 0.006 | | 0.015 | 0.726 |
| Periodontitis | Lewy body dementia | 1.744 | | 5 | 0.883 | 0.031 | | 0.030 | 0.357 |
| Periodontitis | Vascular Dementia | 6.883 | | 5 | 0.230 | 0.069 | | 0.063 | 0.334 |
| Periodontitis | Frontotemporal Dementia | 7.661 | | 5 | 0.176 | -0.070 | | 0.130 | 0.617 |
|  |  |  |  | |  |  |  | |  |
| Cortical Surface Area | Periodontitis | 3.778 | 5 | | 0.582 | 0.014 | 0.025 | | 0.604 |
| Cortical thickness | Periodontitis | 5.722 | 6 | | 0.455 | -0.006 | 0.037 | | 0.874 |
| Right Hippocampal volume | Periodontitis | 28.117 | 12 | | 0.005 | 0.029 | 0.016 | | 0.103 |
| Left Hippocampal volume | Periodontitis | 24.758 | 11 | | 0.010 | 0.034 | 0.018 | | 0.085 |
| Cognitive Performance | Periodontitis | 82.321 | 88 | | 0.651 | 0.005 | 0.010 | | 0.647 |
| Fluid Intelligence Score | Periodontitis | 32.158 | 45 | | 0.925 | 0.005 | 0.015 | | 0.740 |
| Prospective Memory | Periodontitis | 32.873 | 37 | | 0.663 | 0.002 | 0.011 | | 0.835 |
| Reaction Time | Periodontitis | 17.851 | 21 | | 0.658 | -0.018 | 0.038 | | 0.651 |
| Alzheimer's disease (AD) | Periodontitis | 56.615 | 41 | | 0.053 | 0.001 | 0.008 | | 0.924 |
| Early-onset AD | Periodontitis | 13.676 | 16 | | 0.623 | 0.004 | 0.010 | | 0.694 |
| Late-onset AD | Periodontitis | 25.414 | 33 | | 0.825 | 0.002 | 0.008 | | 0.801 |
| Lewy body dementia | Periodontitis | 0.194 | 2 | | 0.908 | 0.005 | 0.026 | | 0.880 |
| Vascular Dementia | Periodontitis | 4.888 | 4 | | 0.299 | 0.002 | 0.028 | | 0.939 |
| Frontotemporal Dementia | Periodontitis | 2.132 | 1 | | 0.144 |  |  | |  |
|  |  |  |  | |  |  |  | |  |
| **Change rate in brain structure** | |  |  | |  |  |  | |  |
| Cortical thickness | Periodontitis | 3.826 | 5 | | 0.575 | -0.054 | 0.036 | | 0.207 |
| Brain surface area | Periodontitis | 5.108 | 4 | | 0.276 | -0.019 | 0.054 | | 0.749 |
| Hippocampal volume | Periodontitis | 0.353 | 1 | | 0.552 |  |  | |  |
| Total brain volume | Periodontitis | 7.993 | 8 | | 0.434 | 0.034 | 0.040 | | 0.414 |
|  |  |  |  | |  |  |  | |  |
| **Slope of cognitive decline** | |  |  | |  |  |  | |  |
| Executive function | Periodontitis | 0.444 | 3 | | 0.931 | 0.041 | 0.067 | | 0.603 |
| Visuospatial skill | Periodontitis | 1.697 | 1 | | 0.193 |  |  | |  |
| Attention/processing speed | Periodontitis | 4.082 | 4 | | 0.395 | -0.028 | 0.091 | | 0.777 |
| Memory | Periodontitis | 13.480 | 2 | | 0.001 | 1.775 | 0.484 | | 0.169 |
|  |  |  |  | |  |  |  | |  |
|  |  |  |  | |  |  |  | |  |
| **Replication Cohort (FinnGen)** | | | | | | | | | |
| Periodontitis | Cortical Surface Area | 13.710 | 15 | | 0.548 | 7.484 | 112.880 | | 0.948 |
| Periodontitis | Cortical Thickness | 19.279 | 17 | | 0.313 | -0.001 | 0.001 | | 0.082 |
| Periodontitis | Right Hippocampal volume | 17.502 | 14 | | 0.230 | 0.783 | 3.676 | | 0.835 |
| Periodontitis | Left Hippocampal volume | 11.631 | 14 | | 0.636 | 1.462 | 2.993 | | 0.633 |
| Periodontitis | Cognitive Performance | 22.534 | 13 | | 0.048 | 0.005 | 0.004 | | 0.224 |
| Periodontitis | Fluid Intelligence Score | 20.343 | 14 | | 0.120 | 0.003 | 0.008 | | 0.746 |
| Periodontitis | Prospective Memory | 16.344 | 16 | | 0.429 | 0.000 | 0.002 | | 0.827 |
| Periodontitis | Reaction Time | 28.732 | 13 | | 0.007 | -0.001 | 0.002 | | 0.773 |
| Periodontitis | Alzheimer's disease | 22.714 | 17 | | 0.159 | 0.003 | 0.008 | | 0.727 |
| Periodontitis | Lewy body dementia | 6.445 | 14 | | 0.954 | -0.025 | 0.043 | | 0.567 |
| Periodontitis | Vascular Dementia | 15.500 | 16 | | 0.488 | -0.004 | 0.055 | | 0.937 |
|  |  |  |  | |  |  |  | |  |
| Cortical Surface Area | Periodontitis | 4.099 | 6 | | 0.663 | -0.028 | 0.034 | | 0.447 |
| Cortical Thickness | Periodontitis | 8.367 | 6 | | 0.212 | 0.001 | 0.047 | | 0.991 |
| Right Hippocampal volume | Periodontitis | 13.528 | 16 | | 0.634 | 0.003 | 0.016 | | 0.846 |
| Left Hippocampal volume | Periodontitis | 8.185 | 13 | | 0.831 | 0.013 | 0.017 | | 0.431 |
| Cognitive Performance | Periodontitis | 105.293 | 102 | | 0.392 | -0.013 | 0.013 | | 0.326 |
| Fluid Intelligence Score | Periodontitis | 48.121 | 48 | | 0.468 | 0.014 | 0.019 | | 0.466 |
| Prospective Memory | Periodontitis | 44.356 | 41 | | 0.332 | -0.011 | 0.015 | | 0.457 |
| Reaction Time | Periodontitis | 22.561 | 21 | | 0.368 | -0.039 | 0.055 | | 0.486 |
| Alzheimer's disease | Periodontitis | 34.368 | 47 | | 0.915 | -0.015 | 0.009 | | 0.089 |
| Lewy body dementia | Periodontitis | 1.510 | 3 | | 0.680 | 0.018 | 0.023 | | 0.509 |
| Vascular Dementia | Periodontitis | 3.532 | 5 | | 0.619 | 0.035 | 0.024 | | 0.226 |
|  |  |  |  | |  |  |  | |  |
|  |  |  |  | |  |  |  | |  |
| **Change rate in brain structure** | |  |  | |  |  |  | |  |
| Cortical thickness | Periodontitis | 2.503 | 4 | | 0.644 | -0.022 | 0.086 | | 0.813 |
| Brain surface area | Periodontitis | 6.555 | 4 | | 0.161 | -0.022 | 0.077 | | 0.797 |
| Hippocampal volume | Periodontitis | 3.087 | 3 | | 0.378 | -0.068 | 0.076 | | 0.468 |
| Total brain volume | Periodontitis | 2.529 | 8 | | 0.960 | -0.018 | 0.049 | | 0.726 |
|  |  |  |  | |  |  |  | |  |
| **Slope of cognitive decline** | |  |  | |  |  |  | |  |
| Executive function | Periodontitis | 4.196 | 3 | | 0.241 | 0.039 | 0.121 | | 0.779 |
| Visuospatial skill | Periodontitis | 0.920 | 1 | | 0.337 |  |  | |  |
| Attention/processing speed | Periodontitis | 0.819 | 4 | | 0.936 | 0.035 | 0.109 | | 0.766 |
| Memory | Periodontitis | 2.520 | 2 | | 0.284 | -0.238 | 0.152 | | 0.362 |

***** In part of the analysis, pleiotropy test was not available since the trait had an effective SNP number of less than two as an exposure.
