# Supplementary material for: No genetic causal associations between periodontitis and brain atrophy or cognitive impairment: evidence from a comprehensive bidirectional Mendelian randomization study
Source: BMC Oral Health. 2024 May 16;24:571. doi: 10.1186/s12903-024-04367-7 (PMC11100120; doi:10.1186/s12903-024-04367-7)
Supplement: Supplementary file 5 — Supplementary Material 5: Table S5. MR-PRESSO test for heterogeneity and outlier exclusion. [file 12903_2024_4367_MOESM5_ESM.docx]

**Supplementary Table 5.** MR-PRESSO test for heterogeneity and outlier exclusion.

| **Exposure** | **Outcome** | **MR Analysis** | **Causal Estimate** | **Sd** | **T-stat** | **P-value** | **RSSobs** | **Global_test_P** |
| --- | --- | --- | --- | --- | --- | --- | --- | --- |
| **Exploration Cohort (GLIDE)** | | | | | | | | |
| Periodontitis | Cortical Surface Area | MR-PRESSO | -785.968 | 618.796 | -1.270 | 0.294 | 4.472 | 0.519 |
| Periodontitis | Cortical thickness | MR-PRESSO | -0.005 | 0.005 | -1.049 | 0.371 | 5.637 | 0.450 |
| Periodontitis | Right Hippocampal volume | MR-PRESSO | -3.834 | 4.612 | -0.831 | 0.452 | 1.075 | 0.955 |
| Periodontitis | Left Hippocampal volume | MR-PRESSO | -5.224 | 7.401 | -0.706 | 0.519 | 3.110 | 0.755 |
| Periodontitis | Cognitive Performance | MR-PRESSO | 0.001 | 0.007 | 0.215 | 0.838 | 5.800 | 0.582 |
| Periodontitis | Fluid Intelligence Score | MR-PRESSO | -0.013 | 0.010 | -1.262 | 0.263 | 1.878 | 0.937 |
| Periodontitis | Prospective Memory | MR-PRESSO | -0.001 | 0.003 | -0.502 | 0.637 | 1.755 | 0.947 |
| Periodontitis | Reaction Time | MR-PRESSO | 0.001 | 0.001 | 1.896 | 0.116 | 3.198 | 0.845 |
| Periodontitis | Alzheimer's disease (AD) | MR-PRESSO | 0.009 | 0.023 | 0.378 | 0.721 | 7.223 | 0.460 |
| Periodontitis | Early-onset AD | MR-PRESSO | -0.032 | 0.062 | -0.522 | 0.624 | 5.514 | 0.700 |
| Periodontitis | Late-onset AD | MR-PRESSO | -0.043 | 0.042 | -1.025 | 0.352 | 4.869 | 0.682 |
| Periodontitis | Lewy body dementia | MR-PRESSO | -0.072 | 0.054 | -1.331 | 0.241 | 2.938 | 0.859 |
| Periodontitis | Vascular Dementia | MR-PRESSO | 0.133 | 0.208 | 0.642 | 0.549 | 9.737 | 0.365 |
| Periodontitis | Frontotemporal Dementia | MR-PRESSO | -0.373 | 0.368 | -1.014 | 0.357 | 14.237 | 0.348 |
| Cortical Surface Area | Periodontitis | MR-PRESSO | 2.8E-06 | 6.8E-06 | 0.414 | 0.696 | 5.941 | 0.572 |
| Cortical thickness | Periodontitis | MR-PRESSO | -0.108 | 1.334 | -0.081 | 0.938 | 8.221 | 0.444 |
| Right Hippocampal volume | Periodontitis | MR-PRESSO | -2.3E-04 | 3.2E-04 | -0.706 | 0.494 | 32.613 | 0.007 |
| Left Hippocampal volume | Periodontitis | MR-PRESSO | -1.0E-04 | 3.7E-04 | -0.274 | 0.789 | 30.421 | 0.012 |
| Cognitive Performance | Periodontitis | MR-PRESSO | -0.079 | 0.091 | -0.865 | 0.389 | 84.076 | 0.657 |
| Fluid Intelligence Score | Periodontitis | MR-PRESSO | -0.056 | 0.044 | -1.272 | 0.210 | 33.507 | 0.930 |
| Prospective Memory | Periodontitis | MR-PRESSO | -0.549 | 0.274 | -2.006 | 0.052 | 34.676 | 0.677 |
| Reaction Time | Periodontitis | MR-PRESSO | -0.367 | 0.321 | -1.141 | 0.267 | 19.808 | 0.646 |
| Alzheimer's disease (AD) | Periodontitis | MR-PRESSO | -0.087 | 0.042 | -2.073 | 0.044 | 58.644 | 0.064 |
| Early-onset AD | Periodontitis | MR-PRESSO | 0.002 | 0.015 | 0.156 | 0.878 | 15.734 | 0.646 |
| Late-onset AD | Periodontitis | MR-PRESSO | 0.014 | 0.021 | 0.649 | 0.521 | 26.807 | 0.831 |
| Lewy body dementia | Periodontitis | MR-PRESSO | -0.001 | 0.027 | -0.033 | 0.975 | 7.581 | 0.338 |
| Vascular Dementia | Periodontitis | MR-PRESSO | -0.001 | 0.027 | -0.033 | 0.975 | 7.581 | 0.353 |
|  |  |  |  |  |  |  |  |  |
| **Change rate in brain structure** | |  |  |  |  |  |  |  |
| Cortical thickness | Periodontitis | MR-PRESSO | -0.006 | 0.007 | -0.818 | 0.451 | 5.458 | 0.600 |
| Brain surface area | Periodontitis | MR-PRESSO | 0.000 | 0.000 | 0.234 | 0.827 | 9.052 | 0.305 |
| Total brain volume | Periodontitis | MR-PRESSO | 0.000 | 0.000 | -1.063 | 0.319 | 11.011 | 0.401 |
|  |  |  |  |  |  |  |  |  |
| **Slope of cognitive decline** | |  |  |  |  |  |  |  |
| Executive function | Periodontitis | MR-PRESSO | -0.054 | 0.017 | -3.243 | 0.048 | 0.794 | 0.936 |
| Attention/processing speed | Periodontitis | MR-PRESSO | 0.014 | 0.036 | 0.376 | 0.726 | 6.455 | 0.426 |
|  |  |  |  |  |  |  |  |  |
|  |  |  |  |  |  |  |  |  |
|  |  |  |  |  |  |  |  |  |
| **Replication Cohort (FinnGen)** | | | | | | | | |
| Periodontitis | Cortical Surface Area | MR-PRESSO | 246.432 | 267.296 | 0.922 | 0.371 | 16.255 | 0.513 |
| Periodontitis | Cortical Thickness | MR-PRESSO | -0.002 | 0.002 | -1.119 | 0.279 | 22.483 | 0.291 |
| Periodontitis | Right Hippocampal volume | MR-PRESSO | -6.191 | 7.737 | -0.800 | 0.437 | 19.562 | 0.258 |
| Periodontitis | Left Hippocampal volume | MR-PRESSO | -6.360 | 5.948 | -1.069 | 0.303 | 13.135 | 0.660 |
| Periodontitis | Cognitive Performance | MR-PRESSO | -0.006 | 0.010 | -0.551 | 0.591 | 24.934 | 0.069 |
| Periodontitis | Fluid Intelligence Score | MR-PRESSO | -0.019 | 0.020 | -0.908 | 0.379 | 22.155 | 0.172 |
| Periodontitis | Prospective Memory | MR-PRESSO | 0.004 | 0.004 | 0.870 | 0.397 | 19.324 | 0.408 |
| Periodontitis | Reaction Time | MR-PRESSO | 0.006 | 0.003 | 1.716 | 0.110 | 33.000 | 0.011 |
| Periodontitis | Alzheimer's disease | MR-PRESSO | -0.020 | 0.020 | -1.004 | 0.329 | 25.168 | 0.181 |
| Periodontitis | Lewy body dementia | MR-PRESSO | 0.008 | 0.063 | 0.119 | 0.907 | 7.068 | 0.962 |
| Periodontitis | Vascular Dementia | MR-PRESSO | -0.103 | 0.142 | -0.724 | 0.480 | 16.900 | 0.530 |
|  |  |  |  |  |  |  |  |  |
| Cortical Surface Area | Periodontitis | MR-PRESSO | 1.7E-05 | 8.1E-06 | 2.045 | 0.087 | 5.946 | 0.652 |
| Cortical thickness | Periodontitis | MR-PRESSO | -3.582 | 2.022 | -1.772 | 0.127 | 10.284 | 0.312 |
| Right Hippocampal volume | Periodontitis | MR-PRESSO | -5.0E-05 | 2.4E-04 | -0.213 | 0.834 | 15.003 | 0.649 |
| Left Hippocampal volume | Periodontitis | MR-PRESSO | -5.4E-05 | 2.4E-04 | -0.226 | 0.825 | 9.482 | 0.835 |
| Cognitive Performance | Periodontitis | MR-PRESSO | -0.026 | 0.122 | -0.216 | 0.829 | 107.264 | 0.399 |
| Fluid Intelligence Score | Periodontitis | MR-PRESSO | 0.077 | 0.068 | 1.139 | 0.261 | 50.124 | 0.478 |
| Prospective Memory | Periodontitis | MR-PRESSO | 0.280 | 0.388 | 0.721 | 0.475 | 47.290 | 0.311 |
| Reaction Time | Periodontitis | MR-PRESSO | -0.068 | 0.497 | -0.137 | 0.892 | 24.494 | 0.385 |
| Alzheimer's disease | Periodontitis | MR-PRESSO | -0.063 | 0.038 | -1.636 | 0.109 | 35.799 | 0.917 |
| Lewy body dementia | Periodontitis | MR-PRESSO | 0.047 | 0.027 | 1.719 | 0.184 | 3.018 | 0.693 |
| Vascular Dementia | Periodontitis | MR-PRESSO | 0.002 | 0.026 | 0.091 | 0.931 | 5.466 | 0.603 |
|  |  |  |  |  |  |  |  |  |
| **Change rate in brain structure** | |  |  |  |  |  |  |  |
| Cortical thickness | Periodontitis | MR-PRESSO | -0.020 | 0.010 | -1.974 | 0.120 | 4.239 | 0.641 |
| Brain surface area | Periodontitis | MR-PRESSO | 0.000 | 0.000 | 0.502 | 0.642 | 10.901 | 0.215 |
| Hippocampal volume | Periodontitis | MR-PRESSO | -0.003 | 0.003 | -0.977 | 0.401 | 5.858 | 0.422 |
| Total brain volume | Periodontitis | MR-PRESSO | 0.000 | 0.000 | -2.029 | 0.077 | 3.294 | 0.958 |
|  |  |  |  |  |  |  |  |  |
| **Slope of cognitive decline** | |  |  |  |  |  |  |  |
| Executive function | Periodontitis | MR-PRESSO | -0.065 | 0.061 | -1.068 | 0.364 | 7.785 | 0.337 |
| Attention/processing speed | Periodontitis | MR-PRESSO | -0.060 | 0.022 | -2.718 | 0.053 | 1.143 | 0.952 |

* In part of the analysis, MR-PRESSO test was not available since the trait had not enough SNP as an exposure.
