# Supplementary material for: No genetic causal associations between periodontitis and brain atrophy or cognitive impairment: evidence from a comprehensive bidirectional Mendelian randomization study
Source: BMC Oral Health. 2024 May 16;24:571. doi: 10.1186/s12903-024-04367-7 (PMC11100120; doi:10.1186/s12903-024-04367-7)
Supplement: Supplementary file 7 — Supplementary Material 7: Figure S2. Scatter plots of causal relationships of brain atrophy measures and cognitive impairment with periodontitis utilizing different MR methods (In exploration cohort). [file 12903_2024_4367_MOESM7_ESM.docx]

Supplementary Figure 2. Scatter plots of causal relationships of brain atrophy measures and cognitive impairment with periodontitis utilizing different MR methods (In exploration cohort). A through L show causal estimates of different characteristics for periodontitis, respectively. A: Cortical surface area. B: Cortex thickness. C: Right Hippocampal volume. D: Left Hippocampal volume. E: Cognitive performance. F: Fluid intelligence score. G: Prospective memory. H: Reaction time. I: Alzheimer’s disease. J: Lewy body dementia. K: Vascular dementia. L: Frontotemporal dementia. The slope of each line corresponds to the causality estimate for each method. The effect of a single SNP on the outcome (dots and vertical lines) is delineated in the background with its effect on exposure (dots and horizontal lines).
