# Supplementary material for: No genetic causal associations between periodontitis and brain atrophy or cognitive impairment: evidence from a comprehensive bidirectional Mendelian randomization study
Source: BMC Oral Health. 2024 May 16;24:571. doi: 10.1186/s12903-024-04367-7 (PMC11100120; doi:10.1186/s12903-024-04367-7)
Supplement: Supplementary file 8 — Supplementary Material 8: Figure S3. Estimated causal effects of periodontitis with early-onset AD and late-onset AD using different MR methods in exploration cohort (forward and reverse direction). [file 12903_2024_4367_MOESM8_ESM.docx]

Supplementary Figure 3. Estimated causal effects of periodontitis with early-onset AD and late-onset AD using different MR methods in exploration cohort (forward and reverse direction). IVW: inverse variance weighted; MR-RAPS: MR- robust adjusted profile score; N.snps: number of SNPs used in MR. AD: Alzheimer's disease.
