# Supplementary material for: No genetic causal associations between periodontitis and brain atrophy or cognitive impairment: evidence from a comprehensive bidirectional Mendelian randomization study
Source: BMC Oral Health. 2024 May 16;24:571. doi: 10.1186/s12903-024-04367-7 (PMC11100120; doi:10.1186/s12903-024-04367-7)
Supplement: Supplementary file 9 — Supplementary Material 9: Figure S4. Scatter plots and leave-one-out analysis of periodontitis with early-onset AD and late-onset AD in exploration cohort (forward and reverse direction). [file 12903_2024_4367_MOESM9_ESM.docx]

Supplementary Figure 4. Scatter plots and leave-one-out analysis of periodontitis with early-onset AD and late-onset AD in exploration cohort (forward and reverse direction). A to D show the causal estimates through scatter plots. A: periodontitis on early-onset AD. B: early-onset AD on periodontitis. C: periodontitis on late-onset AD. D: late-onset AD on periodontitis. The slope of each line corresponds to the causality estimate for each method. The effect of a single SNP on the outcome (dots and vertical lines) is delineated in the background with its effect on exposure (dots and horizontal lines). E to H show the leave-one-out analysis. E: periodontitis on early-onset AD. F: early-onset AD on periodontitis. G: periodontitis on late-onset AD. H: late-onset AD on periodontitis.
