# Supplementary material for: No genetic causal associations between periodontitis and brain atrophy or cognitive impairment: evidence from a comprehensive bidirectional Mendelian randomization study
Source: BMC Oral Health. 2024 May 16;24:571. doi: 10.1186/s12903-024-04367-7 (PMC11100120; doi:10.1186/s12903-024-04367-7)
Supplement: Supplementary file 12 — Supplementary Material 12: Figure S7.docx Leave-one-out analysis of periodontitis with brain atrophy measures and cognitive impairment (In exploration cohort). [file 12903_2024_4367_MOESM12_ESM.docx]

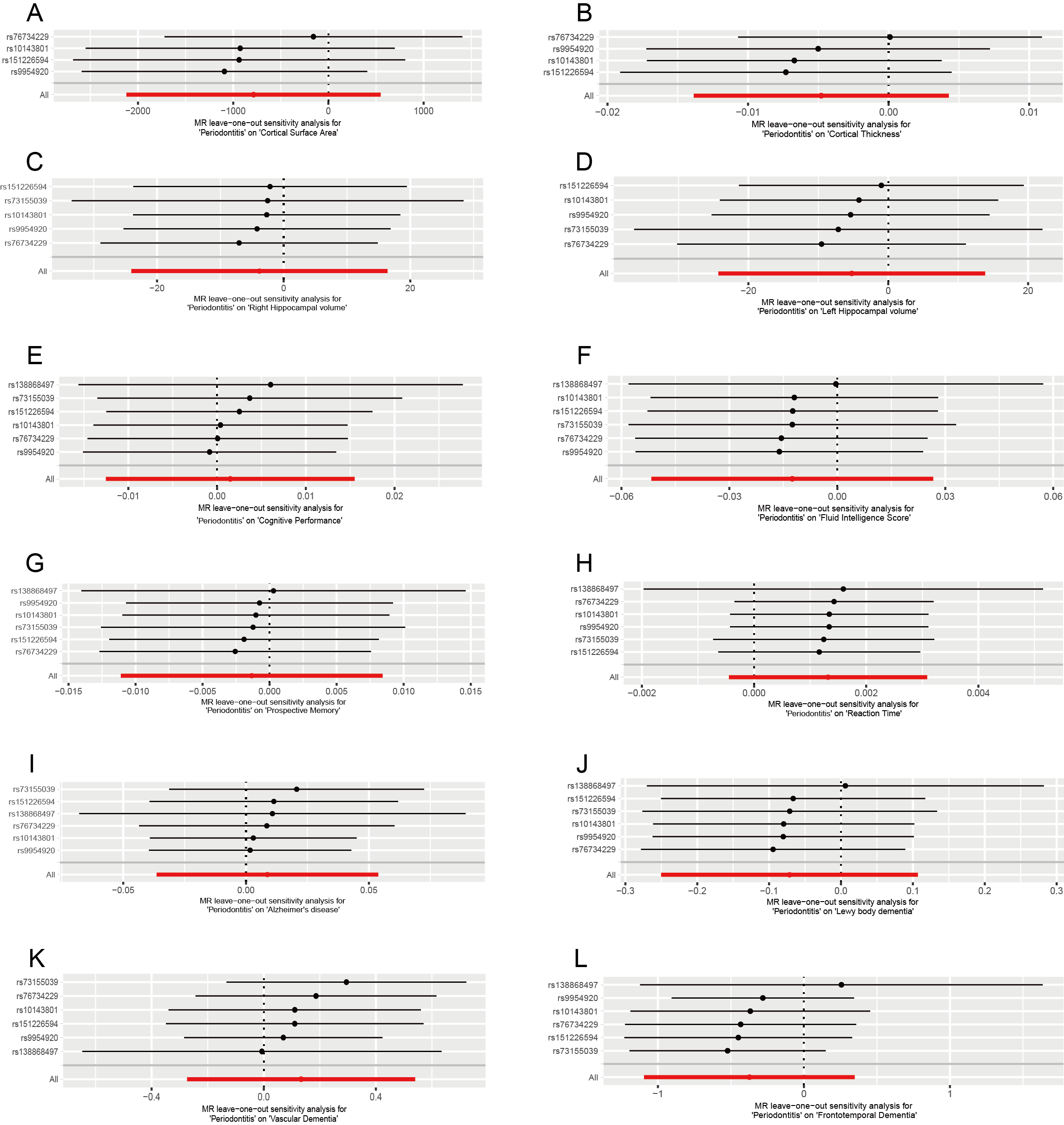


Supplementary Figure 7. Leave-one-out analysis of periodontitis with brain atrophy measures and cognitive impairment (In exploration cohort). A through L show the tests of periodontitis for different characteristics, respectively. A: Cortical surface area. B: Cortex thickness. C: Right Hippocampal volume. D: Left Hippocampal volume. E: Cognitive performance. F: Fluid intelligence score. G: Prospective memory. H: Reaction time. I: Alzheimer’s disease. J: Lewy body dementia. K: Vascular dementia. L: Frontotemporal dementia.
